# Multiple Co-Infecting Caliciviruses in Oral Fluid and Enteric Samples of Swine Detected by a Novel RT-qPCR Assay and a 3′RACE-PCR-NGS Method

**DOI:** 10.3390/v17020193

**Published:** 2025-01-30

**Authors:** Zoltán László, Péter Pankovics, Péter Urbán, Róbert Herczeg, Gyula Balka, Barbara Igriczi, Attila Cságola, Mihály Albert, Fruzsina Tóth, Gábor Reuter, Ákos Boros

**Affiliations:** 1 Department of Medical Microbiology and Immunology, Medical School, University of Pecs, 7624 Pecs, Hungary; laszlo.zoltan2@pte.hu (Z.L.); pankovics.peter@pte.hu (P.P.); toth.fruzsina1@edu.pte.hu (F.T.); reuter.gabor@pte.hu (G.R.); 2 Bioinformatics Research Group, Genomics and Bioinformatics Core Facility, János Szentagothai Research Centre, University of Pecs, 7624 Pecs, Hungary; urban.peter@pte.hu (P.U.); herczeg.robert@pte.hu (R.H.); 3 Department of Pathology, University of Veterinary Medicine, István Str 2., 1078 Budapest, Hungary; balka.gyula@univet.hu (G.B.); igriczi.barbara@univet.hu (B.I.); 4 National Laboratory of Infectious Animal Diseases, Antimicrobial Resistance, Veterinary Public Health and Food Chain Safety, 1078 Budapest, Hungary; 5 Ceva Phylaxia Ltd., 1107 Budapest, Hungary; attila.csagola@ceva.com (A.C.); mihaly.albert@ceva.com (M.A.)

**Keywords:** norovirus, sapovirus, valovirus, calicivirus, swine, saliva, oral fluid, NGS, co-infection

## Abstract

Caliciviruses including noro- and sapoviruses of family *Caliciviridae* are important enteric human and swine pathogens, while others, like valoviruses, are less known. In this study, we developed a detection and typing pipeline for the most prevalent swine enteric caliciviruses—sapovirus GIII (Sw-SaV), norovirus GII (Sw-NoV), and valovirus GI (Sw-VaV). The pipeline integrates triplex RT-qPCR, 3′RACE semi-nested PCR, and next-generation sequencing (NovaSeq, Illumina) techniques. A small-scale epidemiological investigation was conducted on archived enteric and, for the first time, on oral fluid/saliva samples of diarrheic and asymptomatic swine of varying ages from Hungary and Slovakia. In enteric samples, Sw-SaV was the most prevalent, detected in 26.26% of samples, primarily in diarrheic pigs with low Cq values, followed by Sw-NoV (2.53%) in nursery pigs. In oral fluid samples, Sw-NoV predominated (7.46%), followed by Sw-SaV (4.39%). Sw-VaVs were sporadically found in both sample types. A natural, asymptomatic Sw-SaV outbreak was retrospectively detected where the transient shedding of the virus was <2 weeks. Complete capsid sequences (*n* = 59; 43 Sw-SaV, 13 Sw-NoV, and 3 Sw-VaV) including multiple (up to five) co-infecting variants were identified. Sw-SaV sequences belong to seven genotypes, while Sw-NoV and Sw-VaV strains clustered into distinct sub-clades, highlighting the complex diversity of these enteric caliciviruses in swine.

## 1. Introduction

The family *Caliciviridae* currently consists of 11 genera from which certain members of the genus *Norovirus*, *Sapovirus*, *Valovirus*, and *Vesivirus* could infect swine hosts as well [1]. Using faecal–oral routes as the main mode of transmission, swine caliciviruses of the first three genera can be referred to as porcine enteric caliciviruses [2,3].

Noro-, sapo-, and valoviruses have a positive sense, single-stranded RNA genome of 7.3–8.5 kb in size with two (sapo- and valoviruses) or at least three (noroviruses) open reading frames (ORFs) which are flanked by short 5′/3′ untranslated regions and a 3′ poly(A) tail. The 5′ located ORF1s of all three viruses encode the non-structural proteins including the most conserved RNA-dependent-RNA polymerase (RdRp/pol) which was downstream followed by the major capsid protein (VP1) in the same (sapo- and valoviruses) or separate ORFs (noroviruses) and the minor capsid protein (VP2)-encoding ORF [1,3].

Noroviruses can infect vertebrates including humans and swine and are considered as the leading cause of acute gastroenteritis outbreaks in humans worldwide [4]. Currently, the genetically and highly diverse noroviruses are classified into 12 genogroups (GI-GX, GNA1, and GNA2) which can be further divided into at least 48 VP1 capsid-based and >60 RdRp/pol-based genotypes [5,6,7]. Co-infections of different genogroups/types have been reported sporadically in humans but have not been detected in swine [8,9]. From the known capsid genogroups, GII is medically/epidemiologically the most important in humans and also the most commonly found genogroup in swine. Among pigs, the predominance of three phylogenetically related “swine norovirus GII genotypes” (GII.11, GII.18, and GII.19) could be observed, but with low numbers of capsid sequences (*n =* 13) available ([2,4,10,11,12,13], https://ncbi.nlm.nih.gov/labs/virus/vssi/#/, accessed on 30 November 2021). Noroviruses have been detected predominantly in asymptomatic and, to a lesser extent, in diarrheic swine across all age groups from suckling to finisher pigs. The overall detection rate was low (<16.6%), with a higher prevalence generally observed in older animals (>90 days) [6,10,12,14].

To our current knowledge, sapoviruses are even more genetically diverse than noroviruses and can infect both swine and humans. Currently, sapoviruses are classified into 19 genogroups (GI–GXIX) and at least 52 VP1 capsid-based genotypes from which 8 genogroups (GIII, GV–GXI) have been commonly detected in pigs with the predominance of GIII [10,15,16,17,18]. GIII sapoviruses contain several unnumbered genotypes (>11) with high intra/inter-genotypic diversity found exclusively in pigs [16]. Sapoviruses could be present in pigs of all age groups but most frequently found during the post-weaning period in both asymptomatic and diarrheic animals and sometimes as a part of an outbreak [10,17]. Co-infections with other enteric caliciviruses and with different types in swine were reported only sporadically [10,15].

The first valoviruses of the genus *Valovirus* were described as St-Valérien-like caliciviruses in Canada in 2009 from enteric samples of asymptomatic finisher pigs [19]. Later, closely related viruses of the same genus were also detected in the USA, Japan, and Italy, exclusively from faecal samples of older (>4 months old) asymptomatic swine with variable prevalence (2.6% to 23.8%) [20,21,22]. Current knowledge about the prevalence, pathogenesis, and genomic diversity of swine valoviruses is still limited; for example, to date, only six complete genomes and a further four complete VP1 sequences have been known and only a single genogroup (GI) and genotype (GI.1) of swine valovirus have been identified [1,21].

Oral fluid/saliva samples from swine are commonly used for the detection of predominantly respiratory viruses [23,24,25,26]; however, their application for detecting enteric caliciviruses has not been previously reported.

Although several generic oligonucleotide primers, such as p289/p290 and JV12Y/JV13I, have been widely used for calicivirus detection, one-step RT-qPCR assays remain uncommon, particularly for porcine enteric caliciviruses [6,10,27,28,29]. Furthermore, most studies still rely on conventional RT-PCR and Sanger sequencing for detection and genotyping, while next-generation sequencing (NGS) techniques, capable of identifying co-infecting types, are not yet widely adopted (e.g., [9,30]). Therefore, our aims were (i) to develop a pipeline which consists of a novel, one-step triplex RT-qPCR assay and a 3′RACE semi-nested-PCR coupled NGS-based method for the detection and capsid-based typing of the most common porcine enteric caliciviruses of sapovirus GIII, norovirus GII[P] and valovirus GI. As well as to (ii) test the pipeline in enteric and oral fluid samples of swine and (iii) analyse the epidemiological and sequence data.

## 2. Materials and Methods

### 2.1. Design and Setup of the Hydrolysis Probe-Based Triplex RT-qPCR Assay

**Figure 1:**
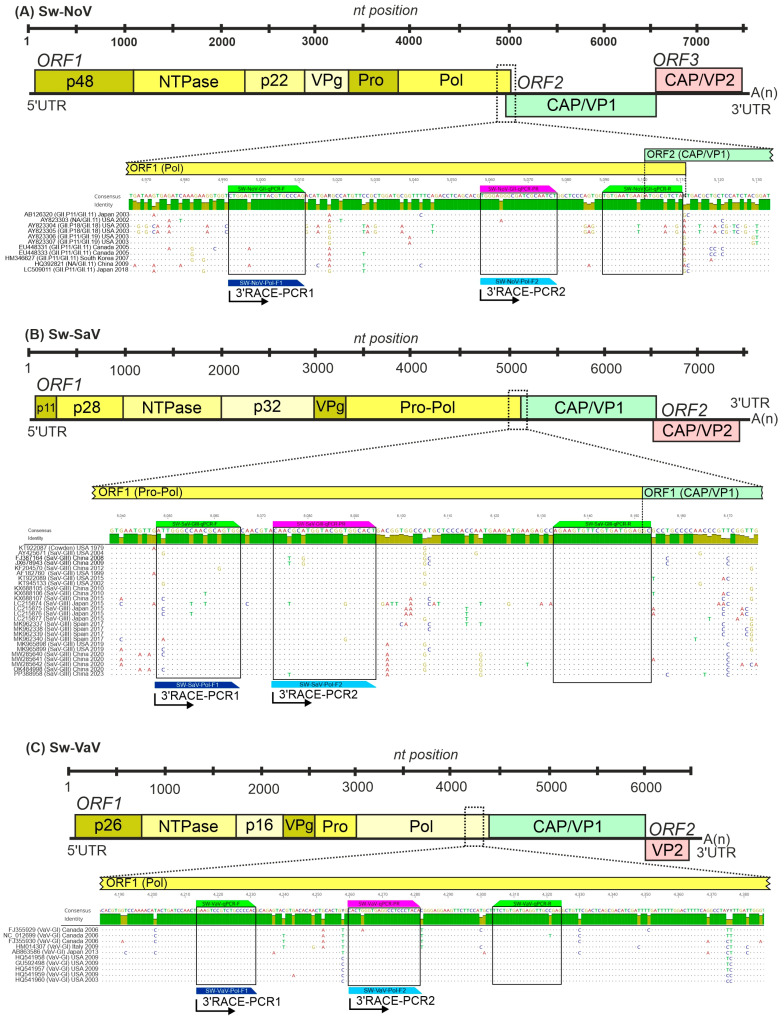
Nucleotide (nt) sequence alignments of the junctions of polymerase (Pol) and capsid (CAP) encoding genome regions swine norovirus GII (Sw-NoV, (**A**)), swine sapovirus GIII (Sw-SaV, (**B**)) and swine valovirus GI (Sw-VaV, (**C**)) sequences with the binding sites of oligonucleotide primers (green) and probes (magenta) of the RT-qPCR assay used in this study. Note that: only those of the Sw-SaV-GIII sequences were included in this representative alignment which shows a difference at the primer/probe binding region. The binding sites of F1 and F2 forward primers (which have identical sequences as the forward and probe oligonucleotides of the qPCR assays, see Table 1) used for 3′RACE semi-nested RT-PCR reactions were also indicated with dark and light blue boxes, respectively. The locations of the aligned regions including the primer/probe binding sites are marked with dotted lines in the schematic genome maps and black boxes in the alignment, respectively. The identity graphs above the alignments show identical (green bars) and moderately variable (pale yellow bars) nts. Only nts different from the consensus sequence are shown as letters with base-specific colours (C = blue, A = red, T = green and G = pale yellow) in the alignments.

All publicly available, >3000 nucleotides (nt)-long swine norovirus (Sw-NoV, *n =* 12), swine sapovirus GIII (Sw-SaV-GIII, *n =* 34) and swine valovirus (Sw-VaV, *n =* 10) sequences were downloaded from the virus database of National Centre for Biotechnology Information (NCBI, https://ncbi.nlm.nih.gov/labs/virus/vssi/#/, accessed on 30 November 2021). The downloaded sequences of the same virus were aligned separately using the MUSCLE alignment tool of Geneious Prime ver. 2021.2.2 (Biomatters) [31] and the generated multiple alignments were used for the final design of virus-specific oligonucleotide primer pairs and hydrolysis probes. Multiple different primer/probe sequences were compared to the non-redundant database of GenBank by BLASTn-search in silico to test non-specific binding (Table 1, Figure 1). To create a triplex assay, the 5′ fluorophores of the hydrolysis probes with non-overlapping peak emission wavelengths and adequate quenchers, were selected using PrimeTime^®^ Multiplex Dye Selection Tool of Integrated DNA Technologies (IDT, Coralville, IO, USA, https://eu.idtdna.com/site/order/qpcr/primetimeprobes/multiplex, accessed on 6 December 2021) (Table 1). The designed primers and probes (either separately or as an assay-specific mixture) were synthesised and shipped lyophilized by the company IDT. Oligonucleotides were resuspended with nuclease-free water to acquire stock solutions of either 50 µM (separate primers) and 2.5 µM (separate probes) or 40× (assay-specific mixtures) which were divided into smaller (30–50 µL) aliquots and stored in the dark at −20 °C until use.

#### 2.1.1. Production of Virus-Specific RNA Standards

For the optimisation of the singleplex/triplex RT-qPCR reactions and for assessing the analytical performance of the assays, RNA standards were in vitro produced using three selected representative sequences of swine norovirus GII (strain Sw/NLV/Sw918/1997/JP; AB074893), swine sapovirus GIII (strain Ishi-Im1-3; LC215875) and swine valovirus GI (strain 25A/IT/09; HM014307) from the GenBank (Table S1). The 512-nt-long regions which contain the primer/probe binding sites of the assays were synthesised and ligated into a pUC57-Simple vector with a T7 promoter sequence by BioCat GmbH (Heidelberg, Germany). The vectors were electroporated into DH5-Alpha-type competent *Escherichia coli* cells (Merck, Darmstadt, Germany) and cultured overnight in 100 µg/mL ampicillin-containing Luria–Bertani (LB) media. The cultured vectors were purified by Zyppy Plasmid Miniprep Kit (Zymo Research, Irvine, CA, USA) according to the manufacturer’s instructions. After the measurement of concentration and purity with NanoDrop 2000 (Thermo-Fisher) we linearized all vectors by EcoRI restriction enzyme (Thermo-Fisher) according to the manufacturer’s instructions. The inserts of the selected purified plasmids were sequenced directly by Sanger sequencing. The 487-nt-long RNA standards were in vitro synthesised from all three linearized vectors using the TranscriptAid T7 High Yield Transcription Kit (Thermo-Fisher) according to the protocol provided by the manufacturer. The RNA products were digested with the TURBO™ DNase enzyme (Thermo-Fisher) and extracted by TRI^®^ Reagent (MRC, Cincinnati, OH, USA) according to the manufacturer’s instructions. The presence of target RNA and vector DNA contaminations of the RNA extracts were checked with RT-qPCR with or without RT enzyme. NanoDrop 2000 and Qubit 4 Fluorometer (Thermo-Fisher) were used to measure the concentration and purity of the in vitro transcribed RNA standards. Based on the molecular weights and the measured concentration the copy numbers of the RNA standards were calculated. Ten-fold serial dilutions of mixed RNA standards were produced with nuclease-free water (NFW) for analytical performance assays.

#### 2.1.2. RT-qPCR Reaction Conditions and qPCR Data Analyses

For all RT-qPCR reactions, the Luna^®^ Universal Probe qPCR Master Mix (New England Biolabs, Ipswich, MA, USA) was used according to the manufacturer’s instructions with 40× assay-specific primer/probe mixtures (0.5 µL/reaction) with final concentrations of 500nM of all primers and 250 nM of each of the probes in a final volume of 20 µL. In epidemiological investigations, the amount of RNA samples was 5 µL. All the reactions were conducted on either 8-well white strips (Bio-Rad Laboratories, Inc., Hercules, CA, USA) or 96-well white plates (BIOplastics, Landgraaf, The Netherlands) and run on a CFX96 Touch Real-Time PCR Detection System (Bio-Rad Laboratories, Inc., USA). The thermal programme consisted of the following steps: Reverse Transcription: 55 °C-10 min, Pre-Incubation: 95 °C-1 min, Amplification: [95 °C-10 s, 59 °C-30 s] × 40/44 repeats, Final cooling: 40 °C-30 s. The fluorescence signals in three channels (FAM, VIC/SUN and Cy5) were measured at the end of every amplification step. Nuclease-free water (NFW) was used as non-template control. The data analyses were conducted with the Bio-Rad CFX Maestro 2.2 ver. 5.2.008.0222 software (Bio-Rad Laboratories, Inc., USA) applying automatic baseline detection and manual thresholds of 500, 400 and 150 for Sw-SaV/6-FAM, Sw-NoV/SUN and Sw-VaV/Cy5, respectively. The amplification efficiencies, slope and correlation coefficient values were calculated automatically by the software Bio-Rad CFX Maestro 2.2 ver. 5.2.008.0222 software. The diagrams and statistical calculations (standard deviation, mean, coefficient of variation %) were conducted in Excel ver. 16.0.17928.20114 of Microsoft 365. For data visualisation CorelDraw standard 2020 software ver. 22.0.0.474 was used. Box plot diagrams of measured quantification cycle (Cq) values were created using the BoxPlotR web tool [32].

#### 2.1.3. Analytical Performance Tests of the RT-qPCR Assay

Analytical sensitivity and specificity analyses of singleplex/triplex RT-qPCR assays were carried out using 7.5 µL of 10-fold serial dilutions of mixed virus-specific RNA standards which resulted in the template concentrations ranging from 1 × 10^8^ to 1 × 10^0^ copies of each standard in the final reaction volume of 20 µL. A lower limit of detection analyses of singleplex and triplex RT-qPCR assays was conducted using three final concentrations of mixed virus-specific RNA standards (1 × 10^3^, 1 × 10^2^ and 1 × 10^1^ copies/reaction of each element of the mixture) in three technical replicates. The experiment containing all three singleplex and a triplex assay in a 96-well plate format was repeated four times. Intra-assay reproducibility was tested using two final concentrations of the mixed RNA standards (1 × 10^3^ and 1 × 10^4^ copies/reaction of each component of the mixture) in three technical replicates and inter-assay variations were calculated based on the results of four independent RT-qPCR runs containing all three singleplex and a triplex assay on different days by two different persons. The reagents, qPCR plastics, reaction conditions and data analyses were identical in all experiments as described in the Section 2.1.1.

### 2.2. Background Information on Enteric and Oral Fluid Samples, Animals and Farms

A total of *n* = 198 archived enteric and *n* = 228 oral fluid samples were used for epidemiological investigations (Figure S1, Tables S2 and S3). The enteric samples (*n =* 79 faecal and *n =* 119 rectal swabs) originated from non-diarrheic (*n =* 135) and diarrheic (*n =* 63) swine of various ages (Table S2). Faecal samples were carefully collected from the animals’ flooring by competent people with care to prevent any possible contamination. Specimens were collected between 2008 and 2022 from *n =* 25 different large-scale industrial swine farms located in various geographical locations across Hungary (Figure S1, Table S2). A total of *n =* 336 rectal swab samples were collected from *n =* 42 asymptomatic animals first at the age of ≈21 days (D21) and then two weeks later (D35) after that every week during six consecutive weeks (D42–D77) for a follow-up investigation. The sampled animals were kept together in a freshly established swine housing unit on a large-scale industrial swine farm in Mohács, Hungary. No visible enteric symptoms were observable in the pen during the sampling period. Faecal samples were taken from the flooring underneath the animals into sterile containers. Rectal swabs were collected into individual tubes using separate polyester-tipped swabs. Faecal/rectal samples were then re-suspended in 500–1000 µL sterile 0.1 M phosphate-buffered saline (PBS) and stored at −80 °C until RNA extraction. Previous laboratory tests were not conducted on diarrheic cases.

Oral fluid samples of asymptomatic, 2–20-week-old swine were collected from *n =* 24 large-scale pig herds across Hungary and one farm (Nagyhegyes) in the neighbouring country, Slovakia between 2020 and 2022 (Figure S1), as a part of an active surveillance sampling programme. Oral fluid samples were collected using a piece of cotton rope which was hung in each pen for multiple pigs to chew on them. After 15–20 min, the ropes were removed and then the liquid from each piece was squeezed into individual plastic tubes. Therefore, all oral fluid samples were considered pooled samples of a given pen. More details about the animals and the sampling protocol can be found in the previous studies [24,25].

For epidemiological investigations, all the sampled animals were retrospectively classified into four arbitrary age groups: Suckling pigs (1–20 days old), Nursery pigs (21–77 days old), Fattening pigs (78–140 days old) and Sows (≥141 days old) (Tables S2 and S3).

### 2.3. Nucleic Acid Isolation from Enteric and Oral Fluid Samples

Total RNA was extracted from 150 µL of resuspended faecal/rectal swab samples using TRI Reagent (Molecular Research Center, Cincinnati, OH, USA) according to the instructions of the manufacturer and eluted in 50 µL nuclease-free water. The oral fluid samples were centrifuged shortly (300× *g* for 5 min) before extraction. Total RNA was extracted from 200 µL of oral fluid supernatants by QIAcube automated nucleic acid extractor (Qiagen, Hilden, Germany) using the QIAamp cador Pathogen Mini Kit (Qiagen) according to the manufacturer’s protocol. All the extracted RNA samples were stored at −80 °C until further experiments.

### 2.4. 3′RACE Semi-Nested RT-PCR and Sanger Sequencing

For 3′ Rapid Amplification of cDNA ends in semi-nested RT-PCR (3′RACE-snPCR) reactions 5-5 µL of total RNA was converted to cDNA using Oligo dT-Anchor-Adapter primer (Table 1) and MAXIMA H-minus RT enzyme (Thermo-Fisher, Waltham, MA, USA) in a reverse transcription (RT) reaction in the total volume of 20 µL according to the manufacturer’s instructions. The RT reaction product was then digested with 5U of RNAse-H enzyme (New England Biolabs, Ipswich, MA, USA) and incubated at 37 °C for 20 min.

The digested cDNA samples were used for semi-nested PCR reactions with two virus-specific outer and inner forward primers and a universal Adapter reverse primer (Table 1, Figure 1). The outer sense primer used in the 1st PCR round is the same as the forward primer of the corresponding Sw-SaV, Sw-NoV or Sw-VaV qPCR assay while the sequence of the inner sense primer used in the 2nd PCR round is identical to the probe (without the quencher/fluorophore) of the same qPCR assay (Table 1, Figure 1, Tables S2 and S3). For 3′RACE-snPCR the PCR reactions GoTaq^®^ Long PCR Master Mix (Promega, Madison, WI, USA) was used according to the instructions of the manufacturer. The thermal programme of the 1st PCR round contained 35 repeats while the 2nd PCR round had 25 cycles. With this 3′RACE snPCR method 2150-2570-bp-long genome regions of the investigated caliciviruses can be amplified (Figure 1, Table 1).

For verification 3′RACE RT-PCR reactions the RT reaction conditions are the same as described above but the PCR was performed with a DreamTaq polymerase (Thermo-Fisher, Waltham, MA, USA) enzyme with variant-specific forward primers and the same reaction conditions described previously [33,34,35].

Separate rooms with dedicated laboratory equipment were used for NA isolations, RT and PCR reagent preparations, qPCR/3′RACE PCR setup, PCR amplification, library preparations and Illumina sequencing to prevent contamination. The surfaces and equipment were cleaned with freshly prepared 3% hydrogen peroxide solution after every experiment. Multiple no-template controls (NTCs) in the semi-nested reactions were also used to monitor the presence of any contamination.

Selected 3′RACE (sn)PCR products were purified by GeneJET PCR Purification Kit (Thermo-Fisher, Waltham, MA, USA) with a standard protocol of the kit and sequenced directly from the forward direction using the BigDye™ Terminator v1.1 Cycle Sequencing Kit (Thermo-Fisher) according to the manufacturer’s instructions and run on an automated ABI 3500 Genetic Analyzer (Applied Biosystems, Waltham, MA, USA). For the analyses of Sanger-sequencing data Chromas Ver. 2.6.6, Geneious Prime ver. 2024.0.7 (Biomatters, Auckland, New Zealand) and NCBI-BLASTn software ver 2.13.0 were used.

### 2.5. Next-Generation Sequencing and Data Analysis Pipeline

Selected 2nd round products of 3′RACE-snPCR reactions were first purified using GeneJET PCR Purification Kit (Thermo-Fisher, Waltham, MA, USA) according to the instructions of the manufacturer. The concentrations of purified double-stranded (ds)DNA in the eluted samples were measured with a NanoDrop 2000 spectrophotometer and Qubit 4 Fluorometer (Thermo-Fisher, Waltham, MA, USA). Selected sapo-, noro and valovirus 3′RACE-snPCR products were pooled with pairs (either sapo-norovirus or sapo-valovirus) in quantity-ratio of 1:1 (≈100 ng each) (Table S4). In the case of pools and individual samples total of ≈200 ng dsDNA (PCR product) in a final volume of 20 µL was used for library preparations.

The library for Illumina sequencing was prepared using the NEBNext Ultra II FS DNA Library Prep Kit for Illumina (NEB, Ipswich, MA, USA). Briefly, PCR products were fragmented, end prepped and adapter ligated. Then, size selection was performed using magnetic beads to select 250–300 bp insert size fragments. Finally, the library was amplified according to the manufacturer’s instructions. The quality of the library was checked on 4200 TapeSation System using D1000 Screen Tape (Agilent Technologies, Palo Alto, CA, USA), and the quantity was measured on Qubit 4.0 (Thermo-Fisher, Waltham, MA, USA). Illumina sequencing was performed on NovaSeq 6000/X Plus instruments (Illumina, San Diego, CA, USA) with a 2 × 151 run configuration. The sequencing depth was set between 50.000.000 and 1.000.000 reads (Table S4). Details of the bioinformatics pipeline used for the NGS data analyses can be found in Figure S2. NGS reads were quality checked and the adapters were trimmed by fastp 0.21.0v [36] with the following parameter settings: −q 25 −l 40, and every other parameter was used with the default values. For positive selection of the reads a reference database of *n =* 226 caliciviruses including the prototype strains of NoV GII from Chhabra et al. [5] and all known swine noro-, sapo- and valovirus strains with >3000 nt long 3′ end genomic sequence were created. The nt sequences were downloaded from the GenBank and the NCBI virus databases (https://ncbi.nlm.nih.gov/labs/virus/vssi/#/, accessed on 30 November 2021). Sequence regions upstream from the end of the target sites of applied forward primers of the 2nd PCR round of 3′RACE-snPCR as well as the 3′ polyA tails were removed from the sequences of the reference database. Reads were analysed with Local BLAST (blastn 2.15.0+) using a reference database and only reads which showed >70% nt identity (<0.1 E-value) to any sequences of the reference database were used for further bioinformatics analyses and referred to as positively selected reads (Figure S2).

Two bioinformatics data analysis approaches were used. First, the positively selected reads were mapped against the reference genomes of porcine sapovirus GIII (strain Cowden I, AF182760), porcine norovirus GII.11 (swine/GII/OH-QW125/03/US, AY823305.2), GII.18 (strain SW/NV/swine43/JP, AB126320), GII.19 (strain swine/GII/OH-QW170/03/US, AY823306) and St-Valerien swine virus/valovirus (AB863586) using Geneious Mapper of Geneious Prime with medium sensitivity settings. Consensus sequences were generated from the reference-sequence guided alignments using the 0% threshold option (most common bases in the consensus sequence) (Figure S2). In case of the presence of uniform reads in the alignment the generated consensuses were verified by mapping the trimmed/quality checked reads to them with strict constraints (minimum overlap between reads: 50 nt, minimum overlap identity: 95%, maximum mismatch per read: 5%, max gap size 3, word length: 35, Index word length:15) (Figures S2 and S3).

In case of the presence of diverse reads in the reference-sequence guided alignments a de novo assembly-based approach was used, which contained the following read number reduction steps: the positively selected paired-end reads were first merged using BBMerge and duplicated reads were removed with Dedupe Duplicate Read Remover 38.84 in Geneious Prime. Contigs were then de novo assembled using Geneious assembler. To rule out the in silico generation of chimeric sequences originating from different viruses/variants presented in the same sample strict constraints (minimum overlap between reads: 50 nt, minimum overlap identity: 95%, maximum mismatch per read: 5%, max gap size 3, word length: 35, Index word length: 15) were applied (Figure S2). The generated >1500-nt-long contigs were searched against a non-redundant database of NCBI using BLASTn to verify the presence of calicivirus sequences. All >1500-nt-long sapo-, noro-, or valovirus contigs were used as reference sequences and QC/Trim reads were mapped against them (Map Reads to Reference) using Geneious Mapper with strict constraints: minimum overlap between reads: 50 nt, minimum overlap identity: 97%, maximum mismatch per read: 3%, max gap size 0, word length: 35, Index word length: 15 (Figure S2). Consensus sequences were generated from the reference-guided alignments using the 0% threshold option (the most common bases in the consensus sequence). If the generated consensus sequences were not reaching the primer target sites at the 5′ end and/or the poly(A)-tail at the 3′ end, then those sequences were used as references in further rounds of reference-guided mapping with the same options to extend the sequence further until reaching the expected or longest possible size (Figure S2).

### 2.6. Phylogenetic and Sequence Analysis

Multiple sequence alignments of nucleotide sequences used for primer/probe design, phylogenetic analyses and sequence comparisons were generated using the online platforms of Multiple Sequence Comparison by Log-Expectation (MUSCLE, https://www.ebi.ac.uk/Tools/msa/muscle/, accessed on 30 November 2021) or CLUSTAL 2.1 (https://www.genome.jp/tools-bin/clustalw, accessed on 30 November 2021) with default parameters). GeneDoc software ver. 2.7 and Geneious Prime Ver. 2024.0.7 (Biomatters, New Zealand) were used for sequence assembly. Pairwise nucleotide identity calculations were performed using the Sequence Identity And Similarity (SIAS) web tool (http://imed.med.ucm.es/Tools/sias.html, accessed on 30 November 2021). Nucleotide sequence-based phylogenetic trees were constructed using MEGA software ver. 11.0 with the Neighbour-Joining (NJ) algorithm and the Jukes-Cantor model [37]. Noro-, and sapovirus capsid sequences were also classified by an online Norovirus typing tool (https://www.rivm.nl/mpf/typingtool/norovirus/, accessed on 23 May 2023) [38].

### 2.7. Cluster Analysis

Hierarchical clustering was executed using the hclust function on a distance matrix derived from the Clustal W-aligned VaV and NoV GII.11 nucleotide sequences. The optimal number of clusters was assessed through silhouette analysis [39], the within-cluster sum of squares (WSS) [40] and the gap statistic [41] methods. The cluster plot displays the sequences with distinct colours representing different clusters. The DIM1 and DIM2 values represent the first two dimensions (or principal components) in a dimensionality reduction analysis. DIM1 captures the greatest variance in the data, while DIM2 captures the second-largest variance. The analysis was conducted using a script written in R in the RStudio environment, which includes the “stats” (version 3.6.2) library for hierarchical clustering [42], “msa” library [43] for multiple sequence alignment, the “ape” library [44] for calculating genetic distances between sequences and phylogenetic analysis using the lowest BIC (Bayesian Information Criterion) scores model, and the “factoextra” library [45] for displaying clusters.

### 2.8. Statistical Analysis

Statistical calculations were conducted in Microsoft Excel Ver. 2409 using the Fisher Exact Test [46].

## 3. Results

### 3.1. Design and Performance of RT-qPCR Assays

**Figure 2:**
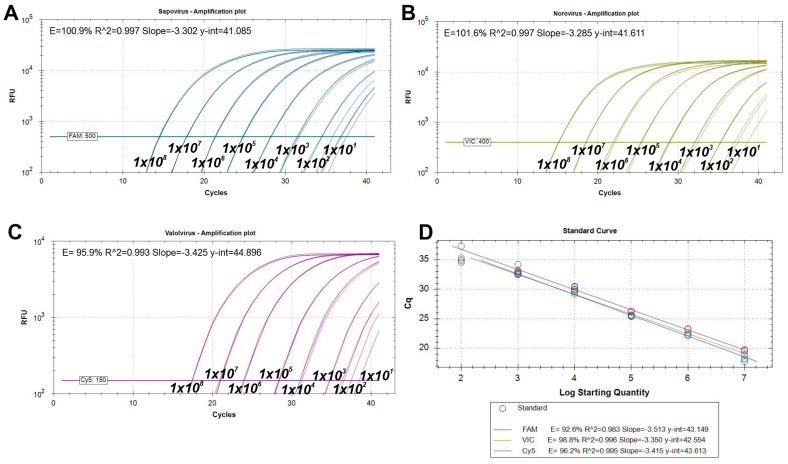
Logarithmic amplification plots and standard curves of singleplex swine sapovirus (**A**), norovirus (**B**), valovirus (**C**) and triplex assays (**D**) using 10-fold serial dilutions of mixed viral RNA standards as templates (1 ×10^8^/1 × 10^7^ to 1 × 10^1^ copies/reaction). Horizontal lines in the amplification plots indicate the arbitrary thresholds of 500, 400 and 150 for Sw-SaV/6-FAM, Sw-NoV/SUN and Sw-VaV/Cy5, respectively. Each dilution had triple technical replicates. The amplification efficiency (E), correlation coefficient (R^2^), slope and Y-intercept (y-int) values were calculated automatically by the Bio-Rad CFX Maestro 2.2 ver. 5.2.008.0222 software. RFU: relative fluorescence units.

Based on the generated nucleotide (nt) alignments of all publicly available, >3000 nt-long swine norovirus GII (Sw-NoV), swine sapovirus GIII (Sw-SaV) and swine valovirus (Sw-VaV) sequences virus-specific primers and different 5′ fluorophore-tagged (SUN for Sw-NoV, 6-FAM for Sw-SaV and Cy5 for Sw-VaV) hydrolysis probes with up to 3 mismatches were designed manually to the most conserved 3′ end of the polymerase or polymerase-capsid junctions (Table 1, Figure 1). The analytical sensitivity and specificity of the designed primer/probe sets were tested in singleplex and triplex formats using serial dilutions (1 × 10^8^–1 × 10^1^ copies/reaction) of in vitro transcribed 512-nt-long Sw-SaV, Sw-NoV and Sw-VaV RNA standards (Figure 2). The slope, correlation coefficient (R^2^) and reaction efficiency (E) values of each singleplex and triplex run are found in Figure 2.

In all three singleplex assays the lower limit of detection (LLOD) which is the lowest tested concentration which was detectable in more than 95% of the repeats was 1 × 10^2^ (100 copies/reaction) of all three targets (Table 2). While LLOD was 1 × 10^2^ in both Sw-NoV and Sw-VaV but 1 × 10^3^ in Sw-SaV (1000 copies/reaction) in a triplex assay setup (Table 2). Based on the mean Cq and standard deviation (SD) values of the lowest analysed concentration (1 × 10^1^) the cut-off Cq was arbitrarily set to 39.0 in all three targets (Figure 2, Table 2). The coefficients of variation (CV%) values of intra- and inter-assays in both analysed dilutions (1 × 10^3^ and 1 × 10^4^) ranged between 0.05 and 2.75% and 1.17–5.12%, respectively (Table S5).

### 3.2. Epidemiological Investigations of Porcine Enteric Caliciviruses in Enteric and Oral Fluid Samples

Triplex RT-qPCR-based epidemiological investigations of Sw-NoV, Sw-SaV and Sw-VaV were conducted on *n =* 198 archived enteric samples from non-diarrheic (*n =* 135) and diarrheic (*n =* 63) swine and *n =* 228 oral fluid samples from asymptomatic pigs of various ages from *n =* 46 different swine farms (Figure S1, Table 3). In the case of enteric samples, the overall most prevalent virus was found to be Sw-SaV (26.26%) followed by Sw-NoV (2.53%) and Sw-VaV (1.52%) while in the case of oral fluid samples, the overall most prevalent virus was Sw-NoV (7.46%) followed by Sw-SaV (4.39%) and Sw-VaV (0.88%) (Table 3 and Table 4). A total of 52.38% (33/63) and 14.07% (19/135) of the enteric samples from diarrheic and non-diarrheic animals were Sw-SaV positive (*p*-value < 0.001), respectively, while only 2 of the 3 Sw-VaV positive samples and only a single sample of the 5 Sw-NoV positive samples were originated from diarrheic animals (Table 3 and Table S2).

Among different age groups, the highest prevalence of Sw-SaV was measured in both enteric and oral fluid samples of nursery pigs (38.58% and 6.54%, respectively), although the virus was also sporadically detectable in both sample types of different age groups as well (Table 3 and Table 4). Interestingly, all Sw-NoV-positive enteric samples originated from nursery pigs, while the majority (*n =* 16/17) of the Sw-NoV-positive oral fluid samples were collected from fattening pigs (Table 3 and Table 4). Sw-VaV was found only in a small number of enteric samples of nursery pigs and oral fluid samples of a single nursery and fattening pig, respectively (Table 3 and Table 4). Double infections were identified in three enteric samples (*n =* 2 Sw-SaV / Sw-NoV and a single Sw-SaV/ Sw-VaV co-infection) of two suckling and a nursery pigs while triple infections were not found (Tables S2 and S3).

**Figure 3:**
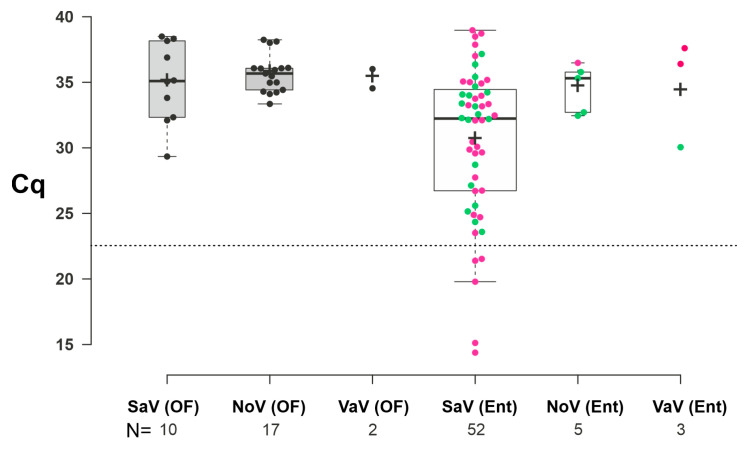
Box plot of measured Cq values of oral fluid samples (OF, grey boxes) and enteric samples (Ent., clear boxes) of swine sapovirus (SaV), norovirus (NoV) and valovirus (VaV). Centre lines show the medians; box limits indicate the 25th and 75th percentiles as determined by R software version 3.1; whiskers extend 1.5 times the interquartile range from the 25th and 75th percentiles; outliers are represented by dots; crosses represent sample means; width of the boxes is proportional to the square root of the sample size; data points are plotted as black (OF) or coloured (Ent.) circles. The number of sample points (N = x) was found below the X-axis. Cq values from enteric samples of diarrheic and non-diarrheic animals are marked with pink and green dots, respectively. The horizontal dashed line indicates the position of the Cq value of 23.00.

The comparison of Cq value distributions in enteric and oral fluid samples indicate overall higher values with the same order of magnitude (medians are >35.00) measured of Sw-NoV and Sw-VaV in both sample types and Sw-SaV in oral fluid samples, while considerably lower values (median of 32.27) could be found only in Sw-SaV in enteric samples (Figure 3). There was only a small difference in the Sw-SaV Cq values of diarrheic and non-diarrheic animals (medians/means were 32.09/30.08 and 32.56/31.31, respectively), although the lowest values (<23.00) were measured only in diarrheic cases (Figure 3, Tables S2 and S3). Note that, any role of degradation of the archived RNA samples during storage and/or during repeated freeze–thaw cycles which could cause an increase in Cq values could not be ruled out.

At the farm level *n =* 19 of the 25 (76.00%) “enteric” (i.e., farms where only enteric samples were collected) and *n =* 12 of the 24 (50.00%) “oral fluid farms” (i.e., only oral fluid samples were collected) were positive for at least one of the investigated viruses. The majority of the “enteric farms” (68.00%) were Sw-SaV positive while Sw-NoV were the most commonly detectable virus (41.7% positivity) in “oral fluid farms” (Table 5). Further details of farm-level prevalence data of the study viruses can be found in Table 5. There is one farm in Hódmezővásárhely where all three viruses were simultaneously detectable in oral fluid samples (Table 4 and Table S3).

### 3.3. Retrospective Follow-Up Investigation of Porcine Enteric Caliciviruses in a Newly Established Swine Housing Unit

**Figure 4:**
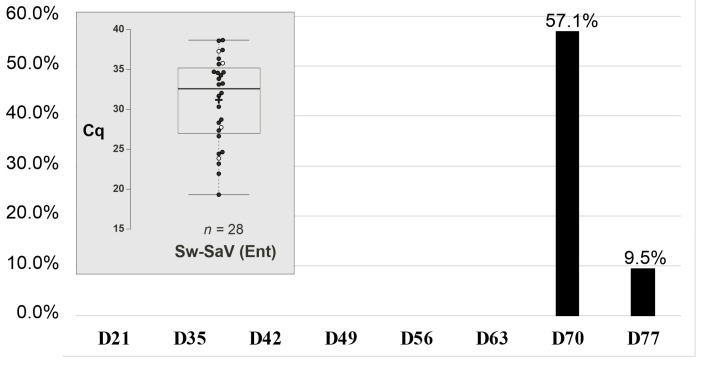
Swine sapovirus (Sw-SaV) positivity (in percentages) was detected with the triplex RT-qPCR assay in enteric samples of *n* = 42 pigs as a part of a follow-up study. D: days of age. Insert Box plot of measured Cq values of Sw-SaV in the enteric samples. Full circles: Cq values from D70 samples, empty circles: Cq values of D77 samples. Centre lines show the medians; box limits indicate the 25th and 75th percentiles as determined by R software; whiskers extend 1.5 times the interquartile range from the 25th and 75th percentiles; outliers are represented by dots; crosses represent sample means. The number of sample points (n = x) was found above the X-axis.

In a follow-up investigation total of 336 rectal swab samples from 42 asymptomatic pigs were examined retrospectively with the triplex RT-qPCR assay. The animals which were housed together in a newly established swine housing unit were sampled individually first at the relocation of the animals (age of ≈21 days, D21), then two weeks later (D35), and after that every week for six consecutive weeks (D42–D77). Neither Sw-NoV nor Sw-VaV were detectable, but overall 28 Sw-SaV positive samples were identified (Figure 4). The majority 24/28 (85.71%) of the positive samples originated from animals at the age of 70 days (D70) with a positivity rate of 57.1% (24/42 animals), while the remaining 4 positive (9.5% positivity rate) was found at D77. There were no animals which were positive in both sampling times (D70 and D77). A total of 28/42 animals (66.67%) were found to be Sw-SaV positive during the examination period (Figure 4). The measured Cq values ranged from 19.31 to 38.68 but the lowest Cq values were found among the D70 samples (Figure 4). Two of the Sw-SaV positive D70 samples (W313/9-D8 and W313/6-H4) were subject to further 3′RACE semi-nested RT-PCR reactions and NGS-based sequence analyses (see below).

### 3.4. Summary of NGS Data Analyses of 3′RACE-snPCR Products

To investigate the capsid-based genotype variance of the caliciviruses identified by our triplex RT-qPCR assay virus-specific 3′RACE semi-nested RT-PCR (3′RACE-snPCR) reactions were performed on selected qPCR positive enteric (*n =* 37/117 including *n =* 2 samples from the follow-up investigation) and oral fluid (*n =* 21/29) samples using two virus-specific forward primers (for 1st and 2nd round of PCRs) which are identical in sequence with the forward and probe of the corresponding qPCR assay (Table 1, Tables S2, S3 and S6).

From the successful 3′RACE-snPCR reactions a total of 44 2nd round 3′RACE-snPCR products (*n =* 27 Sw-SaV, *n =* 13 Sw-NoV, *n =* 4 Sw-VaV) were selected for NGS (Tables S2–S6). Ten of the 27 Sw-SaV PCR products were pooled with an equal concentration ratio with either a norovirus (*n =* 6) or valovirus (*n =* 4) product for cost reduction purposes (Table S4). The 6 sapo-norovirus, 4 sapo-valovirus pools and the individual 3′RACE-snPCR samples were sequenced using the NovaSeq platform (Illumina) where the sequencing depth has been set to either ≈50 M reads (2 samples), ≈4–6 M (7 samples) or ≈1 M reads (25 samples) (Table S4).

From the 3′RACE-snPCR products sequenced by NGS 23/27 Sw-SaV, 11/13 Sw-NoV and 3/4 Sw-VaV samples were successful (contains at least one of the investigated calicivirus sequences) (Tables S4 and S6). Calicivirus consensus sequences were generated from the positively selected NGS reads (i.e., reads with >70% nt identity to any of the known study viruses, Figure S2) first with a mapping-based approach where selected reference genomes of sapovirus GIII, norovirus GII.11/GII.18/GII.19 and valovirus GI were used as templates in reference-sequence guided alignments (Table S4). With this approach, 40 calicivirus consensus sequences could be generated. During the manual evaluation of the mapped read alignments in some of the Sw-SaV-positive 3′RACE-snPCR products, multiple, diverse reads could be observed throughout the alignments suggesting the presence of multiple sequence variants in the given samples. Therefore, in these cases de novo assembly-based approach was also applied which resulted in the generation of additional 19 consensus sequences. From the NGS data total of 59 full-length (ranged between 2128 and 2568 nt) and a single partial (3′end is missing) calicivirus consensus sequences with equivalent coverage and relatively high mean coverage values could be assembled (Table S6, Figures S2 and S3).

Based on the results of BLASTn analyses 43 sequences identified from the 25 Sw-SaV 3′RACE-snPCR products are sapovirus, 13 sequences of the 11 Sw-NoV 3′RACE-snPCR products are norovirus and 3 sequences of the 3 Sw-VaV 3′RACE-snPCR products are valovirus sequences (Table S6). Consensus sequences of the same virus show 45.00–100% nt pairwise identity to each other (Tables S7–S9). Because there are multiple consensus sequences/variants found in samples therefore besides host and virus type the total number of variants of a given sample has been included in the names of the sequences as [Var: no. of sequence variant/total variants in the sample] as well as the NGS data file ID, e.g., swine/SaV/GIII [GD0717/1-Var1/3-281] HUN/2020 (Table S6).

Selected 3′RACE sn-PCR products (*n =* 8) which contained multiple consensus sequences were also partially sequenced by the Sanger-sequencing method. All the acquired Sanger sequences showed ≥98% nt identity to the consensus sequences of the corresponding samples which had the highest mean coverage values (Table S6). In the case of one sample (Du3-SaV-3R-PCR2) which contained two Sw-SaV consensus sequences with median coverage values in a similar range (6907.0 and 6301.4, Table S6) the Sanger sequencing electropherogram contained well-recognisable mixed bases in those positions where the two Sw-SaV sequence variants also different from each other (Figure S4). Furthermore, the actual presence of some sequence variants in those of the samples which contain multiple consensus sequences was also verified 3′RACE RT-PCR with variant-specific forward primers using the original RNA samples in different RT and PCR reactions (Tables S6 and S10).

#### 3.4.1. Sequence and VP1-Based Phylogenetic Analyses of Sapovirus Sequences

A total of 11/25 Sw-SaV 3′RACE-snPCR products contained only a single sapovirus consensus sequence while further 10/25 samples had two different sequence variants, and there were additional three samples which contained 3, 4 and 5 different consensus sequences, respectively (Table S6).

**Figure 5:**
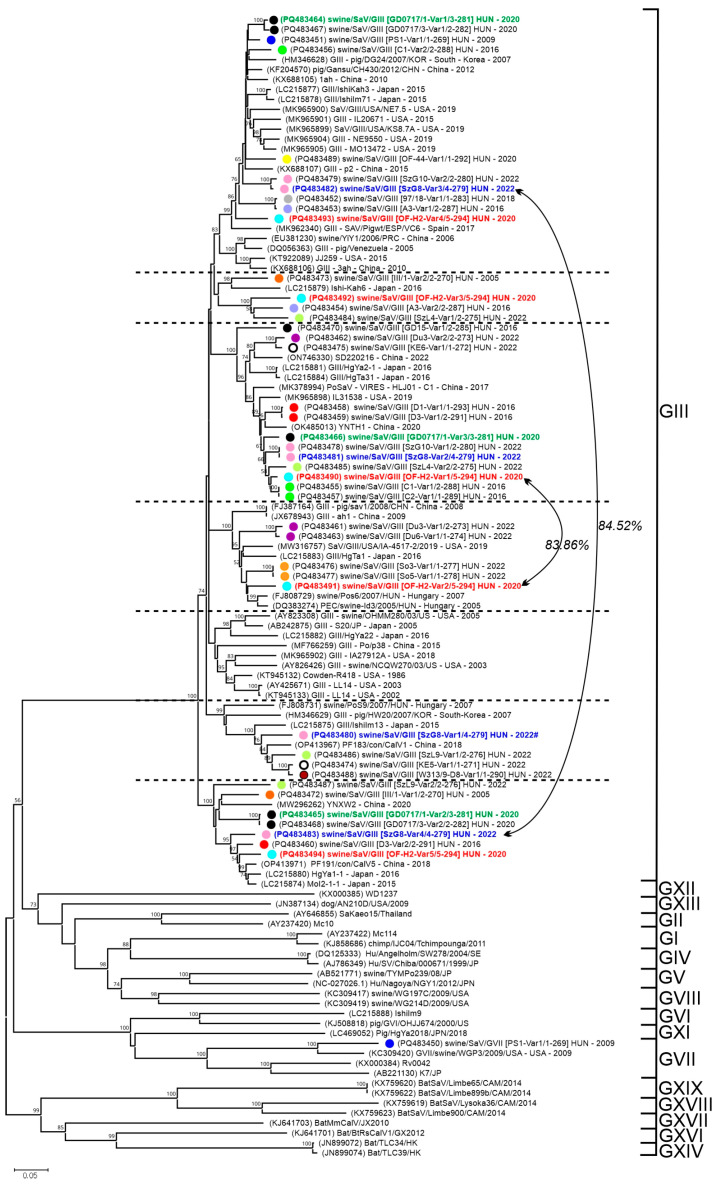
Phylogenetic analysis of sapovirus (SaV) VP1 nucleotide sequences. The Neighbour-Joining phylogenetic tree (Jukes-Cantor method, 1000 bootstrap/BS replicates, BS values less than 50 were eliminated from the tree) contains all the SaV VP1 sequences (*n* = 43) determined in this study (marked with coloured circles) together with its most closely related strains identified by BLASTn search as well as representatives of the known SaV genogroups (GI-GXIX). Horizontal dashed lines indicate the borders between presumed (based on only phylogenetic separation) genotypes. VP1 sequences from the same farm were marked with circles of identical colour. VP1 sequences from the same sample could be identified by the same sample ID found [between square brackets] in the study strain names. Examples of various VP1 sequences found in a single sample were marked with red, green or blue fonts of the strain names. Sequence variants with more than 83.1% nt identity were marked with double arrowheads. The scale bar represents the number of substitutions per site, indicating genetic distance between taxa.

The Sw-SaV VP1 sequences determined in this study show 45.00–99.87% pairwise nt identities to each other (Table S7). The phylogenetic analysis of the Sw-SaV VP1 sequences shows that all the study strains are most closely related to sequences of genogroup GIII and typed as GIII sapovirus with the Norovirus Typing Tool except one (swine/SaV/GVII [PS1-Var1/1-269] HUN/2008) which is clustered together with GVII strains (Figure 5) and show high identities (up to 84% nt and 92% aa) to the GVII sequences. The GIII study strains which show ≥77.9% nt pairwise identities in the VP1 to each other were located in 6 different clusters (Table S7, Figure 5). The VP1 sequences of the same sample are phylogenetically separated from each other and always located on different clusters. Furthermore, these VP1 sequences generally show ≤83.1% nt identities to other VP1 sequences of the same sample except for two pairs with 83.86% and 84.52% nt identities, respectively (Figure 5; Table S7).

There were certain swine farms like Ormándlak, Szigetvár and Orosháza where 3–5 Sw-SaV variants were identifiable (Figure 5, Table S6). Those of the Sw-SaV strains which have high (≥97%) VP1 sequence identities to each other show a close relationship in the VP1 tree originating from different enteric samples of the same farm (Figure 5; Table S7).

#### 3.4.2. Sequence and VP1-Based Phylogenetic Analyses of Norovirus Sequences

In the case of noroviruses, only single consensus sequences could be assembled from 9/11 3′RACE-snPCR products while the remaining two products contained two variants (Table S6). The capsid-based genotypes of the acquired noroviruses could be reliably determined by an online Norovirus typing tool with high (<68.80) BLAST score values. Norovirus variants of the same sample show ≤62.41% nt identities to each other (Table S8).

**Figure 6:**
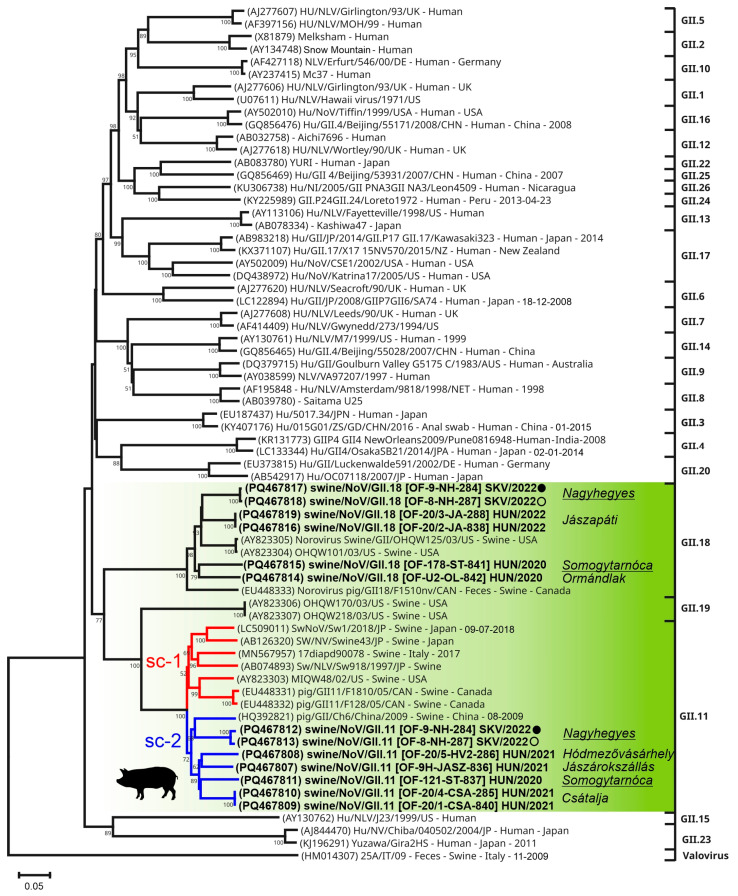
Phylogenetic analysis of full-length norovirus (NoV) VP1 nucleotide sequences. The Neighbour-Joining phylogenetic tree (Jukes-Cantor method, 1000 bootstrap/BS replicates, BS values less than 50 were eliminated from the tree) contains all the NoV VP1 sequences (*n =* 13) determined in this study (in bold) together with its most closely related strains identified by BLASTn search (including all the known swine NoV VP1 sequences) as well as representative sequences of NoV GII genotypes (GII.1-GII.26). A valovirus sequence was used as an outgroup. A main NoV lineage which contains all the known swine NoVs of genotypes GII.11, GII.18 and GII.19 was marked with a green background. Farm names in italics were found next to the study sequences. Farms where multiple types of NoVs were detected are underlined. Sequences from the same sample were marked with identical circles. Two main sub-clades (sc-1 and sc-2) of GII.11 are marked with red and blue lines. OF: oral fluid. The scale bar represents the number of substitutions per site, indicating genetic distance between taxa.

The study norovirus VP1 sequences show 64.63–100% pairwise nt identities to each other (Table S8). Phylogenetic analysis of the VP1 sequences revealed that 6/13 and 7/13 strains clustered together with swine noroviruses of GII.18 and GII.11, respectively, with >84% nt identities to sequences of the same cluster (Figure 6). The VP1 sequences of the same sample show ≤65.58% nt pairwise identities are always located on different clusters (Figure 6, Table S8). There are two farms (Nagyhegyes and Somogytarnóca) where members of two different types/clusters were parallel detectable (Figure 6). Furthermore, while the study GII.18 sequences branched together with the three previously known GII.18 strains until then, the GII.11 sequences formed two well-defined sub-clades (sc-1 and sc-2, Figure 6). One sub-clade contains the GII.11 sequences from USA, Canada, Japan and Italy the second sub-clade includes all the study GII.11 sequences and a Chinese strain (HQ392821) (Figure 6). The separation of the VP1 sequences of GII.11 into two different groups/sub-clades was also supported by the result of the cluster analysis where sequences from the VP1 phylogenetic sub-clades of GII.11 (Figure 6) were also separated into two clusters (Figure S5).

#### 3.4.3. Sequence and VP1-Based Phylogenetic Analyses of Valovirus Sequences

In the case of valoviruses, only single consensus sequences could be assembled from all three valovirus 3RACE-snPCR products from two farms which show ≤88.72% nt identities (Tables S6 and S9).

**Figure 7:**
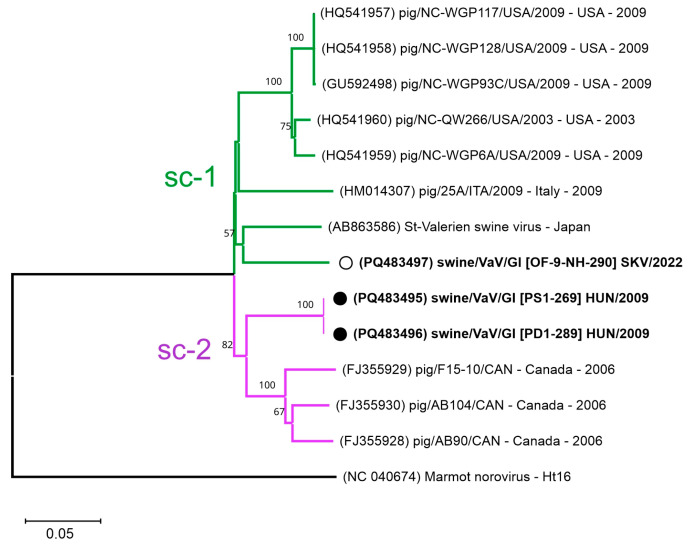
Phylogenetic analysis of full-length swine valovirus (VaV) VP1 nucleotide sequences. The Neighbour-Joining phylogenetic tree (Jukes-Cantor method, 1000 bootstrap/BS replicates, BS values less than 50 were eliminated from the tree) contains all the VaV VP1 sequences (*n =* 3) determined in this study (in bold) together with all the known swine valovirus VP1 sequences. A marmot norovirus sequence was used as an outgroup. Study sequences from enteric or oral fluid/OF samples were marked with black and empty circles, respectively. Two main sub-clades (sc-1 and sc-2) are marked with green and purple lines. The scale bar represents the number of substitutions per site, indicating genetic distance between taxa.

The VP1 sequences of study valovirus strains show 88–100% pairwise nt identities to each other (Table S9). The identical VP1 sequences originated from different enteric samples (PS1 and PD1) of the same farm collected at the same time (Table S6). The study valovirus strains from different farms are located on separate lineages in the VP1 phylogenetic tree (Figure 7). Valovirus strains from farm Pusztaföldvár (Hungary) are clustered together with strains from Canada with relatively high (>89.49%) sequence identities and formed a well-defined sub-clade while a single strain from an oral fluid sample from Slovakia are grouped together with a strain from Japan with 89.74% nt pairwise identity and formed a second sub-clade with the remaining sequences from USA and Italy (Figure 7). The same members of the two phylogenetic sub-clades were also formed in two groups in the valovirus VP1 cluster analysis (Figure S6).

## 4. Discussion

### 4.1. Evaluating the Analytical Sensitivity and Efficiency of a Novel Triplex RT-qPCR Assay

The pol or pol-cap junctions as target sites of primer-probe sets of our RT-qPCR assay are also commonly used sites for calicivirus detection [10,29]. The slope (should range between −3.100 to −3.600), correlation coefficient (R^2^, should be >0.980), reaction efficiency values (E, should be between 90 and 110%) and intra/inter-assay variations (should be <5% and <10%, respectively) of singleplex and triplex assays were found in an acceptable range indicating the optimal performances of both assay types [47,48]. Although the values could be different in other matrices. The lowest target concentration which was detectable in >95% of the repeats [47] for Sw-SaV was one magnitude higher compared to the other targets in the triplex assay which could indicate lower Sw-SaV detection sensitivity in triplex assay which could have a negative impact on the detection rate of Sw-SaV in samples with low (i.e., <1000 copies) viral loads.

### 4.2. Age-Dependent Prevalence and Mixed Infections of Porcine Enteric Caliciviruses in Enteric Samples

The overall most prevalent virus in enteric samples was Sw-SaV followed by Sw-NoV at both animal and farm levels, similar to those previously found in Europe [49,50] including Hungary [18,51]. Valoviruses were detected for the first time in Hungary in two geographically distant farms with a similarly low prevalence as found in Italy [20]. The highest prevalences of all three viruses were detected among nursery pigs. Although previous investigations also found that newly weaned/nursery pigs have the highest sapovirus infection rates [16], the noroviruses were generally detectable in older animals (>90 days) not at nursery age [10,12,14]. Furthermore, valoviruses have never been detected in young animals like nursery pigs before [19,21,22]. The discrepancies between our results and literature could be due to the relatively low number of available enteric samples from the oldest age groups (only 39 and 15 samples of fattening pigs and sows were available, respectively), although due to the limited number of epidemiological studies of noro-, and valoviruses in swine, the age-related prevalence data of these viruses could be considered as unreliable.

From the three investigated viruses only Sw-SaV was detectable in a significantly higher prevalence in diarrheic samples with the lowest measured Cq values indicating a correlation between Sw-SaV infection and diarrhoea. The association of Sw-SaV infection and gastroenteritis among swine is controversial in the literature (diarrheic cases: [52,53,54]) asymptomatic cases: [55,56], but generally higher viral loads were measured in some of the diarrheic cases [53]. Because no other enteric pathogens were investigated the association of sapovirus infection with diarrhoea is unclear in our cases.

Besides Sw-SaV, Sw-VaV is also mostly detectable in samples from diarrheic pigs, but only sporadically. As far as we know this is the first detection of valoviruses from diarrheic swine but due to the low prevalence of the virus (only 3 positive enteric samples) and the lack of additional diagnostics of other enteric pathogens the association of valovirus infection and diarrhoea is undefined. Meanwhile, Sw-NoV was found mainly in asymptomatic animals similar to those found in other studies [10]. Besides single infections, a small number of sapovirus-norovirus– and sapovirus–valovirus double infections were also identified where the latter could be a novel finding.

#### Transient Shedding and High Initial Prevalence of Swine Sapovirus in an Asymptomatic Outbreak

A naturally occurring, asymptomatic sapovirus outbreak was also retrospectively detected in a newly established swine housing unit. Sw-SaV was detectable first among 70-day-old animals with an immediate high positivity rate, which could indicate a recent introduction and rapid transmission of the virus. Sw-SaV was still detectable one week later (D77) among the animals but with a considerably lower prevalence. Unfortunately, there was no further sampling after D77, therefore the duration of the outbreak cannot be determined. There were no animals which were positive in two sampling times indicating the transient shedding (up to 14 days) of sapovirus through faeces. Similarly, short infection dynamics (up to 9 days) were found in experimentally infected gnotobiotic pigs, although longer shedding (up to 34 days) was also reported [16,57].

### 4.3. Age-Dependent Prevalence and Mixed Infections of Porcine Enteric Caliciviruses in Oral Fluid Samples

All three investigated viruses can be detectable in oral fluid samples from asymptomatic pigs of different ages with various prevalences with the predominant presence of noroviruses although, our prevalence data are limited by the small number of oral fluid samples collected from suckling pigs or sows. Fifty per cent of the “oral fluid farms” were positive for at least one virus, while there was a farm where all three viruses were simultaneously detectable. These results indicate the widespread presence and co-circulation of these viruses in investigated swine farms. There were also differences in Sw-NoV frequencies in fattening pigs for enteric vs. oral fluid samples which could be due to the lower number of available enteric samples compared to oral fluids from fattening pigs. Further studies using a higher number of samples and oral fluid-enteric sample pairs would require investigating the age-related prevalence of noroviruses.

As far as we know this is the first report of the presence of all three investigated porcine enteric caliciviruses in oral fluid samples of swine and the presence of valoviruses in Slovakia as well. Recent studies indicate that certain enteric viruses, including rota-, or noroviruses can be detectable in human saliva, the replication of noroviruses in human salivary glands and the transmission through saliva were also proved [4,7,58,59,60]. Based on our results the transmission of porcine enteric caliciviruses including noroviruses through saliva in swine should also be considered. The Cq values of all three viruses, especially in the case of Sw-SaV in oral fluid samples were generally higher than found in the enteric samples indicating a lower copy number in oral fluid samples which could be due to either the pooled form of the oral fluid samples (these samples were collected from ropes which were hanged in cages and all animals in the cages could/were chewed the rope regardless of their infection status) or the generally lower shedding of these viruses in the saliva (note that noroviruses could also be found in relatively low copy numbers in human saliva) [60], but, the environmental origin of these viruses in the mouth is also plausible, requiring experimental studies to confirm viral replication in the salivary glands of swine.

### 4.4. Exploring Hidden Variant Diversity of Porcine Enteric Caliciviruses Using Combined 3′RACE-snPCR and NGS Approaches

With our 3′RACE-snPCR method ≈2100–2500-bp-long, 3′ genomic regions including the complete capsids of the investigated caliciviruses can be amplified even when the given virus was present in a relatively low copy number (>35.00 Cq/<100 copies). Similar 3′RACE-PCR-based approaches for caliciviruses were also reported previously, although those protocols were based on cloning and/or Sanger sequencing methods [61,62]. Unfortunately, the target sites of our primers were located so close to the 3′ terminal ends of the RdRp-s (c.a 23–140 nt from the start of capsid) in all three viruses which resulted in the lack of suitable length of RdRp sequence required for reliable polymerase-type (P-type) determination in the dual nomenclature system of caliciviruses. Selected 3′RACE-snPCR products were sequenced using a short-read NGS method with different sequencing depths. Several co-infecting variants could be identified using the reference-guided (for single variants) and de novo assembly-based (for mixed variants) bioinformatics approaches. Due to the sequencing depth and the relatively high coverage the gathered data could be used for intra-host analyses as well which we did not perform in this study. Up to four or five different consensus sequences could be acquired from the pooled or single 3′RACE-snPCR products, respectively, using ≈1 M sequencing depth, which makes the cost of the NGS for a PCR product comparable to the cost of a Sanger-sequencing with primer-walking method.

The highest abundances of Sw-SaV, Sw-NoV and Sw-VaV variants/types were found in oral fluid samples. These oral fluid samples should be considered as pooled samples due to the sample collection method therefore the parallel presence of multiple Sw-SaV, Sw-NoV and Sw-VaV variants does not necessarily indicate co-infections (but also does not rule them out), rather suggesting co-circulations of these viruses/variants in majority of the investigated farms. Our findings indicate that oral fluids could be an easily collectable and cost-efficient sample type for analysing not just respiratory infections [23,24,25], but enteric caliciviruses in swine especially when NGS was also applied.

Only the most abundant genotype/sequence variant (with the highest coverage in the NGS data) could be detectable by Sanger sequencing. Traces of multiple variants/genotypes in the Sanger electropherogram could be seen as mixed bases when two variants are presented in the sample with a similar abundance. These observations indicate that if these PCR products were sequenced only with the Sanger-sequencing technique then the variant/genotype spectrum most likely would remain hidden. Therefore, for calicivirus discovery or epidemiological investigations especially in those cases when generic primer pairs are being used the use of NGS should be strongly advised.

#### 4.4.1. High Genotypic Diversity and Co-Infection Rates of Sapoviruses

Based on the results of VP1-based sequence, phylogenetic analyses and currently accepted genotype demarcation criteria of sapoviruses (≤83.1% nt identities in the VP1 between genotypes) [15] all of the identified Sw-SaV sequence variants belong to 7 different genotypes (*n =* 6 of GIII and a *n =* 1 of GVII, which could be a GIII-RdRp/GVII-capsid recombinant virus, but it was not investigated further) except two variants which have slightly higher VP1 identities to other Sw-SaV sequences of the same sample, although phylogenetically clearly separated from each other. This could suggest the need for the loosening of the currently accepted sequence-based demarcation criteria of sapoviruses and the parallel use of VP1-based phylogenetic analyses for proper typing. The identified Sw-SaV genotypes are generally widespread and frequently co-circulating in the investigated swine farms in Hungary. More than half of the analysed Sw-SaV positive samples contained more than one (up to five) variants which could indicate the high level of co-infections and considerable genetic variance of co-circulating Sw-SaV strains in the investigated pig populations. The presence of multiple genotypes of sapoviruses in swine farms has been previously reported, although with a lower level of genotype variance [16,54,63]. The highest abundance of co-infecting viruses was found in newly weaned/nursery pigs which could be due to the lack of adequate quantities of passive colostral antibodies caused by the colostrum deprivation and/or the decreased immune status due to post-weaning stress [16,64].

Some of the VP1 sequences from samples of the same farm are generally closely related to each other indicating the widespread presence of certain Sw-SaV genotypes in that farm in the year of sampling.

The identified Sw-SaV types from oral fluid samples were not separated from the strains found in enteric samples which could indicate the same tropism of enteric-origin and saliva-origin types. Unfortunately, there were no oral fluid-enteric sample pairs available for comparison analyses which could prove/disprove this hypothesis. There were no significant differences in the co-infection rates of Sw-SaV variants between diarrheic and non-diarrheic animals, although quadruple Sw-SaV variant infection was found only in one faecal sample of a diarrheic animal. Mixed Sw-SaV infections were described only sporadically [16,62], but as far as we know this is the first report of a quadruple Sw-SaV variant co-infection.

#### 4.4.2. Novel Sub-Clusters and Co-Infections of Norovirus GII.11 and GII.18

Norovirus consensus sequences could be determined only from oral fluid samples. Based on the results of phylogenetic and sequence analyses of VP1/complete capsid sequences including the Norovirus Typing Tool [38] all study norovirus strains belong to the “swine norovirus genotypes” of GII.11 and GII.18. Both Sw-NoV types were detected in several Hungarian farms and also in a farm in Slovakia which could indicate the widespread distribution and frequent co-circulation of these types in the investigated geographical areas. Our genotype-distribution/prevalence data are comparable with the information available from other parts of Europe including Italy, Belgium and Slovenia where also GII.11 was found to be the dominant type followed by GII.18 [12,48,65]. Based on the results of our phylogenetic and cluster analyses two novel, well-defined sub-clusters could be distinguishable among GII.11 viruses which could indicate a separate intra-genotypic evolutional pattern of different NoV sub-types/variants of predominant GII.11 similar to those found among the dominating GII.4 type of human noroviruses [66,67]. Furthermore, GII.11 strains from different countries are located on different lineages in the sub-cluster which could indicate the geographic separation of these strains, although the number of available sequences is still low to support this preliminary observation. The identified Sw-NoV strains from oral fluid samples were phylogenetically separated from the previously found enteric strains which could raise the possibility of different tropisms of enteric-origin and saliva-origin NoVs, but multiple additional capsids from enteric samples are needed to address this assumption.

#### 4.4.3. Genetic Divergence and Novel Sub-Clusters of Swine Valoviruses

Based on the results of our phylogenetic and cluster analyses, the Sw-VaV strains from Hungary and Slovakia form distinct lineages, as a part of two, previously unrecognisable sub-groups of swine valovirus GI. This observation suggests a more complex evolutionary pattern of GI valovirus subtypes/variants. However, due to the still low number of capsid sequences, the level of genetic diversity of valoviruses cannot be analysed.

## Figures and Tables

**Table 1:** List and characteristic features of oligonucleotide primers and probes used in this study. F: forward, R: reverse, PR: probe, RT: reverse transcription, Tm: melting temperature, RT-qPCR: singleplex/triplex RT-qPCR assays. 6-FAM: 6-Carboxyfluorescein; SUN: a fluorophore with a similar spectrum as HEX and VIC dyes. Cy5: Cyanine 5/IABkFQ/3′ Iowa Black^®^ FQ quencher; /IAbRQSp/3′ Iowa Black^®^ RQ quencher. ZEN, TAO: internal quenchers. 3′RACE: 3′ Rapid Amplification of cDNA ends. sn-PCR: semi-nested PCR, *: 3′RACE-snPCR product lengths were calculated with Adapter-1 as a reverse primer. # The primer is used only for reverse transcription reactions.

Target (Region)	Reaction Type	Oligonucleotide Primer ID	Sequence (5′–3′)	Length (nt)	Tm (°C)	PCR Product Size
Swine norovirus GII (Pol-Cap junction)	RT-qPCR	SW-NoV-GII-qPCR-F	CTG GAG TTT TAC GTG CCC AG	20	63	120 bp
		SW-NoV-GII-qPCR-PR	/SUN/TGG GAG GGC/ZEN/GAT CGC AAT CT/IABkFQ/	20	69	
		SW-NoV-GII-qPCR-R	TAG ACG CCA TCT TCA TTC ACA	21	63	
	3′RACE-snPCR1	SW-NoV-Pol-F1	CTG GAG TTT TAC GTG CCC AG	20	63	≈2570 bp *
	3′RACE-snPCR2	SW-NoV-Pol-F2	TGG GAG GGC GAT CGC AAT CT	20	69	≈2500 bp *
Swine sapovirus GIII (Pol-Cap junction)	RT-qPCR	SW-SaV-GIII-qPCR-F	ATT GGG CCA ACG CAG TGG	18	64	106 bp
		SW-SaV-GIII-qPCR-PR	/6-FAM/CAA CGC ATG/ZEN/GTA CGG TGG CAC T/IABkFQ/	22	69	
		SW-SaV-GIII-qPCR-R	GCC TCC ATC ACG AAC ACT TCT	21	64	
	3′RACE-snPCR1	SW-SaV-Pol-F1	ATT GGG CCA ACG CAG TGG	18	64	≈2300 bp *
	3′RACE-snPCR2	SW-SaV-Pol-F2	CAA CGC ATG GTA CGG TGG CAC T	22	69	≈2270 bp *
Swine valovirus GI (Pol)	RT-qPCR	SW-VaV-qPCR-F	GAA GTC CGT CTG CCC CAC	18	64	112 bp
		SW-VaV-qPCR-PR	/Cy5/CAC TGG GTG/TAO/AGG CCT CCC TAC A/IAbRQSp/	22	69	
		SW-VaV-qPCR-R	CTC GGC AAC CTC ATC ACA GAA	21	64	
	3′RACE-snPCR1	SW-VaV-Pol-F1	GAA GTC CGT CTG CCC CAC	18	64	≈2200 bp *
	3′RACE-snPCR2	SW-VaV-Pol-F2	CAC TGG GTG AGG CCT CCC TAC A	22	69	≈2150 bp *
3′ Poly(A)-tail	3′RACE-RT	Oligo dT-Anchor-Adapter #	GGC CGC GCC ACC AAT TTA AA T(15)V	36	>42	
	3′RACE-snPCR1/2	Adapter-1 *	GGC CGC GCC ACC AAT TTA AAT	21	66	

**Table 2:** Summary of the results of the lower limit of detection (LLOD) assays. Cq: quantification cycle, SD: standard deviation, Sw-SaV: swine sapovirus, Sw-NoV: swine norovirus, Sw-VaV: swine valovirus. - (-): no amplification was detectable.

Copies/ Reaction	Mean Cq (SD)						Detected/Tested (%)					
	Singleplex			Triplex			Singleplex			Triplex		
	Sw-SaV	Sw-NoV	Sw-VaV	Sw-SaV	Sw-NoV	Sw-VaV	Sw-SaV	Sw-NoV	Sw-VaV	Sw-SaV	Sw-NoV	Sw-VaV
(1 × 10^1^) 10	37.05 (0.71)	37.53 (0.09)	38.46 (0.01)	- (-)	38.30 (0.64)	38.87 (0.88)	3/10 (30)	4/10 (40)	2/10 (20)	0/10 (0)	5/10 (50)	2/10 (20)
(1 × 10^2^) 100	34.99 (0.28)	35.18 (0.42)	36.16 (0.61)	37.82 (0.53)	35.46 (0.59)	36.41 (0.99)	10/10 (100)	10/10 (100)	10/10 (100)	4/10 (40)	10/10 (100)	10/10 (100)
(1 × 10^3^) 1000	31.79 (0.24)	32.16 (0.12)	32.81 (0.25)	33.00 (0.27)	31.91 (0.20)	32.66 (0.30)	10/10 (100)	10/10 (100)	10/10 (100)	10/10 (100)	10/10 (100)	10/10 (100)

**Table 3:** RT-qPCR-based prevalence data (no. of positive/total, and percentages in brackets) of swine sapovirus (Sw-SaV), norovirus (Sw-NoV) and valovirus (Sw-VaV) of *n* = 198 enteric samples collected from diarrheic and non-diarrheic swine of different age groups. The highest prevalence values of all three viruses of different age groups were highlighted in bold.

Age Groups	Disease Status	Sw-SaV	Sw-NoV	Sw-VaV
Suckling pigs	diarrheic	1/8 (12.50%)	0/8	0/8
(1–20 days)	non-diarrheic	0/9 (0%)	0/9	0/9
	overall	1/17 (5.88%)	0/17 (0%)	0/17 (0%)
Nursery pigs	diarrheic	32/55 (58.18%)	1/55 (1.82%)	2/55 (3.64%)
(21–77 days)	non-diarrheic	17/72 (26.61%)	4/72 (5.56%)	1/72 (1.39%)
	overall	**49/127 (38.58%)**	**5/127 (3.94%)**	**3/127 (2.36%)**
Fattening pigs	diarrheic	0/0	0/0	0/0
(78–140 days)	non-diarrheic	1/39 (2.56%)	0/39	0/39
	overall	1/39 (2.56%)	0/39 (0%)	0/39 (0%)
Sows	diarrheic	0/0	0/0	0/0
(≥141 days)	non-diarrheic	1/15 (6.66%)	0/15	0/15
	overall	1/15 (6.66%)	0/15 (0%)	0/15 (0%)
**∑**		**52/198** **(26.26%)**	**5/198** **(2.53%)**	**3/198** **(1.52%)**

**Table 4:** RT-qPCR-based prevalence data of swine sapovirus (Sw-SaV), norovirus (Sw-NoV) and valovirus (Sw-VaV) of *n* = 228 oral fluid samples collected from asymptomatic swine of different age groups. The highest prevalence values of all three viruses of different age groups were highlighted in bold.

Age Groups	No. of Samples	Sw-SaV	Sw-NoV	Sw-VaV
Suckling pigs	1	0	0	0
(1–20 days)				
Nursery pigs	107	**7** **(6.54%)**	1 (0.94%)	**1** **(0.94%)**
(21–77 days)				
Fattening pigs	116	3 (2.58%)	**16** **(13.79%)**	1 (0.86%)
(78–140 days)				
Sows	4	0	0	0
(≥141 days)				
**Overall**	**228**	**10** **(4.39%)**	**17** **(7.46%)**	**2** **(0.88%)**

**Table 5:** Statistics of swine sapovirus (Sw-SaV), norovirus (Sw-NoV) and valovirus (Sw-VaV) RT-qPCR positive farms. “Enteric” and “oral fluid” indicate the sample types (either enteric or oral fluid, respectively) collected in the given farms. There were a total of 25 and 24 “enteric” and “oral fluid” farms investigated. Low, medium and high prevalence farms indicate the RT-qPCR-based prevalence of the given virus in the investigated samples of the given farm, which ranged between ≤33%, 34–66% and ≥67%, respectively.

Virus	Sample/Farm Type	Overall Pos. Farms	Low Prevalence Farms	Medium Prevalence Farms	High Prevalence Farms
Sw-SaV	“enteric”	17/25 (68.0%)	6/17 (35.3%)	7/17 (41.2%)	4/17 (23.5%)
	“oral fluid”	6/24 (25.0%)	5/6 (83.3%)	1/6 (16.7%)	0/6
Sw-NoV	“enteric”	3/25 (12.0%)	2/3 (66.7%)	0/3	1/3 (33.3%)
	“oral fluid”	10/24 (41.7%)	9/10 (90.0%)	1/10 (10%)	0/10
Sw-VaV	“enteric”	2/25 (8.0%)	1/2 (50.0%)	0/2	1/2 (50.0%)
	“oral fluid”	2/24 (8.3%)	2/2 (100%)	0/2	0/2

## Data Availability

The next-generation sequencing data were found in the SRA database of NCBI under BioProject ID of: PRJNA1177410. The generated consensus sequences from the two approaches were uploaded to GenBank under the accession numbers PQ483450–PQ483494 (swine sapovirus sequences), PQ467807–PQ467819 (swine norovirus sequences) and PQ483495–PQ483497 (swine valovirus sequences) and used for further phylogenetic and sequence analyses.

## Supplementary Materials

The following supporting information can be downloaded at: https://www.mdpi.com/article/10.3390/v17020193/s1, Figure S1: Localizations of swine farms involved in epidemiological investigations; Figure S2: Summary of the next-generation sequencing data analyses of 3′RACE semi-nested PCR products; Figure S3: Coverage maps of swine sapovirus, swine norovirus and swine valovirus consensus sequences; Figure S4: Comparison of an NGS-based and the Sanger-based sequences of the same PCR product; Figure S5: Cluster analysis of VP1 nucleotide sequences of swine GII.11 noroviruses; Figure S6: Cluster analysis of VP1 nucleotide sequences of swine GI valoviruses; Table S1: Features of genome regions of selected sapo-, noro-, and valovirus reference strains used to produce RNA standards; Table S2: Features enteric samples, summaries of the results of triplex RT-qPCR assays and 3′RACE semi-nested PCR reactions; Table S3: Features oral fluid samples, summaries of the results of triplex RT-qPCR assays and 3′RACE semi-nested PCR reactions; Table S4: Summary of the results of next-generation sequencing reactions of 3′RACE semi-nested PCR products; Table S5: Summary of the results of intra- and inter assay variations; Table S6: Features of the 3′RACE PCR products and assembled consensus sequences; Table S7: Pairwise nucleotide identity values between complete sapovirus consensus and VP1 sequences; Table S8: Pairwise nucleotide identity values between complete norovirus consensus and VP1 sequences; Table S9: Pairwise nucleotide identity values between complete valovirus consensus and VP1 sequences; Table S10: List and features of oligonucleotide primers used for the verification 3′RACE RT-PCR reactions.
