# Supplementary material for: Multiple Co-Infecting Caliciviruses in Oral Fluid and Enteric Samples of Swine Detected by a Novel RT-qPCR Assay and a 3′RACE-PCR-NGS Method
Source: Viruses. 2025 Jan 30;17(2):193. doi: 10.3390/v17020193 (PMC11860220; doi:10.3390/v17020193)
Supplement: Supplementary file 1 [file viruses-17-00193-s001.zip › viruses-3439014-supplementary.pdf]

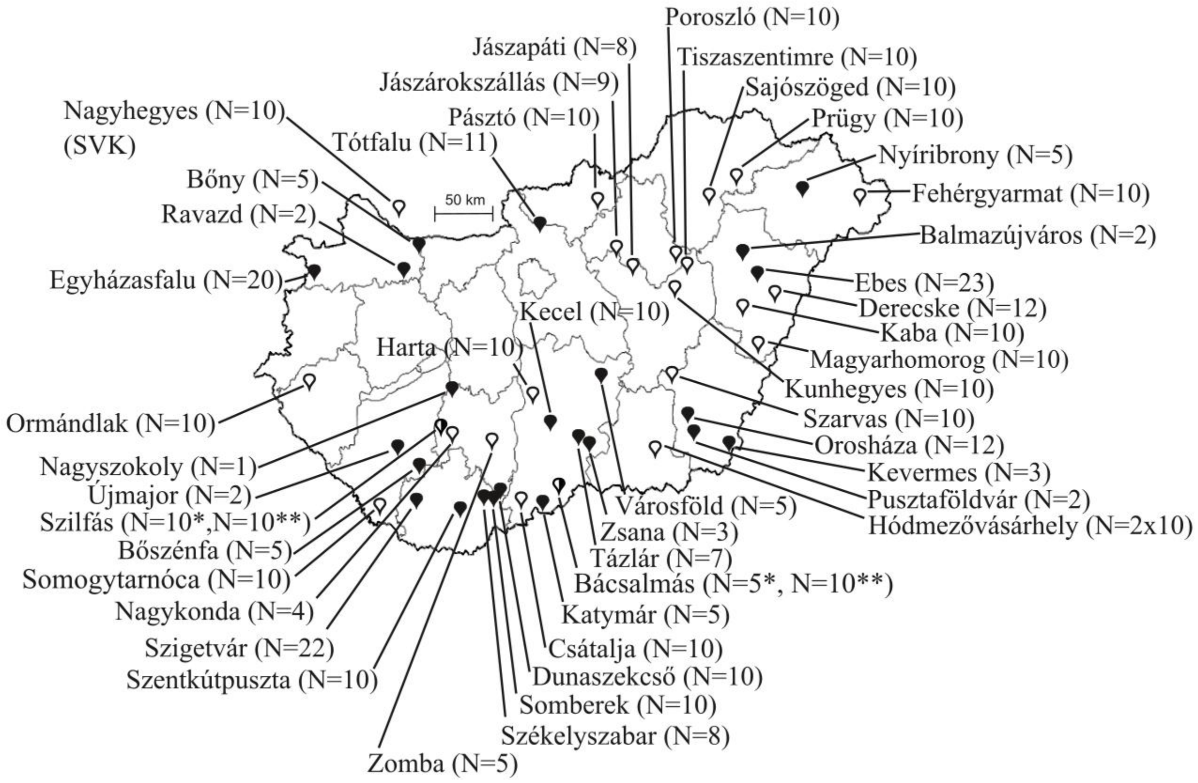

Figure S1: Localizations of the total of N=49 swine farms involved in the RT-qPCR-based epidemiological investigation of swine sapovirus GIII, norovirus GII and valovirus GI in N=198 enteric (dark tags in the map, * in sample numbers) and N=228 oral fluid/saliva samples (clear tags in the map, ** in sample numbers) of diarrheic and asymptomatic swine.

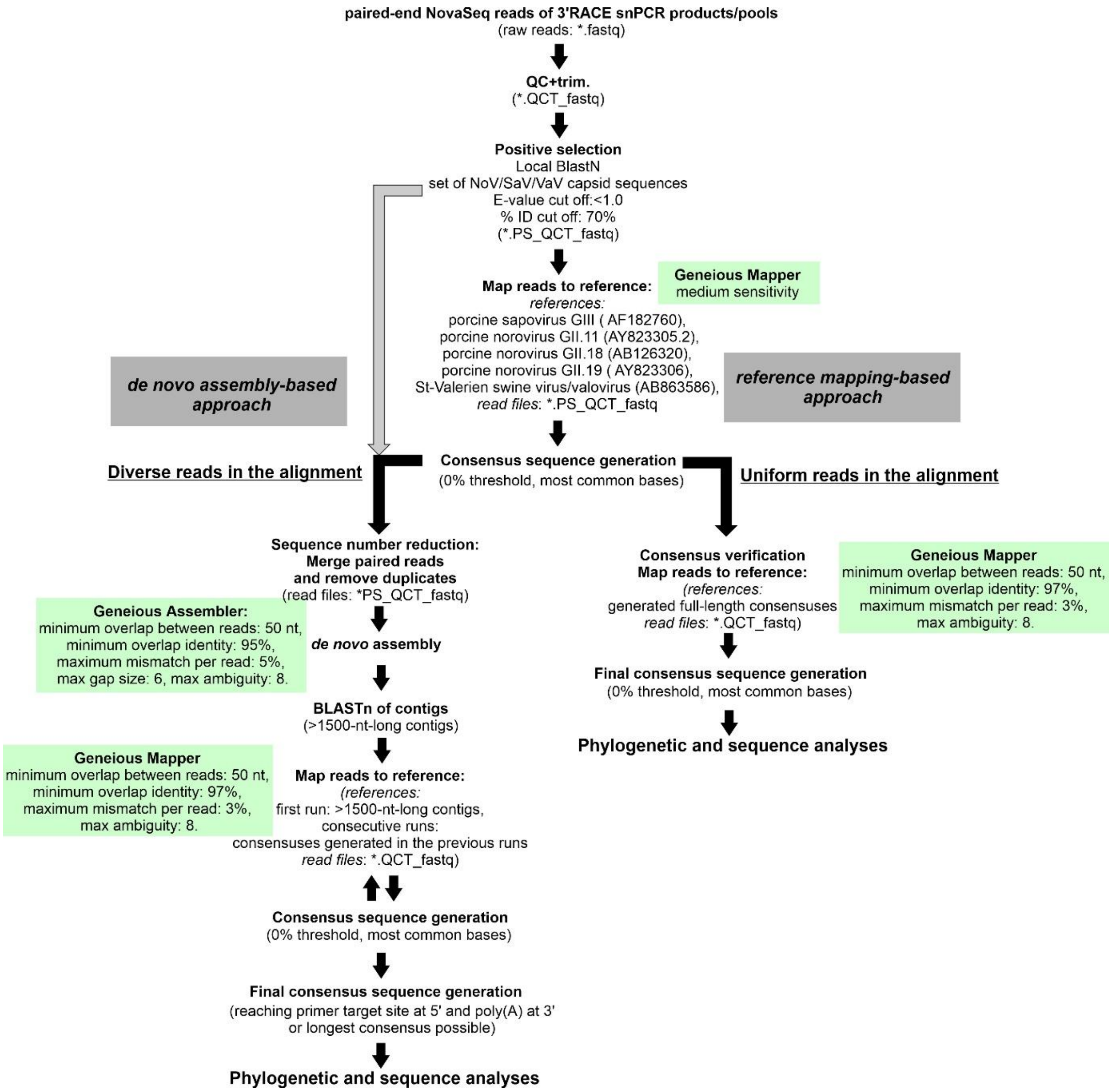

Figure S2: Summary of the next-generation sequencing data analyses of 3'RACE semi-nested PCR products using two, *de novo* assembly-based-, and reference mapping-based approaches. QC: quality check. Trim: adapter sequence trimming. Additional background information related to this data analysis pipeline can be found in Figure S3 and Table S6.

(S3-A) Sw-SaV consensus coverage maps:

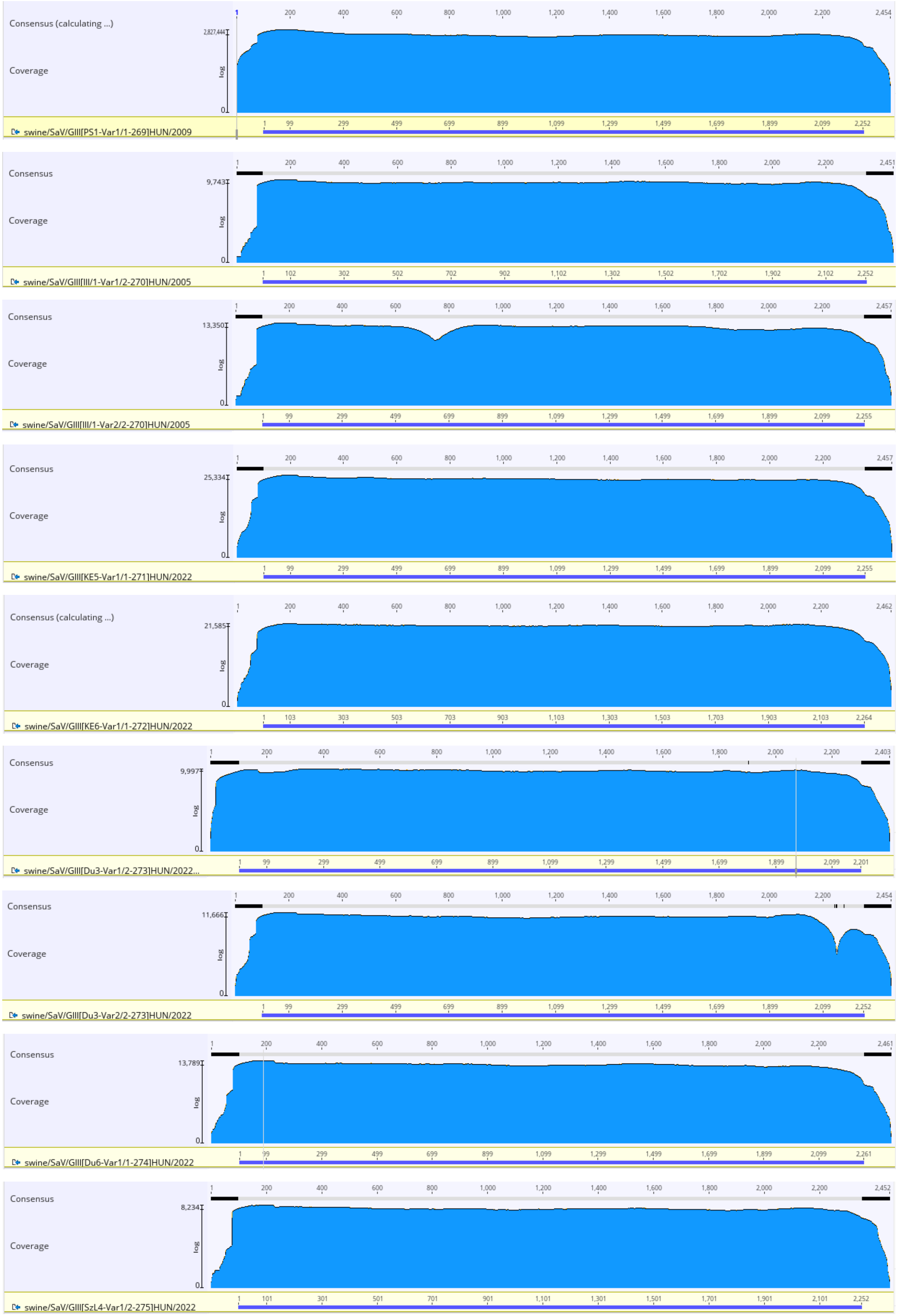

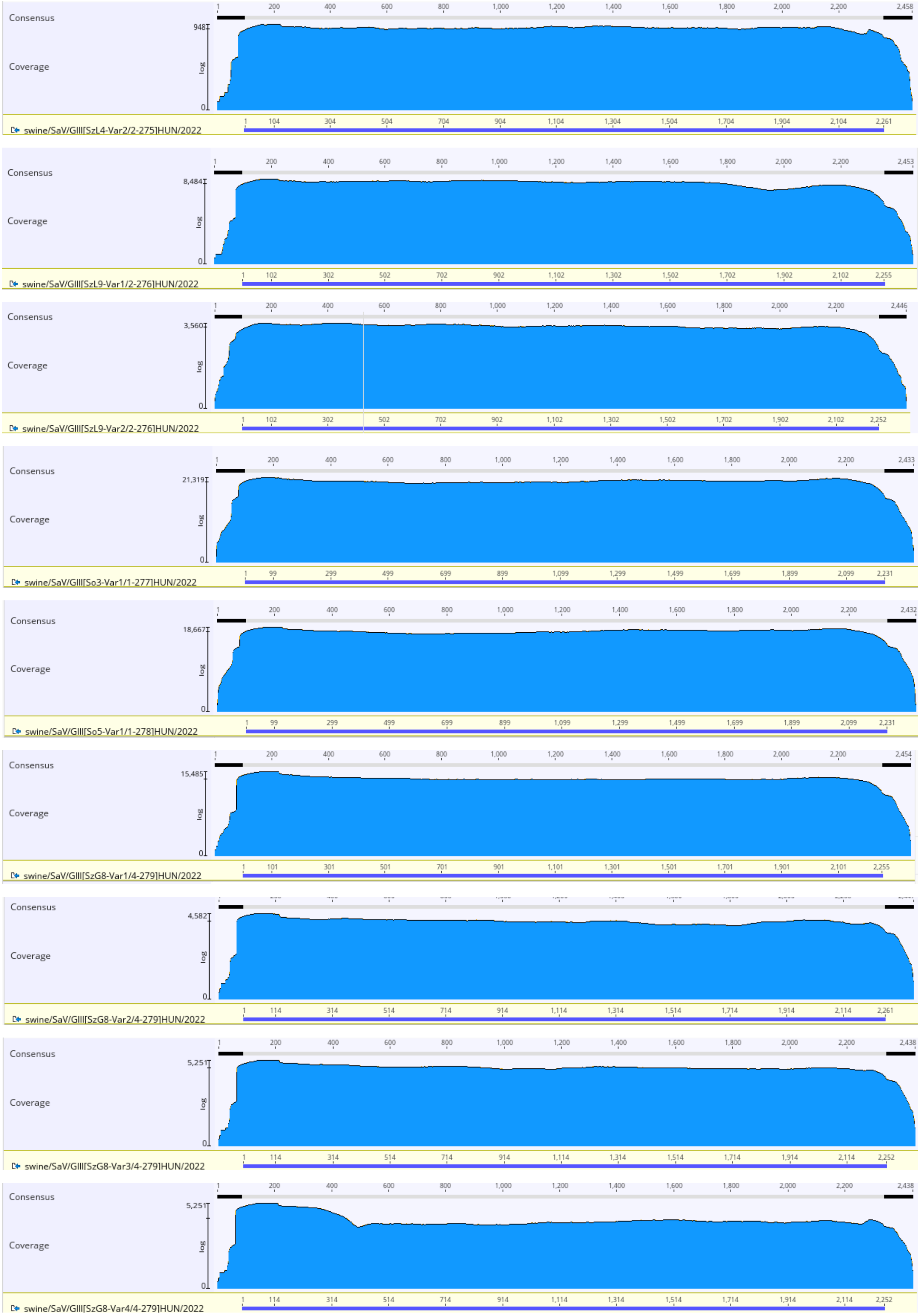
Consensus
Coverage
948
swine/SaV/GIII[SzL4-Var2/2-275]HUN/2022
Consensus
Coverage
8,484
swine/SaV/GIII[SzL9-Var1/2-276]HUN/2022
Consensus
Coverage
3,560
swine/SaV/GIII[SzL9-Var2/2-276]HUN/2022
Consensus
Coverage
21,319
swine/SaV/GIII[So3-Var1/1-277]HUN/2022
Consensus
Coverage
18,667
swine/SaV/GIII[So5-Var1/1-278]HUN/2022
Consensus
Coverage
15,485
swine/SaV/GIII[SzG8-Var1/4-279]HUN/2022
Consensus
Coverage
4,582
swine/SaV/GIII[SzG8-Var2/4-279]HUN/2022
Consensus
Coverage
5,251
swine/SaV/GIII[SzG8-Var3/4-279]HUN/2022
Consensus
Coverage
5,251
swine/SaV/GIII[SzG8-Var4/4-279]HUN/2022

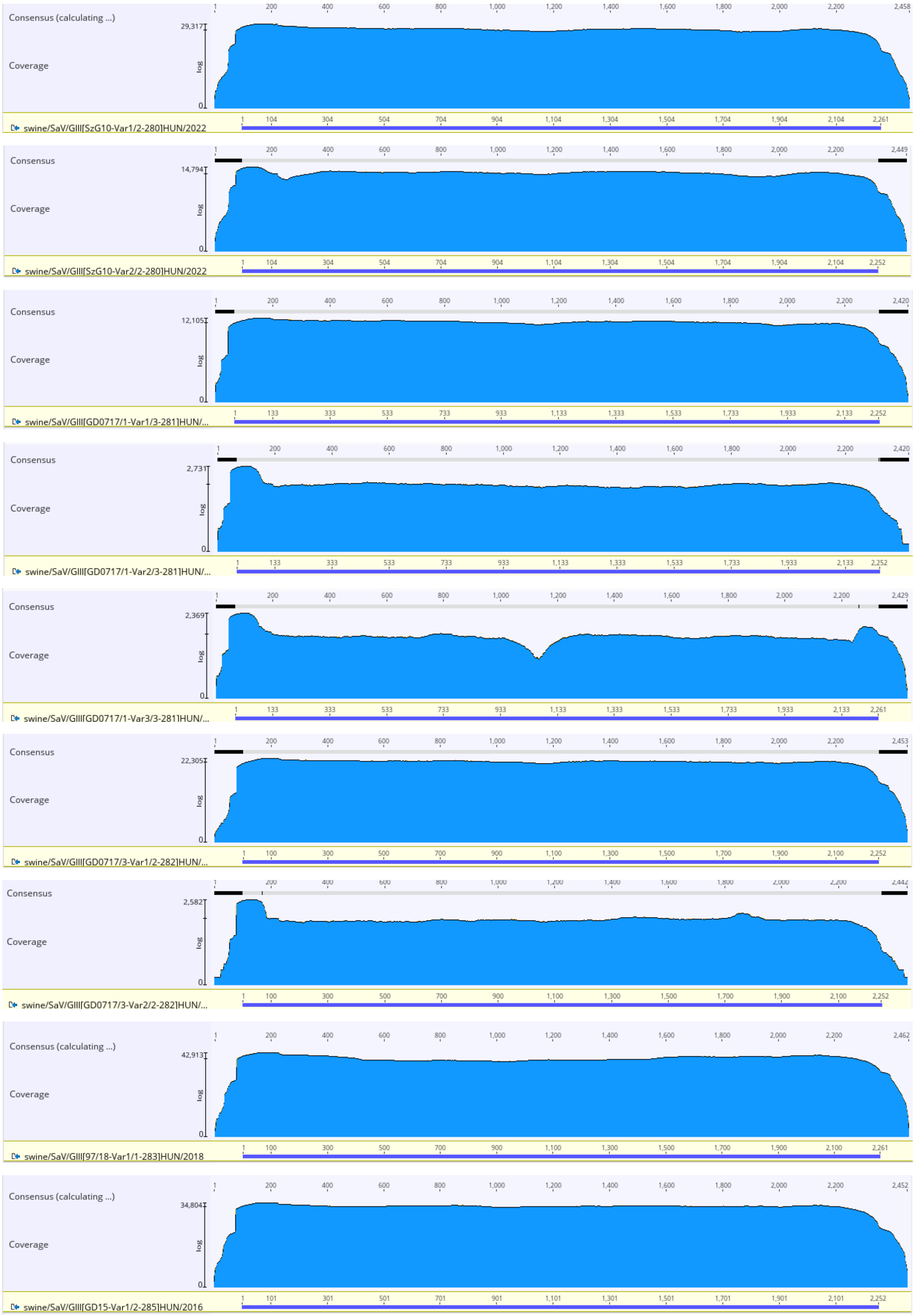

Consensus (calculating ...)
29,317
Coverage
log
swine/SaV/GIII[SzG10-Var1/2-280]HUN/2022
Consensus
14,794
Coverage
log
swine/SaV/GIII[SzG10-Var2/2-280]HUN/2022
Consensus
12,105
Coverage
log
swine/SaV/GIII[GD0717/1-Var1/3-281]HUN/...
Consensus
2,731
Coverage
log
swine/SaV/GIII[GD0717/1-Var2/3-281]HUN/...
Consensus
2,369
Coverage
log
swine/SaV/GIII[GD0717/1-Var3/3-281]HUN/...
Consensus
22,305
Coverage
log
swine/SaV/GIII[GD0717/3-Var1/2-282]HUN/...
Consensus
2,582
Coverage
log
swine/SaV/GIII[GD0717/3-Var2/2-282]HUN/...
Consensus (calculating ...)
42,913
Coverage
log
swine/SaV/GIII[97/18-Var1/1-283]HUN/2018
Consensus (calculating ...)
34,804
Coverage
log
swine/SaV/GIII[GD15-Var1/2-285]HUN/2016

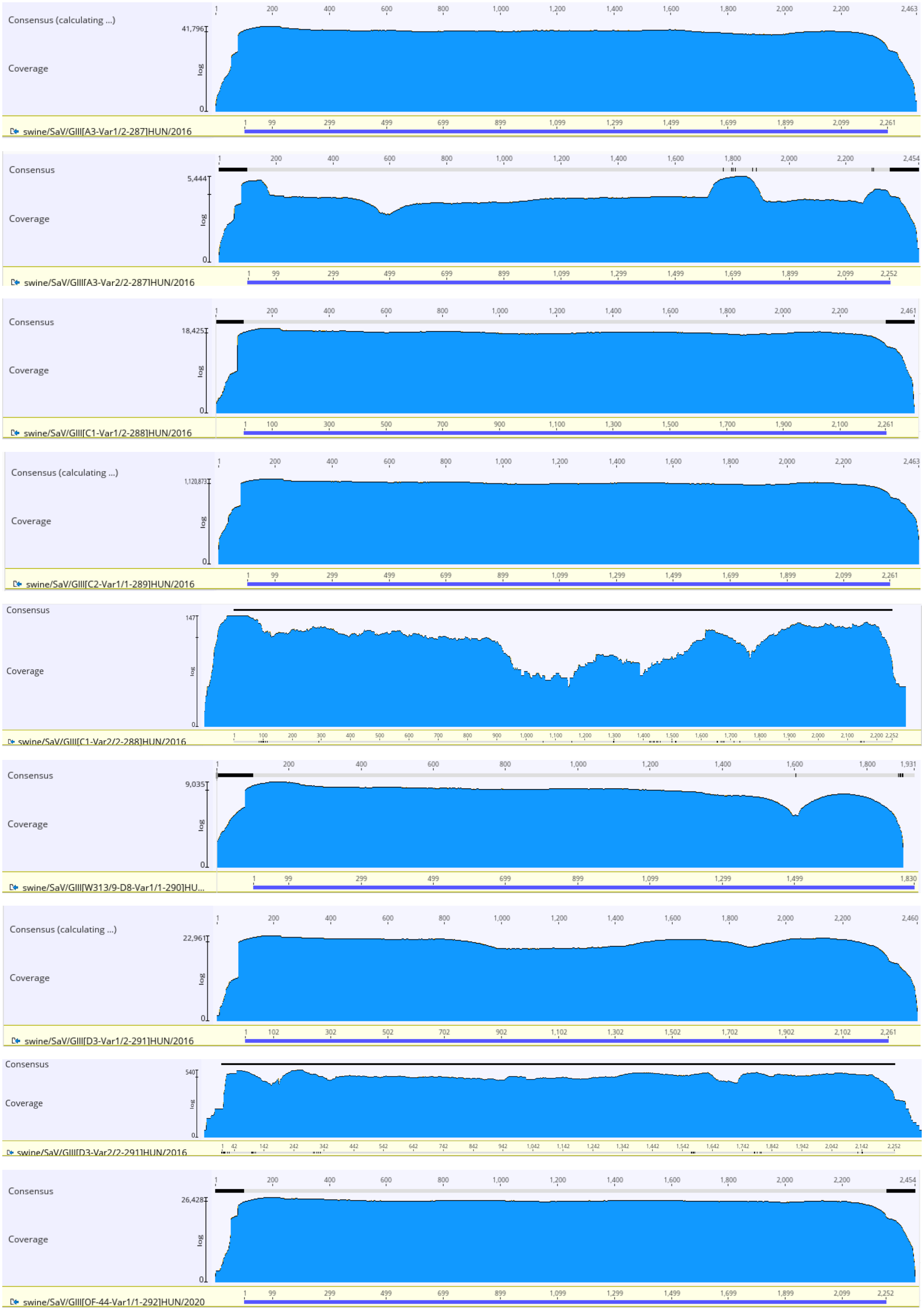

Consensus (calculating ...)
Coverage
41,796
swine/SaV/GIII[A3-Var1/2-287]HUN/2016
Consensus
Coverage
5,444
swine/SaV/GIII[A3-Var2/2-287]HUN/2016
Consensus
Coverage
18,425
swine/SaV/GIII[C1-Var1/2-288]HUN/2016
Consensus (calculating ...)
Coverage
swine/SaV/GIII[C2-Var1/1-289]HUN/2016
Consensus
Coverage
147
swine/SaV/GIII[C1-Var2/2-288]HUN/2016
Consensus
Coverage
9,035
swine/SaV/GIII[W313/9-D8-Var1/1-290]HU...
Consensus (calculating ...)
Coverage
22,961
swine/SaV/GIII[D3-Var1/2-291]HUN/2016
Consensus
Coverage
540
swine/SaV/GIII[D3-Var2/2-291]HUN/2016
Consensus
Coverage
26,428
swine/SaV/GIII[OF-44-Var1/1-292]HUN/2020

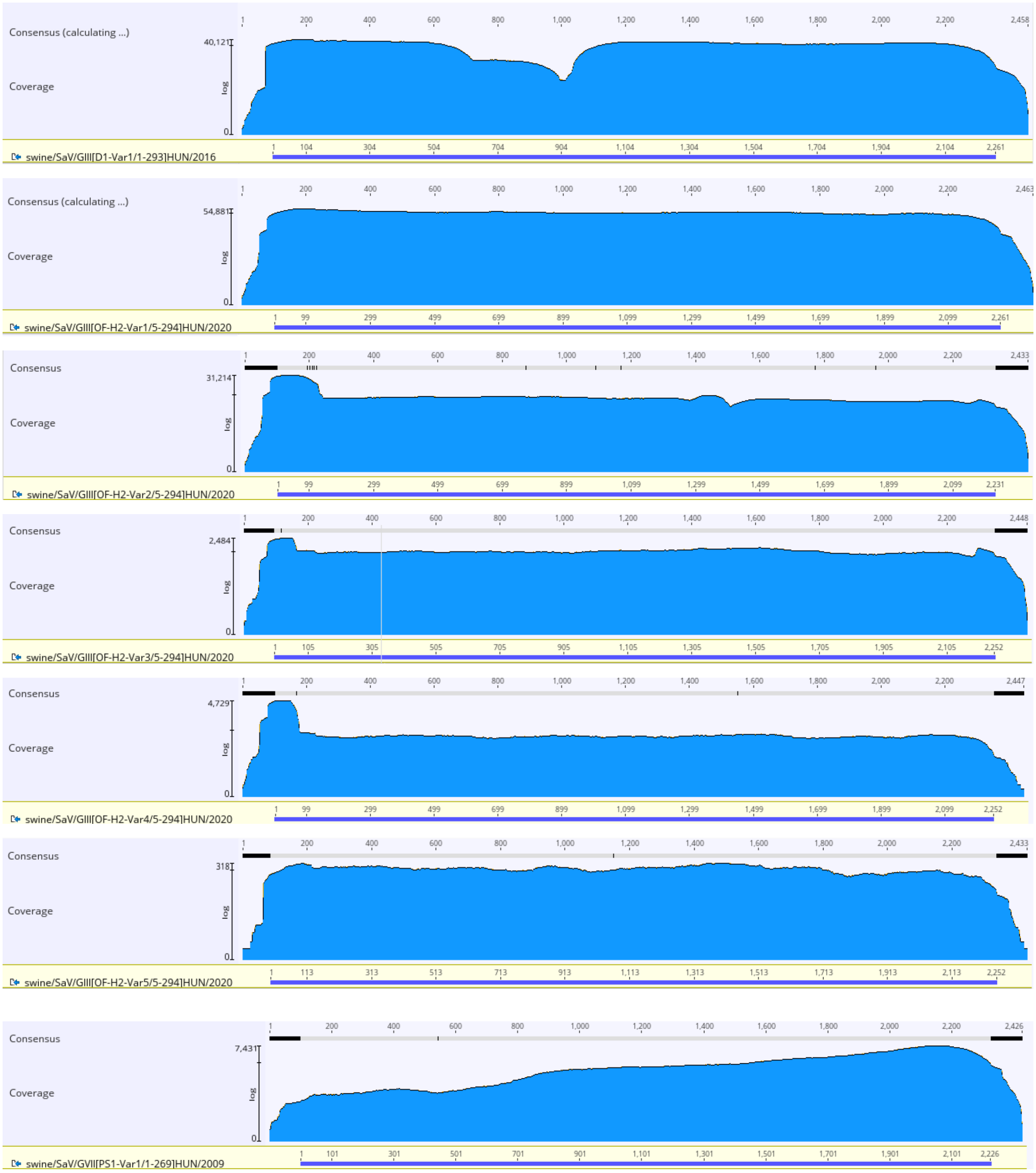
Consensus (calculating ...)
Coverage
40,121
log
0
swine/SaV/GIII[D1-Var1/1-293]HUN/2016
Consensus (calculating ...)
Coverage
54,881
log
0
swine/SaV/GIII[OF-H2-Var1/5-294]HUN/2020
Consensus
Coverage
31,214
log
0
swine/SaV/GIII[OF-H2-Var2/5-294]HUN/2020
Consensus
Coverage
2,484
log
0
swine/SaV/GIII[OF-H2-Var3/5-294]HUN/2020
Consensus
Coverage
4,729
log
0
swine/SaV/GIII[OF-H2-Var4/5-294]HUN/2020
Consensus
Coverage
318
log
0
swine/SaV/GIII[OF-H2-Var5/5-294]HUN/2020
Consensus
Coverage
7,431
log
0
swine/SaV/GVII[PS1-Var1/1-269]HUN/2009

∑=45

(S3-B) Sw-NoV consensus coverage maps:

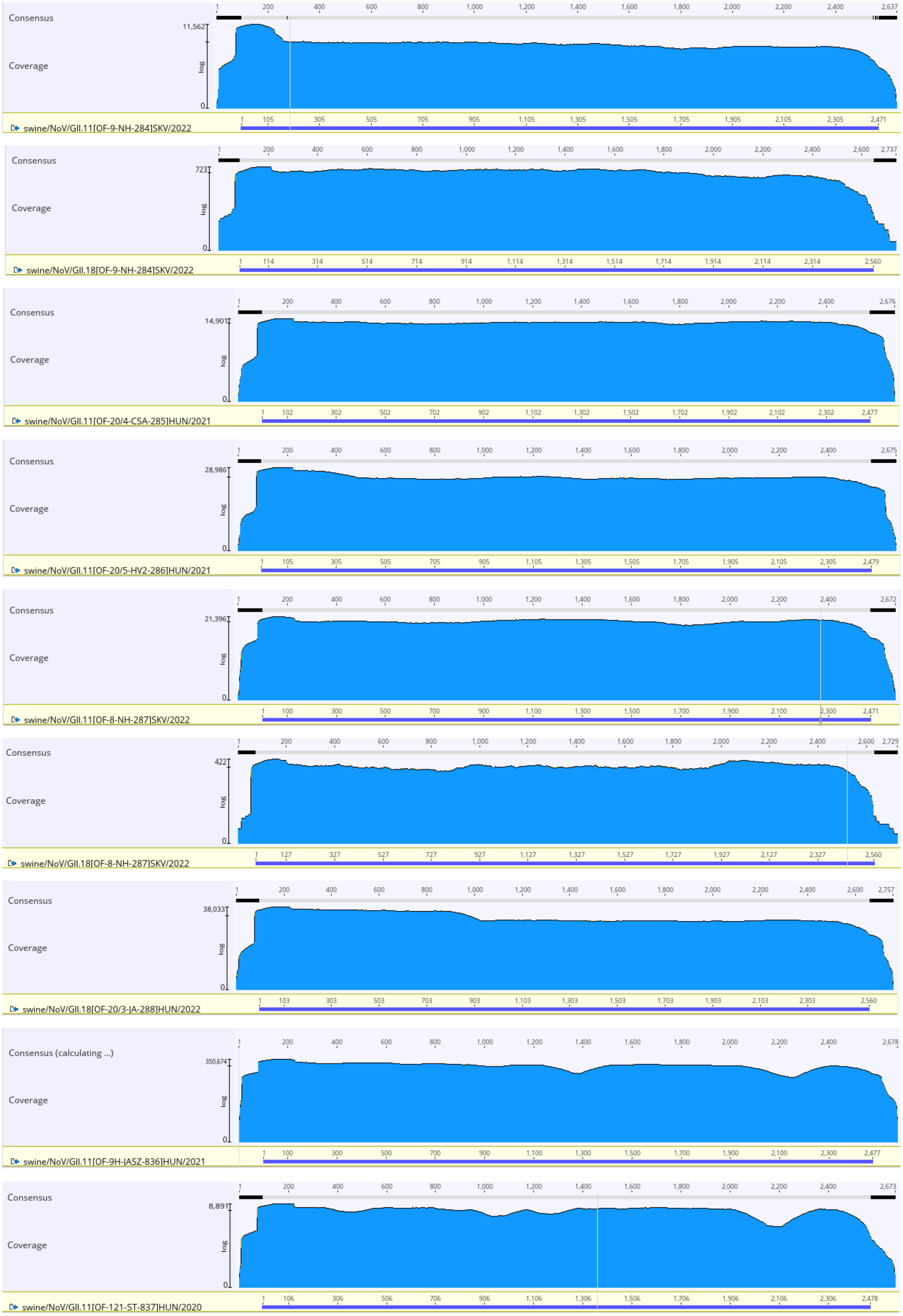

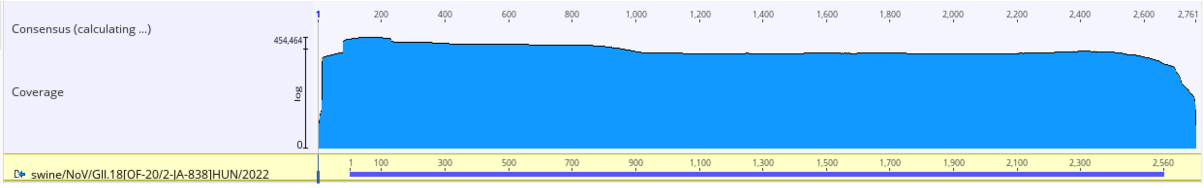
Consensus (calculating ...)
Coverage
454,464
log
swine/NoV/GII.18[OF-20/2-JA-838]HUN/2022

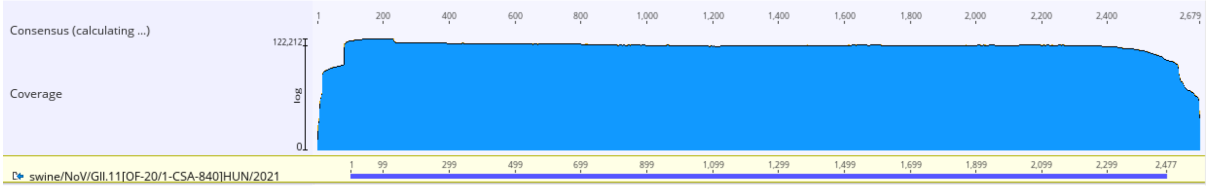
Consensus (calculating ...)
Coverage
122,212
log
swine/NoV/GII.11[OF-20/1-CSA-840]HUN/2021

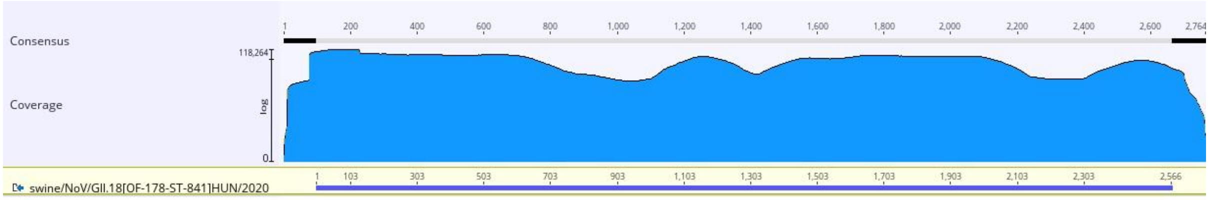
Consensus
Coverage
118,264
log
swine/NoV/GII.18[OF-178-ST-841]HUN/2020

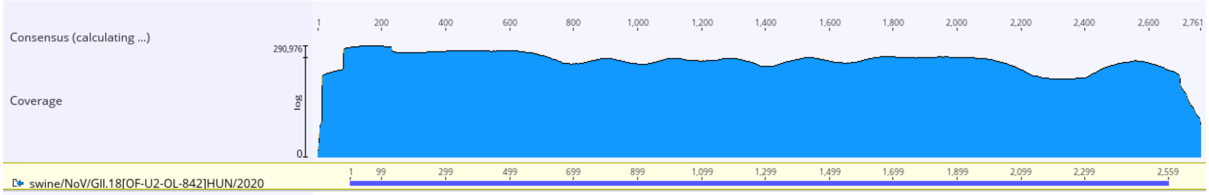
Consensus (calculating ...)
Coverage
290,976
log
swine/NoV/GII.18[OF-U2-OL-842]HUN/2020

∑=13

(S3-C) Sw-VaV consensus coverage maps:

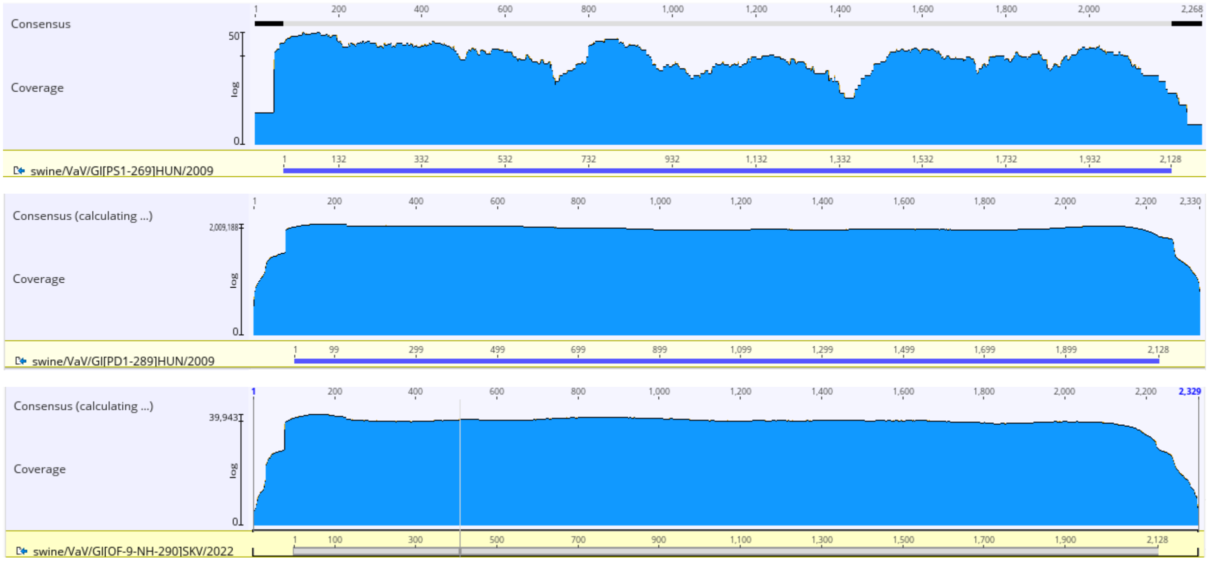

∑=3

Figure S3: Coverage maps of swine sapovirus (Sw-SaV, Panel A), swine norovirus (Sw-NoV, panel B) and swine valovirus (Sw-VaV, panel C) consensus sequences generated by Geneious Mapper of Geneious Prime ver. 2024.0.7 with the following settings: minimum overlap between reads: 50 nt, minimum overlap identity: 97%, maximum mismatch per read: 3%, max ambiguity: 8, the source files were the quality checked and adapter trimmed fastq files (*.QCT_fastq, see Figure 5).

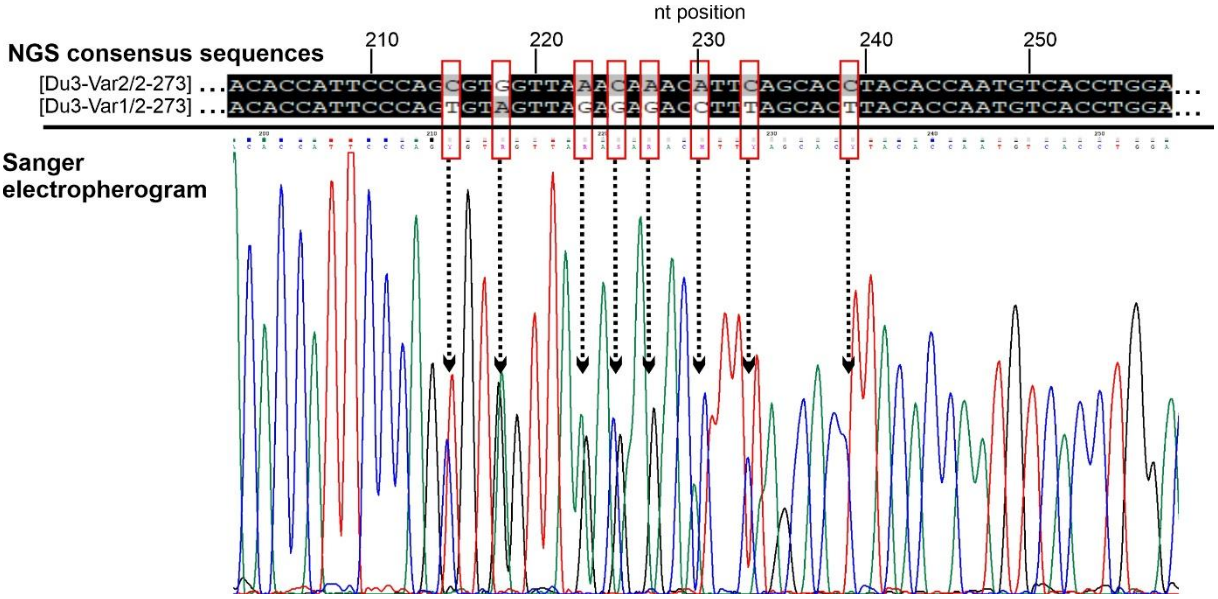

Figure S4: Comparison of sapovirus consensus sequence variants (Var1/2, PQ483461 and Var2/2, PQ483462) of sample Du3 assembled from the NGS sequencing data (top part) and the Sanger electropherogram (bottom) of the same PCR product. Positions of mixed bases are highlighted with red boxes in the consensus sequences and arrows in the electropherogram.

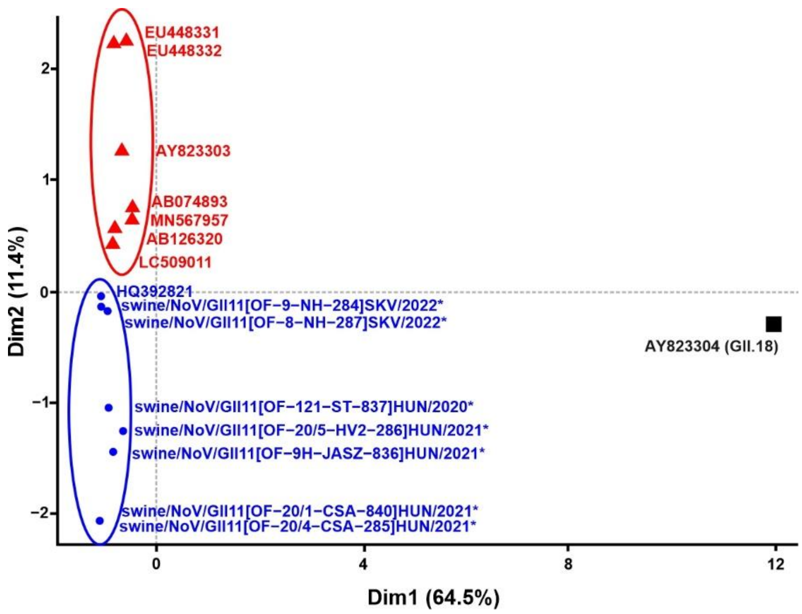

Figure S5: Cluster analysis of complete VP1 nucleotide sequences of swine norovirus GII.11. The analysis contained n=15 sequences including all the study GII.11 strains (marked with an asterisk) and a GII.18 strain (AY823304) which was used as an outgroup. Sequences clustered together by the analysis have been circled and marked with the same colour (red and blue). Note that sequences from the VP1 phylogenetic sub-clades (sc-1 and sc-2) of GII.11 (Figure 6) were also separated into two clusters in this analysis.

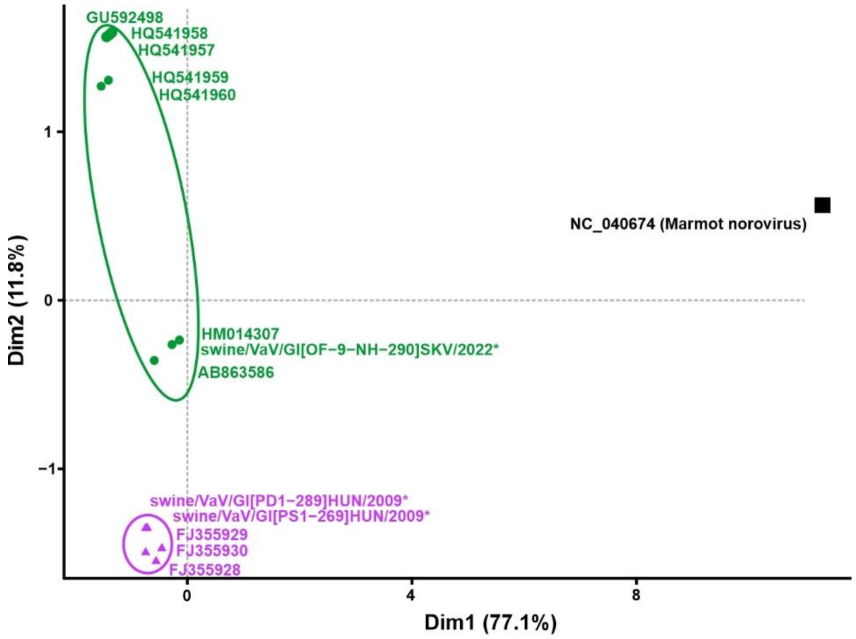

Figure S6: Cluster analysis of complete VP1 nucleotide sequences of all available swine valoviruses (including the study strains marked with asterisks) and a marmot norovirus (NC_040674) which was used as an outgroup. The analysis included n=13. Sequences clustered together by the analysis have been circled and marked with the same colour (green and purple). Note that sequences from the VP1 phylogenetic sub-clades (sc-1 and sc-2) of swine valoviruses (Figure 7) were also separated into two clusters in this analysis.

Target virus	Acc. Number	Strain name	Genogroup.type	Position	Length (bp)
Sapovirus	LC215875	Ishi-Im1-3	GIII	4630-5109	512
Norovirus	AB074893	Sw/NLV/Sw918/1997/JP	GII.11	406-886	512
Valovirus	HM014307	25A/IT/09	GI	208-687	512

Table S1: Features of genome regions of selected sapo-, noro-, and valovirus reference strains used for the production of RNA standards. Acc. Number: GenBank accession number.

Farm ID	Sample ID	Collection date	Age of the host	Health status	SW-SaV qPCR (Cq)	Sw-NoV qPCR (Cq)	Sw-VaV qPCR (Cq)	Sw-SaV 3'RACE snPCR	Sw-NoV 3'RACE snPCR	Sw-VaV 3'RACE snPCR	3RACE-snPCR product ID
Ravazd	120/18 *	18/07/2018	≈21–25 days	DR	-	-	-	n.p.	n.p.	n.p.	-
	97/18*	18/07/2018	≈21–25 days	DR	27.74	-	-	+#	n.p.	n.p.	**97/18-SaV-3R-PCR2**
Nagyszokoly	NSzK1*	01/23/ 2013	≈ 11-12 mo.	Non-DR	-	-	-	n.p.	n.p.	n.p.	-
Zsana	ZSANA 1*	04/14/ 2013	≈70 days	Non-DR	-	32.45	-	n.p.	+#	n.p.	**Zsana1-NoV-3R-PCR2**
	ZSANA 2*	04/14/ 2013	≈70 days	Non-DR	37.01	32.71	-	-	n.p.	n.p.	-
	ZSANA 3*	04/14/ 2013	≈70 days	Non-DR	-	-	-	n.p.	n.p.	n.p.	-
Kevermes	KEV-1*	09/05/ 2013	≈ 4.5 mo.	Non-DR	-	-	-	n.p.	n.p.	n.p.	-
	KEV-2*	09/05/ 2013	≈ 4.5 mo.	Non-DR	-	-	-	n.p.	n.p.	n.p.	-
	KEV-3*	09/05/ 2013	≈ 4.5 mo.	Non-DR	-	-	-	n.p.	n.p.	n.p.	-
Bőny	AM 3	01/13/2017	≈10 days	DR	-	-	-	n.p.	n.p.	n.p.	-
	AM 4	01/13/2017	≈10 days	DR	-	-	-	n.p.	n.p.	n.p.	-
	AM 5	01/13/2017	≈10 days	DR	-	-	-	n.p.	n.p.	n.p.	-
	AM 7	01/13/2017	≈10 days	DR	-	-	-	n.p.	n.p.	n.p.	-
	AM 8	01/13/2017	≈10 days	DR	-	-	-	n.p.	n.p.	n.p.	-
Egyházasfalu	EF1	08/17/ 2016	≈ 3 mo.	Non-DR	-	-	-	n.p.	n.p.	n.p.	-
	EF2	08/17/ 2016	≈ 3 mo.	Non-DR	-	-	-	n.p.	n.p.	n.p.	-
	EF3	08/17/ 2016	≈ 3 mo.	Non-DR	-	-	-	n.p.	n.p.	n.p.	-
	EF4	08/17/ 2016	≈ 3 mo.	Non-DR	-	-	-	n.p.	n.p.	n.p.	-
	EF5	08/17/ 2016	≈ 3 mo.	Non-DR	-	-	-	n.p.	n.p.	n.p.	-
	EF6	08/17/ 2016	≈ 3 mo.	Non-DR	-	-	-	n.p.	n.p.	n.p.	-
	EF7	08/17/ 2016	≈ 3 mo.	Non-DR	-	-	-	n.p.	n.p.	n.p.	-
	EF8	08/17/ 2016	≈ 3 mo.	Non-DR	-	-	-	n.p.	n.p.	n.p.	-
	EF9	08/17/ 2016	≈ 3 mo.	Non-DR	-	-	-	n.p.	n.p.	n.p.	-
	EF10	08/17/ 2016	≈ 3 mo.	Non-DR	-	-	-	n.p.	n.p.	n.p.	-
	EF11	08/17/ 2016	≈ 3 mo.	Non-DR	-	-	-	n.p.	n.p.	n.p.	-
	EF12	08/17/ 2016	≈ 3 mo.	Non-DR	-	-	-	n.p.	n.p.	n.p.	-
	EF13	08/17/ 2016	≈ 3 mo.	Non-DR	-	-	-	n.p.	n.p.	n.p.	-
	EF14	08/17/ 2016	≈ 3 mo.	Non-DR	-	-	-	n.p.	n.p.	n.p.	-

	EF15	08/17/ 2016	≈ 3 mo.	Non-DR	-	-	-	n.p.	n.p.	n.p.	-
	EF16	08/17/ 2016	≈ 3 mo.	Non-DR	-	-	-	n.p.	n.p.	n.p.	-
	EF17	08/17/ 2016	≈ 3 mo.	Non-DR	-	-	-	n.p.	n.p.	n.p.	-
	EF18	08/17/ 2016	≈ 3 mo.	Non-DR	-	-	-	n.p.	n.p.	n.p.	-
	EF19	08/17/ 2016	≈ 3 mo.	Non-DR	-	-	-	n.p.	n.p.	n.p.	-
	EF20	08/17/ 2016	≈ 3 mo.	Non-DR	-	-	-	n.p.	n.p.	n.p.	-
Szentkútpuszta	I/1*	20/07/2005	≈ 3 mo.	Non-DR	-	-	-	n.p.	n.p.	n.p.	-
	I/2*	20/07/2005	≈ 3 mo.	Non-DR	-	-	-	n.p.	n.p.	n.p.	-
	II/1*	20/07/2005	≈ 3 mo.	Non-DR	-	-	-	n.p.	n.p.	n.p.	-
	II/2*	20/07/2005	≈ 3 mo.	Non-DR	-	-	-	n.p.	n.p.	n.p.	-
	II/3*	20/07/2005	≈ 3 mo.	Non-DR	-	-	-	n.p.	n.p.	n.p.	-
	II/10*	20/07/2005	≈ 3 mo.	Non-DR	-	-	-	n.p.	n.p.	n.p.	-
	III/1*	20/07/2005	≈ 3 mo.	Non-DR	33.75	-	-	+#	n.p.	n.p.	**III/1-SaV-3R-PCR2**
	IV/1*	20/07/2005	≈ 3 mo.	Non-DR	-	-	-	n.p.	n.p.	n.p.	-
	V/1*	20/07/2005	≈ 3 mo.	Non-DR	-	-	-	n.p.	n.p.	n.p.	-
	V/2*	20/07/2005	≈ 3 mo.	Non-DR	-	-	-	n.p.	n.p.	n.p.	-
Bőszénfa	WB 2A*	22/04/2011	≈40 days	Non-DR	-	-	-	n.p.	n.p.	n.p.	-
	WB 2B*	22/04/2011	≈40 days	Non-DR	-	-	-	n.p.	n.p.	n.p.	-
	WB 2C*	22/04/2011	≈40 days	Non-DR	-	-	-	n.p.	n.p.	n.p.	-
	WB 2D*	22/04/2011	≈40 days	Non-DR	-	-	-	n.p.	n.p.	n.p.	-
	WB 2E*	22/04/2011	≈40 days	Non-DR	-	-	-	n.p.	n.p.	n.p.	-
Tóthfalu	WB TF1*	17/05/2011	≈ 2 mo.	Non-DR	-	-	-	n.p.	n.p.	n.p.	-
	WB TF2*	17/05/2011	≈ 2 mo.	Non-DR	-	-	-	n.p.	n.p.	n.p.	-
	WB TF3*	17/05/2011	≈ 2 mo.	Non-DR	-	35.31	-	n.p.	-	n.p.	-
	WB TF4*	17/05/2011	≈ 2 mo.	Non-DR	-	-	-	n.p.	n.p.	n.p.	-
	WB TF5*	17/05/2011	≈ 2 mo.	Non-DR	-	-	-	n.p.	n.p.	n.p.	-
	WB TF6*	17/05/2011	≈ 2 mo.	Non-DR	-	-	-	n.p.	n.p.	n.p.	-
	WB TF7*	17/05/2011	≈ 2 mo.	Non-DR	-	35.78	-	n.p.	-	n.p.	-
	WB TF8*	17/05/2011	≈ 2 mo.	Non-DR	-	-	-	n.p.	n.p.	n.p.	-
	WB TF9*	17/05/2011	≈ 2 mo.	Non-DR	-	-	-	n.p.	n.p.	n.p.	-
	WB TF10*	17/05/2011	≈ 2 mo.	Non-DR	-	-	-	n.p.	n.p.	n.p.	-
	WB TF11*	17/05/2011	≈ 2 mo.	Non-DR	-	-	-	n.p.	n.p.	n.p.	-
Orosháza	GD0717/1	17/07/2020	≈21–25 days	Non-DR	32.27	-	-	+#	n.p.	n.p.	**GD0717/1-SaV-3R-PCR2**

	GD0717/2	17/07/2020	≈21–25 days	Non-DR	33.17	-	-	n.p.	n.p.	n.p.	**-**
	GD0717/3	17/07/2020	≈21–25 days	Non-DR	34.07	-	-	+#	n.p.	n.p.	**GD0717/3-SaV-3R-PCR2**
	GD11	09/15/2016	≈21–25 days	Non-DR	-	-	-	n.p.	n.p.	n.p.	-
	GD12	09/15/2016	≈21–25 days	Non-DR	27.13	-	-	+#	n.p.	n.p.	**GD12-SaV-3R-PCR2**
	GD13	09/15/2016	≈21–25 days	Non-DR	-	-	-	n.p.	n.p.	n.p.	-
	GD14	09/15/2016	≈21–25 days	Non-DR	36.36	-	-	n.p.	n.p.	n.p.	**-**
	GD15	09/15/2016	≈21–25 days	Non-DR	32.14	-	-	+#	n.p.	n.p.	**GD15-SaV-3R-PCR2**
	GD16	09/15/2016	≈21–25 days	Non-DR	-	-	-	n.p.	n.p.	n.p.	-
	GD17	09/15/2016	≈21–25 days	Non-DR	-	-	-	n.p.	n.p.	n.p.	-
	GD18	09/15/2016	≈21–25 days	Non-DR	-	-	-	n.p.	n.p.	n.p.	-
	GD19	09/15/2016	≈21–25 days	Non-DR	-	-	-	n.p.	n.p.	n.p.	-
Balmazújváros	BUV-1*	04/24/ 2013	21-35 days	Non-DR	25.15	-	-	+	n.p.	n.p.	-
	BUV-2*	04/24/ 2013	21-35 days	Non-DR	25.59	-	-	+	n.p.	n.p.	-
Városföld	A1	08/12/ 2016	≈40 days	Non-DR	-	-	-	n.p.	n.p.	n.p.	-
	A2	08/12/ 2016	≈40 days	Non-DR	28.71	-	-	+#	n.p.	n.p.	**A2-SaV-3R-PCR2**
	A3	08/12/ 2016	≈40 days	Non-DR	23.59	-	-	+#	n.p.	n.p.	**A3-SaV-3R-PCR2**
	A4	08/12/ 2016	≈40 days	Non-DR	-	-	-	n.p.	n.p.	n.p.	-
	A5	08/12/ 2016	≈40 days	Non-DR	-	-	-	n.p.	n.p.	n.p.	-
Katymár	B1	08/18/ 2016	30-35 days	Non-DR	-	-	-	n.p.	n.p.	n.p.	-
	B2	08/18/ 2016	30-35 days	Non-DR	32.56	-	-	-	n.p.	n.p.	-
	B3	08/18/ 2016	30-35 days	Non-DR	-	-	-	n.p.	n.p.	n.p.	-
	B4	08/18/ 2016	30-35 days	Non-DR	-	-	-	n.p.	n.p.	n.p.	-
	B5	08/18/ 2016	30-35 days	Non-DR	-	-	-	n.p.	n.p.	n.p.	-
Bácsalmás	C1	08/18/ 2016	≈40 days	Non-DR	24.35	-	-	+#	n.p.	n.p.	**C1-SaV-3R-PCR2**
	C2	08/18/ 2016	≈40 days	Non-DR	32.11	-	-	+#	n.p.	n.p.	**C2-SaV-3R-PCR2**
	C3	08/18/ 2016	≈40 days	Non-DR	-	-	-	n.p.	n.p.	n.p.	-
	C4	08/18/ 2016	≈40 days	Non-DR	-	-	-	n.p.	n.p.	n.p.	-
	C5	08/18/ 2016	≈40 days	Non-DR	-	-	-	n.p.	n.p.	n.p.	-
Nyíribrony	D1	08/10/ 2016	30-35 days	Non-DR	33.34	-	-	+#	n.p.	n.p.	**D1-SaV-3R-PCR2**
	D2	08/10/ 2016	30-35 days	Non-DR	-	-	-	n.p.	n.p.	n.p.	-
	D3	08/10/ 2016	30-35 days	Non-DR	34.00	-	-	+#	n.p.	n.p.	**D3-SaV-3R-PCR2**
	D4	08/10/ 2016	30-35 days	Non-DR	34.91	-	-	n.p.	n.p.	n.p.	**-**
	D5	08/10/ 2016	30-35 days	Non-DR	-	-	-	n.p.	n.p.	n.p.	-

Tázlár	TM-1130*	01/2013	7-21 days	DR	-	-	-	n.p.	n.p.	n.p.	-
	TM-1205*	01/2013	7-21 days	DR	-	-	-	n.p.	n.p.	n.p.	-
	TM-1121*	01/2013	7-21 days	DR	14.38	-	-	+	n.p.	n.p.	**TM-1121-SaV-3R-PCR2**
	TKo-1*	01/2013	≈ 11-12 mo.	Non-DR	-	-	-	n.p.	n.p.	n.p.	-
	TKo-2*	01/2013	≈ 11-12 mo.	Non-DR	-	-	-	n.p.	n.p.	n.p.	-
	TKo-3*	01/2013	≈ 11-12 mo.	Non-DR	-	-	-	n.p.	n.p.	n.p.	-
	TKo-4*	01/2013	≈ 11-12 mo.	Non-DR	-	-	-	n.p.	n.p.	n.p.	-
Szigetvár	SzV1018/1	18/10/2017	≈21–25 days	Non-DR	-	-	-	n.p.	n.p.	n.p.	-
	SzV1018/2	18/10/2017	≈21–25 days	Non-DR	-	-	-	n.p.	n.p.	n.p.	-
	SzV1018/3	18/10/2017	≈21–25 days	Non-DR	-	-	-	n.p.	n.p.	n.p.	-
	SzV1018/4	18/10/2017	≈21–25 days	Non-DR	-	-	-	n.p.	n.p.	n.p.	-
	SzV1018/5	18/10/2017	≈21–25 days	Non-DR	-	-	-	n.p.	n.p.	n.p.	-
	SzV1018/6	18/10/2017	≈21–25 days	Non-DR	-	-	-	n.p.	n.p.	n.p.	-
	SzV1018/7	18/10/2017	≈21–25 days	Non-DR	-	-	-	n.p.	n.p.	n.p.	-
	SzV1018/8	18/10/2017	≈21–25 days	Non-DR	-	-	-	n.p.	n.p.	n.p.	-
	SzV1018/9	18/10/2017	≈21–25 days	Non-DR	-	-	-	n.p.	n.p.	n.p.	-
	SzV1018/10	18/10/2017	≈21–25 days	Non-DR	-	-	-	n.p.	n.p.	n.p.	-
	SzV1018/11	18/10/2017	≈21–25 days	Non-DR	-	-	-	n.p.	n.p.	n.p.	-
	SzV1018/12	18/10/2017	≈21–25 days	Non-DR	-	-	-	n.p.	n.p.	n.p.	-
	SzG-1	03/04/2022	21 days	DR	-	-	-	n.p.	n.p.	n.p.	-
	SzG-2	03/04/2022	21 days	DR	34.23	-	-	n.p.	n.p.	n.p.	-
	SzG-3	03/04/2022	21 days	DR	-	-	-	n.p.	n.p.	n.p.	-
	SzG-4	03/04/2022	21 days	DR	-	-	-	n.p.	n.p.	n.p.	-
	SzG-5	03/04/2022	21 days	DR	-	-	-	n.p.	n.p.	n.p.	-
	SzG-6	03/04/2022	21 days	DR	-	-	-	n.p.	n.p.	n.p.	-
	SzG-7	03/04/2022	21 days	DR	-	-	-	n.p.	n.p.	n.p.	-
	SzG-8	03/04/2022	21 days	DR	30.08	-	-	+#	n.p.	n.p.	**SzG8-SaV-3R-PCR2**
	SzG-9	03/04/2022	21 days	DR	-	-	-	n.p.	n.p.	n.p.	-
	SzG-10	03/04/2022	21 days	DR	33.16	-	-	+#	n.p.	n.p.	**SzG10-SaV-3R-PCR2**
Ebes	K1*	01/11/2008	4-weeks	Non-DR	-	-	-	n.p.	n.p.	n.p.	-
	K2*	01/11/2008	4-weeks	Non-DR	-	-	-	n.p.	n.p.	n.p.	-
	K3*	01/11/2008	4-weeks	Non-DR	-	-	-	n.p.	n.p.	n.p.	-
	K4*	01/11/2008	4-weeks	Non-DR	-	-	-	n.p.	n.p.	n.p.	-

	K5*	01/11/2008	4-weeks	Non-DR	-	-	-	n.p.	n.p.	n.p.	-
	K7*	01/11/2008	4-weeks	Non-DR	-	-	-	n.p.	n.p.	n.p.	-
	K21*	01/11/2008	10 days	Non-DR	-	-	-	n.p.	n.p.	n.p.	-
	K22*	01/11/2008	10 days	Non-DR	-	-	-	n.p.	n.p.	n.p.	-
	K30*	01/11/2008	10 days	Non-DR	-	-	-	n.p.	n.p.	n.p.	-
	K25*	01/11/2008	10 days	Non-DR	-	-	-	n.p.	n.p.	n.p.	-
	K29*	01/11/2008	10 days	Non-DR	-	-	-	n.p.	n.p.	n.p.	-
	K31*	01/11/2008	3 mo.	Non-DR	-	-	-	n.p.	n.p.	n.p.	-
	K32*	01/11/2008	3 mo.	Non-DR	-	-	-	n.p.	n.p.	n.p.	-
	K33*	01/11/2008	3 mo.	Non-DR	-	-	-	n.p.	n.p.	n.p.	-
	K34*	01/11/2008	3 mo.	Non-DR	-	-	-	n.p.	n.p.	n.p.	-
	K35*	01/11/2008	3 mo.	Non-DR	-	-	-	n.p.	n.p.	n.p.	-
	K36*	01/11/2008	3 mo.	Non-DR	-	-	-	n.p.	n.p.	n.p.	-
	K46*	01/11/2008	6 mo.	Non-DR	-	-	-	n.p.	n.p.	n.p.	-
	K47*	01/11/2008	6 mo.	Non-DR	-	-	-	n.p.	n.p.	n.p.	-
	K48*	01/11/2008	6 mo.	Non-DR	-	-	-	n.p.	n.p.	n.p.	-
	K49*	01/11/2008	6 mo.	Non-DR	-	-	-	n.p.	n.p.	n.p.	-
	K50*	01/11/2008	6 mo.	Non-DR	-	-	-	n.p.	n.p.	n.p.	-
	K51*	01/11/2008	6 mo.	Non-DR	-	-	-	n.p.	n.p.	n.p.	-
Kecel	KE-1	10/05/2022	21 days	DR	38.49	-	-	n.p.	n.p.	n.p.	-
	KE-2	10/05/2022	21 days	DR	-	-	-	n.p.	n.p.	n.p.	-
	KE-3	10/05/2022	21 days	DR	-	-	-	n.p.	n.p.	n.p.	-
	KE-4	10/05/2022	21 days	DR	-	-	-	n.p.	n.p.	n.p.	-
	KE-5	10/05/2022	21 days	DR	24.71	-	-	+#	n.p.	n.p.	**KE5-SaV-3R-PCR2**
	KE-6	10/05/2022	21 days	DR	26.74	-	-	+#	n.p.	n.p.	**KE6-SaV-3R-PCR2**
	KE-7	10/05/2022	21 days	DR	-	-	-	n.p.	n.p.	n.p.	-
	KE-8	10/05/2022	21 days	DR	38.72	-	-	n.p.	n.p.	n.p.	-
	KE-9	10/05/2022	21 days	DR	37.87	-	-	n.p.	n.p.	n.p.	-
	KE-10	10/05/2022	21 days	DR	-	-	-	n.p.	n.p.	n.p.	-
Dunaszekcső	Du-1	12/04/2022	21 days	DR	29.87	-	-	n.p.	n.p.	n.p.	-
	Du-2	12/04/2022	21 days	DR	37.16	-	-	n.p.	n.p.	n.p.	-
	Du-3	12/04/2022	21 days	DR	19.79	-	-	+#	n.p.	n.p.	**Du3-SaV-3R-PCR2**
	Du-4	12/04/2022	21 days	DR	35.41	-	-	n.p.	n.p.	n.p.	-

	Du-5	12/04/2022	21 days	DR	32.09	-	-	n.p.	n.p.	n.p.	-
	Du-6	12/04/2022	21 days	DR	26.71	-	-	+#	n.p.	n.p.	**Du6-SaV-3R-PCR2**
	Du-7	12/04/2022	21 days	DR	35.06	-	-	n.p.	n.p.	n.p.	-
	Du-8	12/04/2022	21 days	DR	35.18	-	-	n.p.	n.p.	n.p.	-
	Du-9	12/04/2022	21 days	DR	35.01	-	-	n.p.	n.p.	n.p.	-
	Du-10	12/04/2022	21 days	DR	32.47	-	-	n.p.	n.p.	n.p.	-
Szilfás	SzL-1	17/04/2022	21 days	DR	-	-	36.82	n.p.	n.p.	+#	**SzL1-VaV-3R-PCR2**
	SzL-2	17/04/2022	21 days	DR	-	-	-	n.p.	n.p.	n.p.	-
	SzL-3	17/04/2022	21 days	DR	-	-	-	n.p.	n.p.	n.p.	-
	SzL-4	17/04/2022	21 days	DR	33.25	-	-	+#	n.p.	n.p.	**SzL4-SaV-3R-PCR2**
	SzL-5	17/04/2022	21 days	DR	-	-	-	n.p.	n.p.	n.p.	-
	SzL-6	17/04/2022	21 days	DR	38.97	-	-	n.p.	n.p.	n.p.	-
	SzL-7	17/04/2022	21 days	DR	-	-	-	n.p.	n.p.	n.p.	-
	SzL-8	17/04/2022	21 days	DR	-	-	-	n.p.	n.p.	n.p.	-
	SzL-9	17/04/2022	21 days	DR	33.97	-	-	+#	n.p.	n.p.	**SzL9-SaV-3R-PCR2**
	SzL-10	17/04/2022	21 days	DR	-	-	-	n.p.	n.p.	n.p.	-
Somberek	So-1	12/04/2022	21 days	DR	32.20	-	-	n.p.	n.p.	n.p.	-
	So-2	12/04/2022	21 days	DR	24.89	-	-	n.p.	n.p.	n.p.	-
	So-3	12/04/2022	21 days	DR	21.39	-	-	+#	n.p.	n.p.	**So3-SaV-3R-PCR2**
	So-4	12/04/2022	21 days	DR	33.38	-	-	n.p.	n.p.	n.p.	-
	So-5	12/04/2022	21 days	DR	21.53	36.48	-	+#	-	n.p.	**So5-SaV-3R-PCR2**
	So-6	12/04/2022	21 days	DR	30.46	-	-	n.p.	n.p.	n.p.	-
	So-7	12/04/2022	21 days	DR	-	-	-	n.p.	n.p.	n.p.	-
	So-8	12/04/2022	21 days	DR	23.51	-	-	n.p.	n.p.	n.p.	-
	So-9	12/04/2022	21 days	DR	29.65	-	-	n.p.	n.p.	n.p.	-
	So-10	12/04/2022	21 days	DR	29.58	-	-	n.p.	n.p.	n.p.	-
Újmajor	UMS-1*	01/04/2010	21 days	DR	-	-	-	n.p.	n.p.	n.p.	-
	UMS-2*	01/04/2010	21 days	DR	-	-	-	n.p.	n.p.	n.p.	-
Pusztaföldvár	PD-1*	27/06/2009	≈70 days	Non-DR	-	-	30.05	n.p.	n.p.	+#	**PD1-VaV-3R-PCR2**
	PS-1*	27/06/2009	21 days	DR	15.12	-	37.40	+#	n.p.	+#	**VaV-3R-PCR2**
Székelyszabar	SZ1M*	01/2013	≈ 11-12 mo.	Non-DR	34.68	-	-	n.p.	n.p.	n.p.	-
	SZ2M*	01/2013	≈ 11-12 mo.	Non-DR	-	-	-	n.p.	n.p.	n.p.	-
	SZ3M*	01/2013	≈ 11-12 mo.	Non-DR	-	-	-	n.p.	n.p.	n.p.	-

SZ4M*	01/2013	≈ 11-12 mo.	Non-DR	-	-	-	n.p.	n.p.	n.p.	-
SZ1K*	01/2013	7-21 days	Non-DR	-	-	-	n.p.	n.p.	n.p.	-
SZ2K*	01/2013	7-21 days	Non-DR	-	-	-	n.p.	n.p.	n.p.	-
SZ3K*	01/2013	7-21 days	Non-DR	-	-	-	n.p.	n.p.	n.p.	-
SZ4K*	01/2013	7-21 days	Non-DR	-	-	-	n.p.	n.p.	n.p.	-

Table S2: Features of analyzed enteric samples and **s**ummaries of the results of triplex swine sapovirus(Sw-SaV)/Norovirus (Sw-NoV)/valovirus (Sw-VaV) RT-qPCR assays with measured Cq values as well as the results of 3'RACE semi-nested PCR (3'RACE-snPCR) reactions of selected qPCR positive samples. ID: identification, DR: diarreic animal, Non-DR: non-diarrheic animal. n.p.: not performed. #: 3'RACE-snPCR products were selected for NGS sequencing. In the 3RACE-snPCR product ID column the identification names of those of the 3'RACE-snPCR products were found which were selected for NGS sequencing. Sample IDs marked with asterisk (*) indicate faecal samples while all other, unmarked samples are individual rectal swabs.

Farm ID	Sample ID	Collection date	Age	Health status	SW-SaV qPCR (Cq)	Sw-NoV qPCR (Cq)	Sw-VaV qPCR (Cq)	Sw-SaV 3'RACE snPCR	Sw-NoV 3'RACE snPCR	Sw-VaV 3'RACE snPCR	3RACE-snPCR product ID
Jászárokszállás (JASZ)	1B-NA JÁSz	2021.01.11-27	10 weeks	AS	-	-	-	n.p.	n.p.	n.p.	-
	2B-NA JÁSz	2021.01.11-27	10 weeks	AS	-	-	-	n.p.	n.p.	n.p.	-
	3B-NA JÁSz	2021.01.11-27	10 weeks	AS	-	-	-	n.p.	n.p.	n.p.	-
	4B-NA JÁSz	2021.01.11-27	10 weeks	AS	-	-	-	n.p.	n.p.	n.p.	-
	5B-NA JÁSz	2021.01.11-27	10 weeks	AS	-	-	-	n.p.	n.p.	n.p.	-
	6H-NA JÁSz	2021.01.11-27	20 weeks	AS	-	-	-	n.p.	n.p.	n.p.	-
	7H-NA JÁSz	2021.01.11-27	20 weeks	AS	-	-	-	n.p.	n.p.	n.p.	-
	8H-NA JÁSz	2021.01.11-27	20 weeks	AS	-	-	-	n.p.	n.p.	n.p.	-
	9H-NA JÁSz	2021.01.11-27	20 weeks	AS	-	36.07	-	n.p.	+#	n.p.	**OF-9H-JASZ-NoV-3R-PCR2**
Somogytarnóca (ST)	37	2020.12.09	10 weeks	AS	-	-	-	n.p.	n.p.	n.p.	-
	44	2020.12.09	10 weeks	AS	-	-	-	n.p.	n.p.	n.p.	-
	51	2020.12.09	10 weeks	AS	-	-	-	n.p.	n.p.	n.p.	-
	58	2020.12.09	10 weeks	AS	-	-	-	n.p.	n.p.	n.p.	-
	65	2020.12.09	10 weeks	AS	-	-	-	n.p.	n.p.	n.p.	-
	79	2020.12.09	20 weeks	AS	-	-	-	n.p.	n.p.	n.p.	-
	86	2020.12.09	20 weeks	AS	-	-	-	n.p.	n.p.	n.p.	-
	121	2020.12.09	20 weeks	AS	-	38.11	-	n.p.	+#	n.p.	**OF-121-ST-NoV-3R-PCR2**
	149	2020.12.09	20 weeks	AS	-	-	-	n.p.	n.p.	n.p.	-
	178	2020.12.09	20 weeks	AS	-	38.01	-	n.p.	+#	n.p.	**OF-178-ST-NoV-3R-PCR2**
Bácsalmás	B1	2022.01.12	10 weeks	AS	-	-	-	n.p.	n.p.	n.p.	-
	B2	2022.01.12	10 weeks	AS	-	-	-	n.p.	n.p.	n.p.	-
	B3	2022.01.12	10 weeks	AS	-	-	-	n.p.	n.p.	n.p.	-
	B4	2022.01.12	10 weeks	AS	-	-	-	n.p.	n.p.	n.p.	-
	B5	2022.01.12	10 weeks	AS	-	-	-	n.p.	n.p.	n.p.	-
	H1	2022.01.12	20 weeks	AS	-	-	-	n.p.	n.p.	n.p.	-
	H2	2022.01.12	20 weeks	AS	-	-	-	n.p.	n.p.	n.p.	-
	H3	2022.01.12	20 weeks	AS	-	-	-	n.p.	n.p.	n.p.	-
	H4	2022.01.12	20 weeks	AS	-	-	-	n.p.	n.p.	n.p.	-
	H5	2022.01.12	20 weeks	AS	-	-	-	n.p.	n.p.	n.p.	-
Fehérgyarmat	1	2021.01.11-12.	10 weeks	AS	-	-	-	n.p.	n.p.	n.p.	-

	2	2021.01.11-12.	10 weeks	AS	-	-	-	n.p.	n.p.	n.p.	-
	3	2021.01.11-12.	10 weeks	AS	-	-	-	n.p.	n.p.	n.p.	-
	4	2021.01.11-12.	10 weeks	AS	-	-	-	n.p.	n.p.	n.p.	-
	5	2021.01.11-12.	10 weeks	AS	-	-	-	n.p.	n.p.	n.p.	-
	6	2021.01.11-12.	20 weeks	AS	-	36.05	-	n.p.	n.p.	n.p.	-
	7	2021.01.11-12.	20 weeks	AS	-	-	-	n.p.	n.p.	n.p.	-
	8	2021.01.11-12.	20 weeks	AS	-	-	-	n.p.	n.p.	n.p.	-
	9	2021.01.11-12.	20 weeks	AS	-	-	-	n.p.	n.p.	n.p.	-
	10	2021.01.11-12.	20 weeks	AS	-	-	-	n.p.	n.p.	n.p.	-
Hódmezővásárhely 1	10/1	2021.02.18	10 weeks	AS	-	-	-	n.p.	n.p.	n.p.	-
	10/2	2021.02.18	10 weeks	AS	-	-	-	n.p.	n.p.	n.p.	-
	10/3	2021.02.18	10 weeks	AS	38.16	-	-	-	n.p.	n.p.	-
	10/4	2021.02.18	10 weeks	AS	-	-	-	n.p.	n.p.	n.p.	-
	10/5	2021.02.18	10 weeks	AS	-	-	-	n.p.	n.p.	n.p.	-
	20/1	2021.02.18	20 weeks	AS	-	-	-	n.p.	n.p.	n.p.	-
	20/2	2021.02.18	20 weeks	AS	-	-	-	n.p.	n.p.	n.p.	-
	20/3	2021.02.18	20 weeks	AS	-	-	-	n.p.	n.p.	n.p.	-
	20/4	2021.02.18	20 weeks	AS	-	-	-	n.p.	n.p.	n.p.	-
	20/5	2021.02.18	20 weeks	AS	-	-	-	n.p.	n.p.	n.p.	-
Tiszaszentimre	70/1	2022.05.12	10 weeks	AS	-	-	-	n.p.	n.p.	n.p.	-
	70/2	2022.05.12	10 weeks	AS	-	-	-	n.p.	n.p.	n.p.	-
	70/3	2022.05.12	10 weeks	AS	-	-	-	n.p.	n.p.	n.p.	-
	70/4	2022.05.12	10 weeks	AS	-	-	-	n.p.	n.p.	n.p.	-
	70/5	2022.05.12	10 weeks	AS	-	-	-	n.p.	n.p.	n.p.	-
	134/1	2022.05.12	20 weeks	AS	-	-	-	n.p.	n.p.	n.p.	-
	134/2	2022.05.12	20 weeks	AS	-	-	-	n.p.	n.p.	n.p.	-
	134/3	2022.05.12	20 weeks	AS	-	-	-	n.p.	n.p.	n.p.	-
	134/4	2022.05.12	20 weeks	AS	-	-	-	n.p.	n.p.	n.p.	-
	134/5	2022.05.12	20 weeks	AS	-	-	-	n.p.	n.p.	n.p.	-
Poroszló	10/1	2022.06.10	10 weeks	AS	-	-	-	n.p.	n.p.	n.p.	-
	10/2	2022.06.10	10 weeks	AS	-	-	-	n.p.	n.p.	n.p.	-
	10/3	2022.06.10	10 weeks	AS	-	-	-	n.p.	n.p.	n.p.	-
	10/4	2022.06.10	10 weeks	AS	-	-	-	n.p.	n.p.	n.p.	-

	10/5	2022.06.10	10 weeks	AS	-	-	-	n.p.	n.p.	n.p.	-
	20/1	2022.06.10	20 weeks	AS	-	-	-	n.p.	n.p.	n.p.	-
	20/2	2022.06.10	20 weeks	AS	-	-	-	n.p.	n.p.	n.p.	-
	20/3	2022.06.10	20 weeks	AS	-	-	-	n.p.	n.p.	n.p.	-
	20/4	2022.06.10	20 weeks	AS	-	-	-	n.p.	n.p.	n.p.	-
	20/5	2022.06.10	20 weeks	AS	-	-	-	n.p.	n.p.	n.p.	-
Magyarhomorog	10/1	2022.06.	10 weeks	AS	-	-	-	n.p.	n.p.	n.p.	-
	10/2	2022.06.	10 weeks	AS	-	-	-	n.p.	n.p.	n.p.	-
	10/3	2022.06.	10 weeks	AS	-	-	-	n.p.	n.p.	n.p.	-
	10/4	2022.06.	10 weeks	AS	-	-	-	n.p.	n.p.	n.p.	-
	10/5	2022.06.	10 weeks	AS	-	-	-	n.p.	n.p.	n.p.	-
	20/1	2022.06.	20 weeks	AS	-	-	-	n.p.	n.p.	n.p.	-
	20/2	2022.06.	20 weeks	AS	-	-	-	n.p.	n.p.	n.p.	-
	20/3	2022.06.	20 weeks	AS	-	-	-	n.p.	n.p.	n.p.	-
	20/4	2022.06.	20 weeks	AS	-	-	-	n.p.	n.p.	n.p.	-
	20/5	2022.06.	20 weeks	AS	-	-	-	n.p.	n.p.	n.p.	-
Jászapáti	10/1	2021.11.19	10 weeks	AS	-	-	-	n.p.	n.p.	n.p.	-
(JA)	10/2	2021.11.19	10 weeks	AS	-	-	-	n.p.	n.p.	n.p.	-
	10/3	2021.11.19	10 weeks	AS	-	-	-	n.p.	n.p.	n.p.	-
	10/4	2021.11.19	10 weeks	AS	-	-	-	n.p.	n.p.	n.p.	-
	10/5	2021.11.19	10 weeks	AS	-	-	-	n.p.	n.p.	n.p.	-
	20/1	2021.11.19	20 weeks	AS	-	36.10	-	n.p.	+	n.p.	**OF-20/1-JA-NoV-3R-PCR2**
	20/2	2021.11.19	20 weeks	AS	-	38.24	-	n.p.	+#	n.p.	**OF-20/2-JA-NoV-3R-PCR2**
	20/3	2021.11.19	20 weeks	AS	-	34.11	-	n.p.	+#	n.p.	**OF-20/3-JA-NoV-3R-PCR2**
Pásztó	oral f. 10/1	2021.04.09	10 weeks	AS	-	-	-	n.p.	n.p.	n.p.	-
	oral f. 10/2	2021.04.09	10 weeks	AS	-	-	-	n.p.	n.p.	n.p.	-
	oral f. 10/3	2021.04.09	10 weeks	AS	-	-	-	n.p.	n.p.	n.p.	-
	oral f. 10/4	2021.04.09	10 weeks	AS	-	-	-	n.p.	n.p.	n.p.	-
	oral f. 10/5	2021.04.09	10 weeks	AS	-	-	-	n.p.	n.p.	n.p.	-
	oral f. 20/1	2021.04.09	20 weeks	AS	-	-	-	n.p.	n.p.	n.p.	-
	oral f. 20/2	2021.04.09	20 weeks	AS	-	-	-	n.p.	n.p.	n.p.	-
	oral f. 20/3	2021.04.09	20 weeks	AS	-	-	-	n.p.	n.p.	n.p.	-
	oral f. 20/4	2021.04.09	20 weeks	AS	-	-	-	n.p.	n.p.	n.p.	-

	oral f. 20/5	2021.04.09	20 weeks	AS	-	-	-	n.p.	n.p.	n.p.	-
Hódmezővásárhely 2 (HV2)	oral f. 10/1	2021.03.23	10 weeks	AS	-	-	36.01	n.p.	n.p.	-	-
	oral f. 10/2	2021.03.23	10 weeks	AS	-	-	-	n.p.	n.p.	n.p.	-
	oral f. 10/3	2021.03.23	10 weeks	AS	-	-	-	n.p.	n.p.	n.p.	-
	oral f. 10/4	2021.03.23	10 weeks	AS	-	-	-	n.p.	n.p.	n.p.	-
	oral f. 10/5	2021.03.23	10 weeks	AS	-	-	-	n.p.	n.p.	n.p.	-
	oral f. 20/1	2021.03.23	20 weeks	AS	-	-	-	n.p.	n.p.	n.p.	-
	oral f. 20/2	2021.03.23	20 weeks	AS	38.32	-	-	-	n.p.	n.p.	-
	oral f. 20/3	2021.03.23	20 weeks	AS	-	-	-	n.p.	n.p.	n.p.	-
	oral f. 20/4	2021.03.23	20 weeks	AS	-	-	-	n.p.	n.p.	n.p.	
	oral f. 20/5	2021.03.23	20 weeks	AS	-	33.35	-	n.p.	+#	n.p.	**OF-20/5-HV2-NoV-3R-PCR2**
Derecske	4W M1	2020.09.15	4 weeks	AS	36.89	-	-	n.p.	n.p.	n.p.	-
	4W M2	2020.09.15	5 weeks	AS	35.14	-	-	n.p.	n.p.	n.p.	-
	6W M1	2020.09.15	6 weeks	AS	35.05	-	-	n.p.	n.p.	n.p.	-
	6W M2	2020.09.15	6 weeks	AS	-	-	-	n.p.	n.p.	n.p.	-
	8W M1	2020.09.15	8 weeks	AS	-	-	-	n.p.	n.p.	n.p.	-
	8W M2	2020.09.15	8 weeks	AS	-	-	-	n.p.	n.p.	n.p.	-
	10 W M1	2020.09.15	10 weeks	AS	-	-	-	n.p.	n.p.	n.p.	-
	13 W	2020.09.15	13 weeks	AS	-	34.97	-	n.p.	n.p.	n.p.	-
	14 W	2020.09.15	14 weeks	AS	-	-	-	n.p.	n.p.	n.p.	-
	16 W	2020.09.15	16 weeks	AS	-	-	-	n.p.	n.p.	n.p.	-
	18 W	2020.09.15	18 weeks	AS	-	-	-	n.p.	n.p.	n.p.	-
	19 W	2020.09.15	19 weeks	AS	-	-	-	n.p.	n.p.	n.p.	-
Szlovákia (Nagyhegyes) (NH)	oral f. 1	2022.06.21	10 weeks	AS	-	-	-	n.p.	n.p.	n.p.	-
	oral f. 2	2022.06.21	10 weeks	AS	-	-	-	n.p.	n.p.	n.p.	-
	oral f. 3	2022.06.21	10 weeks	AS	-	-	-	n.p.	n.p.	n.p.	-
	oral f. 4	2022.06.21	10 weeks	AS	-	-	-	n.p.	n.p.	n.p.	-
	oral f. 5	2022.06.21	10 weeks	AS	-	-	-	n.p.	n.p.	n.p.	-
	oral f. 6	2022.06.21	20 weeks	AS	-	-	-	n.p.	n.p.	n.p.	-
	oral f. 7	2022.06.21	20 weeks	AS	-	-	-	n.p.	n.p.	n.p.	-
	oral f. 8	2022.06.21	20 weeks	AS	-	34.42	-	n.p.	+#	n.p.	**OF-8-NH-NoV-3R-PCR2**
	oral f. 9	2022.06.21	20 weeks	AS	-	34.29	34.54	n.p.	+#	+#	**OF-9-NH-NoV-3R-PCR2**

											**OF-9-NH-VaV-3R-PCR2**
	oral f. 10	2022.06.21	20 weeks	AS	-	-	-	n.p.	n.p.	n.p.	-
Prügy	10/1	2022.06.02	10 weeks	AS	-	-	-	n.p.	n.p.	n.p.	-
	10/2	2022.06.02	10 weeks	AS	-	-	-	n.p.	n.p.	n.p.	-
	10/3	2022.06.02	10 weeks	AS	-	-	-	n.p.	n.p.	n.p.	-
	10/4	2022.06.02	10 weeks	AS	-	-	-	n.p.	n.p.	n.p.	-
	10/5	2022.06.02	10 weeks	AS	-	-	-	n.p.	n.p.	n.p.	-
	16/1	2022.06.02	16 weeks	AS	-	-	-	n.p.	n.p.	n.p.	-
	16/2	2022.06.02	16 weeks	AS	-	-	-	n.p.	n.p.	n.p.	-
	16/3	2022.06.02	16 weeks	AS	-	-	-	n.p.	n.p.	n.p.	-
	16/4	2022.06.02	16 weeks	AS	-	-	-	n.p.	n.p.	n.p.	-
	16/5	2022.06.02	16 weeks	AS	-	-	-	n.p.	n.p.	n.p.	-
Zomba	oral f. 24	2020.10.22	2 weeks	AS	-	-	-	n.p.	n.p.	n.p.	-
	oral f. 44	2020.10.22	4 weeks	AS	32.10	-	-	+#	n.p.	n.p.	**OF-44-SaV-3R-PCR2**
	oral f. 64	2020.10.22	6 weeks	AS	32.33	-	-	n.p.	n.p.	n.p.	**-**
	oral f. 84	2020.10.22	8 weeks	AS	-	-	-	n.p.	n.p.	n.p.	-
	oral f. 104	2020.10.22	10 weeks	AS	-	-	-	n.p.	n.p.	n.p.	-
Szarvas	10 hetes 1	2021.05.28	10 weeks	AS	-	-	-	n.p.	n.p.	n.p.	-
	10 hetes 2	2021.05.28	10 weeks	AS	-	-	-	n.p.	n.p.	n.p.	-
	10 hetes 3	2021.05.28	10 weeks	AS	-	-	-	n.p.	n.p.	n.p.	-
	10 hetes 4	2021.05.28	10 weeks	AS	-	-	-	n.p.	n.p.	n.p.	-
	10 hetes 5	2021.05.28	10 weeks	AS	-	-	-	n.p.	n.p.	n.p.	-
	20 hetes 1	2021.05.28	20 weeks	AS	-	-	-	n.p.	n.p.	n.p.	-
	20 hetes 2	2021.05.28	20 weeks	AS	-	-	-	n.p.	n.p.	n.p.	-
	20 hetes 3	2021.05.28	20 weeks	AS	-	-	-	n.p.	n.p.	n.p.	-
	20 hetes 4	2021.05.28	20 weeks	AS	-	-	-	n.p.	n.p.	n.p.	-
	20 hetes 5	2021.05.28	20 weeks	AS	-	-	-	n.p.	n.p.	n.p.	-
Kunhegyes	E1	2021.12.10	10 weeks	AS	-	-	-	n.p.	n.p.	n.p.	-
	E2	2021.12.10	10 weeks	AS	-	-	-	n.p.	n.p.	n.p.	-
	E3	2021.12.10	10 weeks	AS	-	-	-	n.p.	n.p.	n.p.	-
	E4	2021.12.10	10 weeks	AS	-	-	-	n.p.	n.p.	n.p.	-
	E5	2021.12.10	10 weeks	AS	-	-	-	n.p.	n.p.	n.p.	-
	U1	2021.12.10	20 weeks	AS	-	-	-	n.p.	n.p.	n.p.	-

	U2	2021.12.10	20 weeks	AS	-	-	-	n.p.	n.p.	n.p.	-
	U3	2021.12.10	20 weeks	AS	-	-	-	n.p.	n.p.	n.p.	-
	U4	2021.12.10	20 weeks	AS	-	-	-	n.p.	n.p.	n.p.	-
	U5	2021.12.10	20 weeks	AS	-	-	-	n.p.	n.p.	n.p.	-
Szilfás	8h	2020.10.27	8 weeks	AS	-	-	-	n.p.	n.p.	n.p.	-
	9h	2020.10.27	9 weeks	AS	-	-	-	n.p.	n.p.	n.p.	-
	10h	2020.10.27	10 weeks	AS	-	-	-	n.p.	n.p.	n.p.	-
	11h	2020.10.27	11 weeks	AS	-	-	-	n.p.	n.p.	n.p.	-
	12h	2020.10.27	12 weeks	AS	-	-	-	n.p.	n.p.	n.p.	-
	17-19	2020.10.27	17-19 weeks	AS	-	-	-	n.p.	n.p.	n.p.	-
	21-24	2020.10.27	21-24 weeks	AS	-	-	-	n.p.	n.p.	n.p.	-
	23-28	2020.10.27	23-28 weeks	AS	-	-	-	n.p.	n.p.	n.p.	-
	29-30	2020.10.27	29-30 weeks	AS	-	-	-	n.p.	n.p.	n.p.	-
	35-36	2020.10.27	35-36 weeks	AS	-	-	-	n.p.	n.p.	n.p.	-
Harta (HA)	oral f. 12 hetes 1	2021.05.19	12 weeks	AS	-	-	-	n.p.	n.p.	n.p.	-
	oral f. 12 hetes 2	2021.05.19	12 weeks	AS	-	-	-	n.p.	n.p.	n.p.	-
	oral f. 12 hetes 3	2021.05.19	12 weeks	AS	-	-	-	n.p.	n.p.	n.p.	-
	oral f. 12 hetes 4	2021.05.19	12 weeks	AS	-	-	-	n.p.	n.p.	n.p.	-
	oral f. 12 hetes 5	2021.05.19	12 weeks	AS	-	-	-	n.p.	n.p.	n.p.	-
	oral f. 18 hetes 1	2021.05.19	18 weeks	AS	-	-	-	n.p.	n.p.	n.p.	-
	oral f. 18 hetes 2	2021.05.19	18 weeks	AS	-	-	-	n.p.	n.p.	n.p.	-
	oral f. 18 hetes 3	2021.05.19	18 weeks	AS	-	-	-	n.p.	n.p.	n.p.	-
	oral f. 18 hetes 4	2021.05.19	18 weeks	AS	-	-	-	n.p.	n.p.	n.p.	-
	oral f. 18 hetes 5	2021.05.19	18 weeks	AS	-	36.06	-	n.p.	+#	n.p.	**OF-18/5-HA-NoV-3R-PCR2**
Nagykonda	oral f. 1	2021.02.09.	10 weeks	AS	-	-	-	n.p.	n.p.	n.p.	-
	oral f. 2	2021.02.09.	10 weeks	AS	-	-	-	n.p.	n.p.	n.p.	-
	oral f. 3	2021.02.09.	20 weeks	AS	-	-	-	n.p.	n.p.	n.p.	-
	oral f. 4	2021.02.09.	20 weeks	AS	-	-	-	n.p.	n.p.	n.p.	-
Csátalja (CSA)	12/1	2021.05.19	12 weeks	AS	-	-	-	n.p.	n.p.	n.p.	-
	12/2	2021.05.19	12 weeks	AS	-	-	-	n.p.	n.p.	n.p.	-
	12/3	2021.05.19	12 weeks	AS	-	-	-	n.p.	n.p.	n.p.	-
	12/4	2021.05.19	12 weeks	AS	-	-	-	n.p.	n.p.	n.p.	-
	12/5	2021.05.19	12 weeks	AS	-	-	-	n.p.	n.p.	n.p.	-

	20/1	2021.05.19	20 weeks	AS	-	34.99	-	n.p.	+#	n.p.	**OF-20/1-CSA-NoV-3R-PCR2**
	20/2	2021.05.19	20 weeks	AS	-	-	-	n.p.	n.p.	n.p.	-
	20/3	2021.05.19	20 weeks	AS	-	35.94	-	n.p.	+	n.p.	**OF-20/3-CSA-NoV-3R-PCR2**
	20/4	2021.05.19	20 weeks	AS	-	34.23	-	n.p.	+#	n.p.	**OF-20/4-CSA-NoV-3R-PCR2**
	20/5	2021.05.19	20 weeks	AS	-	35.47	-	n.p.	+	n.p.	**OF-20/5-CSA-NoV-3R-PCR2**
Ormándlak	oral f. utón. 1	2020.12.01	10 weeks	AS	-	-	-	n.p.	n.p.	n.p.	-
(OL)	oral f. utón. 2	2020.12.01	10 weeks	AS	-	35.67	-	n.p.	+#	n.p.	**OF-U2-OL-NoV-3R-PCR2**
	oral f. utón. 3	2020.12.01	10 weeks	AS	-	-	-	n.p.	n.p.	n.p.	-
	oral f. utón. 4	2020.12.01	10 weeks	AS	-	-	-	n.p.	n.p.	n.p.	-
	oral f. utón. 5	2020.12.01	10 weeks	AS	-	-	-	n.p.	n.p.	n.p.	-
	oral f. hízó 1	2020.12.01	20 weeks	AS	-	-	-	n.p.	n.p.	n.p.	-
	oral f. hízó 2	2020.12.01	20 weeks	AS	29.34	-	-	+#	n.p.	n.p.	**OF-H2-SaV-3R-PCR2**
	oral f. hízó 3	2020.12.01	20 weeks	AS	-	-	-	n.p.	n.p.	n.p.	-
	oral f. hízó 4	2020.12.01	20 weeks	AS	-	-	-	n.p.	n.p.	n.p.	-
	oral f. hízó 5	2020.12.01	20 weeks	AS	33.81	-	-	n.p.	n.p.	n.p.	-
Kaba	oral f. B/2	2021.07.07	10 weeks	AS	-	-	-	n.p.	n.p.	n.p.	-
	oral f. B/3	2021.07.07	10 weeks	AS	-	-	-	n.p.	n.p.	n.p.	-
	oral f. B/5	2021.07.07	10 weeks	AS	-	-	-	n.p.	n.p.	n.p.	-
	oral f. B/6	2021.07.07	10 weeks	AS	-	-	-	n.p.	n.p.	n.p.	-
	oral f. B/11	2021.07.07	10 weeks	AS	-	-	-	n.p.	n.p.	n.p.	-
	oral f. H/1	2021.07.07	20 weeks	AS	-	-	-	n.p.	n.p.	n.p.	-
	oral f. H/2	2021.07.07	20 weeks	AS	-	-	-	n.p.	n.p.	n.p.	-
	oral f. H/3	2021.07.07	20 weeks	AS	-	-	-	n.p.	n.p.	n.p.	-
	oral f. H/4	2021.07.07	20 weeks	AS	-	-	-	n.p.	n.p.	n.p.	-
	oral f. H/4/2	2021.07.07	20 weeks	AS	-	-	-	n.p.	n.p.	n.p.	-
Sajószöged	10/1	2021.06.01	10 weeks	AS	-	-	-	n.p.	n.p.	n.p.	-
	10/2	2021.06.01	10 weeks	AS	-	-	-	n.p.	n.p.	n.p.	-
	10/3	2021.06.01	10 weeks	AS	-	-	-	n.p.	n.p.	n.p.	-
	10/4	2021.06.01	10 weeks	AS	38.49	-	-	n.p.	n.p.	n.p.	-
	10/5	2021.06.01	10 weeks	AS	-	-	-	n.p.	n.p.	n.p.	-
	20/1	2021.06.01	20 weeks	AS	-	-	-	n.p.	n.p.	n.p.	-
	20/2	2021.06.01	20 weeks	AS	-	-	-	n.p.	n.p.	n.p.	-
	20/3	2021.06.01	20 weeks	AS	-	-	-	n.p.	n.p.	n.p.	-

20/4	2021.06.01	20 weeks	AS	-	-	-	n.p.	n.p.	n.p.	-
20/5	2021.06.01	20 weeks	AS	-	-	-	n.p.	n.p.	n.p.	-

Table S3: Features of analyzed animals and its oral fluid (OF) samples and **s**ummaries of the results of triplex swine sapovirus(Sw-SaV)/Norovirus (Sw-NoV)/valovirus (Sw-VaV) RT-qPCR assays with measured Cq values as well as the results of 3'RACE semi-nested PCR (3'RACE-snPCR) reactions of selected qPCR positive samples. ID: identification, AS: asymptomatic animal. n.p.: not performed. #: 3'RACE-snPCR products were selected for NGS sequencing. In the 3RACE-snPCR product ID column the identification names of those of the 3'RACE-snPCR products were found which were selected for NGS sequencing.

3'RACE-snPCR product IDs	NGS data ID	No. of total reads	No. of Pos.Selected reads	No. of generated consensus sequences
**PS1-SaV-3R-PCR2 ***	269	43 451 368	36 574 122	2#
**III/1-SaV-3R-PCR2 ***	270	643 218	490 450	2#
**KE5-SaV-3R-PCR2 ***	271	643 820	588 382	1#
**KE6-SaV-3R-PCR2 ***	272	753 570	687 516	1#
**Du3-SaV-3R-PCR2 ***	273	610 050	520 784	2#
**Du6-SaV-3R-PCR2 ***	274	660 980	557 876	1#
**SzL4-SaV-3R-PCR2 ***	275	707 798	545 198	2#
**SzL9-SaV-3R-PCR2 ***	276	760 380	521 864	2#
**So3-SaV-3R-PCR2 ***	277	602 818	551 732	1#
**So5-SaV-3R-PCR2 ***	278	528 848	477 296	1#
**SzG8-SaV-3R-PCR2 ***	279	591 084	500 588	4#
**SzG10-SaV-3R-PCR2 ***	280	983 690	873 700	2#
**GD0717/1-SaV-3R-PCR2 ***	281	790 400	555 326	3#
**GD0717/3-SaV-3R-PCR2 ***	282	789 234	709 000	2#
**97/18-SaV-3R-PCR2 / Zsana1-NoV-3R-PCR2**	283	685 066	438 018	1/0
**GD12-SaV-3R-PCR2 / OF-9-NH-NoV-3R-PCR2**	284	730 192	613 524	0/2
**GD15-SaV-3R-PCR2 / OF-20/4-CSA-NoV-3R-PCR2**	285	720 384	663 886	1/1
**A2-SaV-3R-PCR2 / OF-20/5-HV2-NoV-3R-PCR2**	286	723 614	201 060	0/1
**A3-SaV-3R-PCR2 / OF-8-NH-NoV-3R-PCR2**	287	833 208	798 492	2/2
**C1-SaV-3R-PCR2 / OF-20/3-JA-NoV-3R-PCR2**	288	768 634	587 786	2/1
**C2-SaV-3R-PCR2 / PD1-VaV-3R-PCR2**	289	43 894 920	37 112 326	1/1
**W313/9-D8-SaV-3R-PCR2 / OF-9-NH-VaV-3R-PCR2**	290	928 434	531 102	1/1
**D3-SaV-3R-PCR2 / PS1-VaV-3R-PCR2**	291	740 998	505 640	2/1
**OF-44-SaV-3R-PCR2 / SzL1-VaV-3R-PCR2**	292	639 940	326 766	1/1
D1-SaV-3R-PCR2	293	712 538	458 634	1
OF-H2-SaV-3R-PCR2	294	766 210	583 479	5
W313/6-H4-SaV-3R-PCR2	295	670 920	-	0
OF-9H-JASZ-NoV-3R-PCR2	836	6 194 686	2 798 060	1
OF-121-ST-NoV-3R-PCR2	837	4 114 164	981 860	1
OF-20/2-JA-NoV-3R-PCR2	838	5 128 890	2 654 706	1
OF-18/5-HA-NoV-3R-PCR2	839	6 690 650	-	0
OF-20/1-CSA-NoV-3R-PCR2	840	5 714 978	1 304 500	1
OF-178-ST-NoV-3R-PCR2	841	5 918 812	1 549 746	1
OF-U2-OL-NoV-3R-PCR2	842	4 627 892	1 614 746	1
**∑=44**				**59**

Table S4: Summary of the results of next-generation sequencing reactions of selected 3’RACE semi-nested PCR (3’RACE-snPCR) products (details of the sequenced products can be found in Tables 7, S1 and S2). ID: identification number/name. Pos.Selected reads: Number of calicivirus reads identified from the total reads by positive selection (see Figure 6 for details). Consensus sequences were swine sapo-, noro-,or valovirus sequences acquired from the Pos.Selected reads by either reference-mapping based and/or de novo assembly-based approaches (see Figure 6 for details). 3’RACE-snPCR products from different viruses (SaV: sapovirus, NoV: norovirus, VaV: valovirus) of the same line (marked with bold and separated by “/” mark) in the 3'RACE-snPCR product IDs column were pooled samples. Consensus sequence numbers separated by a “/” mark represents the number of consensus sequences originated from the corresponding member of the pool found in the 3'RACE-snPCR product IDs column of the same line. Note that SaV 3’RACE-snPCR products marked with asterisk (*) were pooled with human NoV 3’RACE-snPCR products unrelated to this study. Consensus sequence numbers marked with (#) indicate numbers of only swine sapovirus contigs.

	intra-assay variation (CV %)				inter-assay variation (CV %)			
	Singleplex		Triplex		Singleplex		Triplex	
**Virus**	**1,00E+03**	**1,00E+04**	**1,00E+03**	**1,00E+04**	**1,00E+03**	**1,00E+04**	**1,00E+03**	**1,00E+04**
**Sw-SaV**	0.08-0.88	0.05-0.23	0.73-2.75	0.25-0.76	1.17	2.08	4.03	3.18
**Sw-NoV**	0.32-0.69	0.09-0.35	0.48-1.94	0.11-0.68	1.43	2.76	4.89	4.18
**Sw-VaV**	0.76-2.58	0.20-0.99	0.29-1.19	0.04-0.54	2.30	2.36	5.12	3.87

Table S5: Summary of the results of intra -, and inter assay variations assays.
Sw SaV: swine sapovirus, Sw NoV: swine norovirus, Sw VaV: swine valovirus.
CV %: Coefficient of Variation in percentages.

Virus type	Farm ID	Sample ID	Sample type	3RACE-snPCR product ID	Consensus sequence ID	Seq length (nt)	Accession No.	Mean coverage	Closest relative nt identity % (strain name, Acc.Num.)
Sapovirus (SaV)	Pusztaföldvár	PS-1@	enteric (DR)	PS1-SaV-3R-PCR2	swine/SaV/GVII [**PS1**-Var1/1-269] HUN/2009[#]	2227	PQ483450	23.4	84.98% (swine/WGP247/2009/USA, KC309421)
					swine/SaV/GIII [**PS1**-Var1/1-269] HUN/2009[#]	2252	PQ483451	1107166.0	96.18% (pig/Gansu/CH430/2012/CHN, KF204570)
	Ravazd	97/18@	enteric (DR)	97/18-SaV-3R-PCR2	swine/SaV/GIII [**97/18**-Var1/1-283] HUN/2018	2261	PQ483452	20248.3	91.03% (pig/Gansu/CH430/2012/CHN, KF204570)
	Városföld	A2	enteric (Non-DR)	A2-SaV-3R-PCR2	-	-	-	-	-
		A3	enteric (Non-DR)	A3-SaV-3R-PCR2	swine/SaV/GIII [**A3**-Var1/2-287] HUN/2016[#]	2261	PQ483453	21244.3	90.98% (pig/Gansu/CH430/2012/CHN, KF204570)
					swine/SaV/GIII [**A3**-Var2/2-287] HUN/2016	2252	PQ483454	562.0	84.99% (GIII-1ah, JX678943)
	Bácsalmás	C1	enteric (Non-DR)	C1-SaV-3R-PCR2	swine/SaV/GIII [**C1**-Var1/2-288] HUN/2016[#]	2261	PQ483455	10242.9	95.37% (PoSaV_VIRES_HLJ01_C1, MK378994)
					swine/SaV/GIII [**C1**-Var2/2-288] HUN/2016	2252	PQ483456	54.9	93.69% (pig/Gansu/CH430/2012/CHN, KF204570)
		C2	enteric (Non-DR)	C2-SaV-3R-PCR2	swine/SaV/GIII [**C2**-Var1/1-289] HUN/2016	2261	PQ483457	527882.3	95.33% (PoSaV_VIRES_HLJ01_C1, MK378994)
	Nyíribrony	D1	enteric (Non-DR)	D1-SaV-3R-PCR2	swine/SaV/GIII [**D1**-Var1/1-293] HUN/2016	2261	PQ483458	20723.0	94.09% (IL31538, MK965898)
		D3	enteric (Non-DR)	D3-SaV-3R-PCR2	swine/SaV/GIII [**D3**-Var1/2-291] HUN/2016[#]	2261	PQ483459	10263.8	94.13% (IL31538, MK965898)
					swine/SaV/GIII [**D3**-Var2/2-291] HUN/2016[#]	2252	PQ483460	278.3	94.96% (PF191/con/CalV5, OP413971)
	Dunaszekcső	Du3	enteric (DR)	Du3-SaV-3R-PCR2[*]	swine/SaV/GIII [**Du3**-Var1/2-273] HUN/2022[**#]	2252	PQ483461	6907.0	88.53% (SaV/GIII/USA/IA_4517-2/2019, MW316757)
					swine/SaV/GIII [**Du3**-Var2/2-273] HUN/2022[**#]	2261	PQ483462	6301.4	92.44% (SD220216, ON746330)
		Du6	enteric (DR)	Du6-SaV-3R-PCR2	swine/SaV/GIII [**Du6**-Var1/1-274] HUN/2022	2261	PQ483463	7804.6	88.12% (SaV/GIII/USA/IA_4517-2/2019, MW316757)
	Orosháza	GD0717/1	enteric (Non-DR)	GD0717/1-SaV-3R-PCR2[*]	swine/SaV/GIII [**GD0717/1**-Var1/3-281] HUN/2020[**#]	2252	PQ483464	7096.9	94.09% (pig/Gansu/CH430/2012/CHN, KF204570)
					swine/SaV/GIII [**GD0717/1**-Var2/3-281] HUN/2020[#]	2252	PQ483465	473.7	89.02% (MoI2-1-1, LC215874)
					swine/SaV/GIII [**GD0717/1**-Var3/3-281] HUN/2020	2261	PQ483466	288.3	94.79% (IL31538, MK965898)
		GD0717/3	enteric (Non-DR)	GD0717/3-SaV-3R-PCR2[*]	swine/SaV/GIII [**GD0717/3**-Var1/2-282] HUN/2020[**#]	2252	PQ483467	12606.0	94.63% (pig/Gansu/CH430/2012/CHN, KF204570)
					swine/SaV/GIII [**GD0717/3**-Var2/2-282] HUN/2020	2252	PQ483468	368.5	89.02% (MoI2-1-1, LC215874)

	GD15 Szk	enteric (Non-DR)	GD15-SaV-3R-PCR2	swine/SaV/GIII [**GD15**-Var1/1-285] HUN/2016[#]	2252	PQ483470	20598.4	89.12% (HgYa2-1, LC215881)
Szentkútpuszta	III/1@	enteric (Non-DR)	III/1-SaV-3R-PCR2	swine/SaV/GIII [**III/1**-Var1/2-270] HUN/2005[#]	2252	PQ483472	6194.3	91.16% (MoI2-1-1, LC215874)
				swine/SaV/GIII [**III/1**-Var2/2-270] HUN/2005[#]	2255	PQ483473	7694.6	88.69% (Ishi-Kah6, LC215879)
Kecel	KE-5	enteric (DR)	KE5-SaV-3R-PCR2	swine/SaV/GIII [**KE5**-Var1/1-271] HUN/2022	2264	PQ483474	14239.4	93.13% (PF183/con/CalV1, OP413967)
	KE-6	enteric (DR)	KE6-SaV-3R-PCR2	swine/SaV/GIII [**KE6**-Var1/1-272] HUN/2022	2264	PQ483475	15209.8	93.17% (SD220216, ON746330)
Somberek	So-3	enteric (DR)	So3-SaV-3R-PCR2	swine/SaV/GIII [**So3**-Var1/1-277] HUN/2022	2231	PQ483476	12656.1	91.57% (HgTa1, LC215883)
	So-5	enteric (DR)	So5-SaV-3R-PCR2	swine/SaV/GIII [**So5**-Var1/1-278] HUN/2022	2231	PQ483477	10953.4	91.39% (HgTa1, LC215883)
Szigetvár	SzG-10	enteric (DR)	SzG10-SaV-3R-PCR2[*]	swine/SaV/GIII [**SzG10**-Var1/2-280] HUN/2022[**#]	2261	PQ483478	13165.3	94.48% (PoSaV_VIRES_HLJ01_C1, MK378994)
				swine/SaV/GIII [**SzG10**-Var2/2-280] HUN/2022[#]	2252	PQ483479	6533.1	93.25% (pig/Gansu/CH430/2012/CHN, KF204570)
	SzG-8	enteric (DR)	SzG8-SaV-3R-PCR2	swine/SaV/GIII [**SzG8**-Var1/4-279] HUN/2022[#]	2255	PQ483480	6523.2	93.25% (PF183/con/CalV1, OP413967)
				swine/SaV/GIII [**SzG8**-Var2/4-279] HUN/2022	2261	PQ483481	1866.9	94.44% (PoSaV_VIRES_HLJ01_C1, MK378994)
				swine/SaV/GIII [**SzG8**-Var3/4-279] HUN/2022[#]	2252	PQ483482	2397.9	93.56% (pig/Gansu/CH430/2012/CHN, KF204570)
				swine/SaV/GIII [**SzG8**-Var4/4-279] HUN/2022[#]	2252	PQ483483	1090.5	93.23% (PF191/con/CalV5, OP413971)
Szilfás	SzL-4	enteric (DR)	SzL4-SaV-3R-PCR2[*]	swine/SaV/GIII [**SzL4**-Var1/2-275] HUN/2022[**#]	2252	PQ483484	4986.8	85.14% (Cowden, KT922087)
				swine/SaV/GIII [**SzL4**-Var2/2-275] HUN/2022	2261	PQ483485	617.6	93.86% (IL31538, MK965898)
	SzL-9	enteric (DR)	SzL9-SaV-3R-PCR2*	swine/SaV/GIII [**SzL9**-Var1/2-276] HUN/2022**[#]	2255	PQ483486	5294.4	92.85% (PF183/con/CalV1, OP413967)
				swine/SaV/GIII [**SzL9**-Var2/2-276] HUN/2022[#]	2252	PQ483487	2438.0	87.47% (226-k141_18826, MZ679046)
Mohács	W313/9-D8	enteric (Non-DR)	W313/9-D8-SaV-3R-PCR2	swine/SaV/GIII [**W313/9-D8**-Var1/1-290] HUN/2022	1830[&]	PQ483488	3452.2	93.53% (PF183/con/CalV1, OP413967)
	W313/6-H4	enteric (Non-DR)	W313/6-H4-SaV-3R-PCR2	-	-	-	-	-
Zomba	OF-44	oral fluid	OF-44-SaV-3R-PCR2	swine/SaV/GIII [**OF-44**-Var1/1-292] HUN/2020	2252	PQ483489	15969.6	93.38% (pig/Gansu/CH430/2012/CHN, KF204570)
Ormándlak	OF-H2	oral fluid	OF-H2-SaV-3R-PCR2*	swine/SaV/GIII [**OF-H2**-Var1/5-294] HUN/2020[**#]	2261	PQ483490	32354.9	94.97% (PoSaV_VIRES_HLJ01_C1, MK378994)

					swine/SaV/GIII [**OF-H2**-Var2/5-294] HUN/2020[#]	2231	PQ483491	2789.1	90.82% (HgTa1, LC215883)
					swine/SaV/GIII [**OF-H2**-Var3/5-294] HUN/2020[#]	2252	PQ483492	729.9	84.97% (TCA-Cowden, KT922088)
					swine/SaV/GIII [**OF-H2**-Var4/5-294] HUN/2020[#]	2252	PQ483493	320.9	89.70% (pig/Gansu/CH430/2012/CHN, KF204570)
					swine/SaV/GIII [**OF-H2**-Var5/5-294] HUN/2020	2252	PQ483494	190.4	94.56% (PF191/con/CalV5, OP413971)
Norovirus (NoV)	Nagyhegyes (Slovakia)	OF-9-NH	oral fluid	OF-9-NH-NoV-3R-PCR2	swine/NoV/GII.11 [**OF-9-NH**-284] SKV/2022[#]	2471	PQ467812	1624.8	87.01% (pig/GII/Ch6/China/2009, HQ392821)
					swine/NoV/GII.18 [**OF-9-NH**-284] SKV/2022[#]	2560	PQ467817	440.8	88.55% (OH-QW101/03/US, AY823304)
		OF-8-NH	oral fluid	OF-8-NH-NoV-3R-PCR2*	swine/NoV/GII.11 [**OF-8-NH**-287] SKV/2022**[#]	2471	PQ467813	10851.4	87.08% (pig/GII/Ch6/China/2009, HQ392821)
					swine/NoV/GII.18 [**OF-8-NH**-287] SKV/2022[#]	2560	PQ467818	229.0	88.67% (OH-QW101/03/US, AY823304)
	Jászárokszállás	OF-9H-JASZ	oral fluid	OF-9H-JASZ-NoV-3R-PCR2	swine/NoV/GII.11 [**OF-9H-JASZ**-836] HUN/2021	2477	PQ467807	132336.4	86.44% (pig/GII/Ch6/China/2009, HQ392821)
	Somogytarnóca	OF-121-ST	oral fluid	OF-121-ST-NoV-3R-PCR2	swine/NoV/GII.11 [**OF-121-ST**-837] HUN/2020	2478	PQ467811	4158.6	87.01% (pig/GII/Ch6/China/2009, HQ392821)
		OF-178-ST	oral fluid	OF-178-ST-NoV-3R-PCR2	swine/NoV/GII.18 [**OF-178-ST**-841] HUN/2020	2566	PQ467815	39882.8	87.23% (OH-QW125/03/US, AY823305)
	Csátalja	OF-20/1-CSA	oral fluid	OF-20/1-CSA-NoV-3R-PCR2	swine/NoV/GII.11 [**OF-20/1-CSA**-840] HUN/2021	2477	PQ467809	60288.5	86.88% (pig/GII/Ch6/China/2009, HQ392821)
		OF-20/4-CSA	oral fluid	OF-20/4-CSA-NoV-3R-PCR2	swine/NoV/GII.11 [**OF-20/4-CSA**-285] HUN/2021	2477	PQ467810	8786.3	86.88% (pig/GII/Ch6/China/2009, HQ392821)
	Hódmezővásárhely 2	OF-20/5-HV2	oral fluid	OF-20/5-HV2-NoV-3R-PCR2	swine/NoV/GII.11 [**OF-20/5-HV2**-286] HUN/2021	2479	PQ467808	8908.1	86.02% (MI-QW48/02/US, AY823303)
	Jászapáti	OF-20/2-JA	oral fluid	OF-20/2-JA-NoV-3R-PCR2	swine/NoV/GII.18 [**OF-20/2-JA**-838] HUN/2022	2564	PQ467816	119298.5	89.02% (OH-QW101/03/US, AY823304)
		OF-20/3-JA	oral fluid	OF-20/3-JA-NoV-3R-PCR2	swine/NoV/GII.18 [**OF-20/3-JA**-288] HUN/2022	2568	PQ467819	12243.6	89.02% (OH-QW101/03/US, AY823304)
	Ormándlak	OF-U2-OL	oral fluid	OF-U2-OL-NoV-3R-PCR2	swine/NoV/GII.18 [**OF-U2-OL**-842] HUN/2020	2559	PQ467814	75774.4	87.64% (OH-QW101/03/US, AY823304)
	Harta	OF-18/5-HA	oral fluid	OF-18/5-HA-NoV-3R-PCR2	-	-	-	-	-
	Zsana	Zsana-1@	enteric (Non-DR)	Zsana1-NoV-3R-PCR2	-	-	-	-	-
Valovirus (VaV)	Pusztaföldvár	PS-1@	enteric (DR)	PS1-VaV-3R-PCR2	swine/VaV/GI [**PS1**-269] HUN/2009	2128	PQ483495	2627.2	89.94% (AB104, FJ355930)
		PD-1@	enteric (Non-DR)	PD1-VaV-3R-PCR2	swine/VaV/GI [**PD1**-289] HUN/2009	2128	PQ483496	1119035.9	89.94% (AB104, FJ355930)
	Nagyhegyes (Slovakia)	OF-9-NH	oral fluid	OF-9-NH-VaV-3R-PCR2	swine/VaV/GI [**OF-9-NH**-290] SKV/2022	2128	PQ483497	20213.0	90.41% (St-Valerien swine virus, AB863586)

	Szilfás	SzL-1	enteric (DR)	SzL1-VaV-3R-PCR2	-	-	-	-	-

Table S6: Features of the sapovirus/norovirus/valovirus 3'RACE semi-nested (sn)PCR products used for next-generation sequencing and the consensus sequences identified from them. DR: sample from diarrheic animal, Non-DR: sample from non-diarrheic animal, ID: identification. The IDs of the 3RACE-snPCR products consists of the following parts: Sample ID followed by the virus type (SaV-sapovirus, NoV-norovirus, VaV-valovirus), 3R (3'RACE) and PCR2 (2nd round product of the semi-nested PCR reaction). The IDs of the consensus sequences consists of the following parts: host/virus type: (SaV/NoV/VaV)/ genogroup.type (type only in NoV) [sample ID followed by the Var: no. of sequence variant/total variants in the sample, the datafile ID] and HUN-Hungary/collection year (variant numbers were only included to the names of SaV sequences). Mean coverage: mean number of nucleotide coverage of all positions in the given consensus sequence calculated from the aligned reads by Geneious software (coverage maps of all consensus sequences can be found in Figure S2). *: selected 3'RACE-snPCR products which were also sequenced by Sanger-sequencing method. **: Consensus sequence which was nearly identical (>98% nt identity) to the corresponding Sanger-sequence of the PCR2* product found in the same line of the table. #: Consensus sequences which were verified by 3'RACE-PCR based on the use of variant-specific forward primers (see Table S5). &: The size of the consensus sequence is shorter compared to the other SaV consensuses (3' end of the ORF2 and the complete 3'UTR is missing) due to the mispriming of the oligo dT-Anchor-Adapter primer during RT. Closest relatives were identified from the results of individual BLASTn searches. Sample IDs marked with @ indicate faecal samples while all other, unmarked samples are individual rectal swabs.

Sapovirus	swine/SaV/GIII [97/18-Var1/1-283] HUN/2018	swine/SaV/GIII [A3-Var1/2-287] HUN/2016	swine/SaV/GIII [A3-Var2/2-287] HUN/2016	swine/SaV/GIII [C1-Var1/2-288] HUN/2016	swine/SaV/GIII [C1-Var2/2-288] HUN/2016	swine/SaV/GIII [C2-Var1/1-289] HUN/2016	swine/SaV/GIII [D1-Var1/1-293] HUN/2016	swine/SaV/GIII [D3-Var1/2-291] HUN/2016	swine/SaV/GIII [D3-Var2/2-291] HUN/2016	swine/SaV/GIII [Du3-Var2/2-273] HUN/2022	swine/SaV/GIII [Du3-Var1/2-273] HUN/2022	swine/SaV/GIII [Du6-Var1/1-274] HUN/2022	swine/SaV/GIII [GD0717/1-Var1/3-281] HUN/2020	swine/SaV/GIII [GD0717/1-Var2/3-281] HUN/2020	swine/SaV/GIII [GD0717/1-Var3/3-281] HUN/2020	swine/SaV/GIII [GD0717/3-Var1/2-282] HUN/2020	swine/SaV/GIII [GD0717/3-Var2/2-282] HUN/2020	swine/SaV/GIII [GD15-Var1/1-285] HUN/2016	swine/SaV/GIII [III/1-Var1/2-270] HUN/2005	swine/SaV/GIII [III/1-Var2/2-270] HUN/2005	swine/SaV/GIII [KE5-Var1/1-271] HUN/2022	swine/SaV/GIII [KE6-Var1/1-272] HUN/2022	swine/SaV/GIII [OF-44-Var1/1-292] HUN/2020	swine/SaV/GIII [OF-H2-Var1/5-294] HUN/2020	swine/SaV/GIII [OF-H2-Var2/5-294] HUN/2020	swine/SaV/GIII [OF-H2-Var3/5-294] HUN/2020	swine/SaV/GIII [OF-H2-Var4/5-294] HUN/2020	swine/SaV/GIII [OF-H2-Var5/5-294] HUN/2020	swine/SaV/GIII [PS1-Var1/1-269] HUN/2009	swine/SaV/GVII [PS1-Var1/1-269] HUN/2009	swine/SaV/GIII [So3-Var1/1-277] HUN/2022	swine/SaV/GIII [So5-Var1/1-278] HUN/2022	swine/SaV/GIII [SzG10-Var1/2-280] HUN/2022	swine/SaV/GIII [SzG10-Var2/2-280] HUN/2022	swine/SaV/GIII [SzG8-Var1/4-279] HUN/2022	swine/SaV/GIII [SzG8-Var2/4-279] HUN/2022	swine/SaV/GIII [SzG8-Var3/4-279] HUN/2022	swine/SaV/GIII [SzG8-Var4/4-279] HUN/2022	swine/SaV/GIII [SzL4-Var1/2-275] HUN/2022	swine/SaV/GIII [SzL4-Var2/2-275] HUN/2022	swine/SaV/GIII [SzL9-Var1/2-276] HUN/2022	swine/SaV/GIII [SzL9-Var2/2-276] HUN/2022	swine/SaV/GIII [W313/9-D8-Var1/1-290] HUN/2022	VP1 sequences
	100%	99.4%	82.96%	81.4%	90.34%	81.4%	81.52%	81.82%	83.32%	81.1%	81.28%	81.7%	90.64%	81.34%	81.58%	91%	81.4%	82.18%	82.42%	83.86%	80.86%	81.46%	91.06%	81.46%	82.66%	83.2%	90.28%	82%	91.78%	48.59%	82.42%	82.42%	82.06%	90.58%	80.86%	82.06%	91.06%	83.44%	82.24%	80.92%	80.14%	80.5%	81.22%	**swine/SaV/GIII [97/18-Var1/1-283] HUN/2018**
swine/SaV/GIII [97/18-Var1/1-283] HUN/2018	100%	100%	**82.72%**	81.28%	90.1%	81.28%	81.4%	81.7%	83.26%	81.1%	81.04%	81.46%	90.52%	81.16%	81.46%	90.88%	81.22%	82.06%	82.18%	83.86%	80.62%	81.34%	90.82%	81.34%	82.54%	82.84%	90.04%	81.94%	91.66%	48.65%	82.18%	82.18%	81.94%	90.28%	80.62%	81.94%	90.82%	83.26%	82%	80.8%	79.9%	80.26%	80.98%	**swine/SaV/GIII [A3-Var1/2-287] HUN/2016**
swine/SaV/GIII [A3-Var1/2-287] HUN/2016	98.39%	100%	100%	80.8%	83.62%	80.8%	80.44%	80.74%	81.88%	80.32%	80.38%	80.56%	82.9%	80.98%	80.86%	83.62%	81.04%	81.4%	82.18%	83.8%	80.38%	80.68%	83.38%	80.68%	82.06%	90.4%	83.92%	81.82%	83.68%	48.11%	82%	81.88%	80.68%	83.68%	80.2%	80.68%	83.86%	81.82%	90.94%	81.22%	80.02%	79.9%	80.5%	**swine/SaV/GIII [A3-Var2/2-287] HUN/2016**
swine/SaV/GIII [A3-Var2/2-287] HUN/2016	83.85%	**83.63%**	100%	100%	82.84%	100%	95.2%	95.08%	82.48%	90.64%	82.6%	81.82%	81.7%	82.42%	95.98%	81.82%	82.48%	89.5%	81.82%	82.72%	79.6%	90.76%	81.76%	97.84%	83.74%	80.86%	81.28%	81.46%	82.84%	47.69%	82.36%	82.36%	97.54%	82.66%	79.12%	97.42%	81.88%	81.88%	81.34%	96.88%	79.06%	80.38%	79.48%	**swine/SaV/GIII [C1-Var1/2-288] HUN/2016**
swine/SaV/GIII [C1-Var1/2-288] HUN/2016	84.67%	84.67%	82.2%	100%	100%	**82.84%**	82.54%	82.78%	83.02%	82.6%	81.88%	81.94%	92.62%	81.88%	82.6%	93.1%	81.94%	83.38%	82.78%	84.64%	80.38%	82.84%	91.6%	82.84%	83.26%	82.78%	90.04%	81.28%	94.12%	48.47%	83.08%	82.96%	83.14%	91.3%	80.56%	83.02%	91.84%	83.26%	82.9%	81.88%	80.32%	80.26%	80.26%	**swine/SaV/GIII [C1-Var2/2-288] HUN/2016**
swine/SaV/GIII [C1-Var2/2-288] HUN/2016	89.53%	89.36%	84.54%	**83.89%**	100%	100%	95.2%	95.08%	82.48%	90.64%	82.6%	81.82%	81.7%	82.42%	95.98%	81.82%	82.48%	89.5%	81.82%	82.72%	79.6%	90.76%	81.76%	97.84%	83.74%	80.86%	81.28%	81.46%	82.84%	47.69%	82.36%	82.36%	97.54%	82.66%	79.12%	97.42%	81.88%	81.88%	81.34%	96.88%	79.06%	80.38%	79.48%	**swine/SaV/GIII [C2-Var1/1-289] HUN/2016**
swine/SaV/GIII [C2-Var1/1-289] HUN/2016	84.63%	84.63%	82.24%	99.95%	83.94%	100%	100%	99.7%	82.24%	91%	82.48%	82.06%	81.52%	81.88%	94.78%	81.88%	81.94%	90.16%	81.58%	82.3%	79.96%	91.18%	82.18%	94.54%	84.16%	80.38%	81.7%	80.98%	82.96%	47.03%	82.96%	83.08%	94.12%	83.08%	79.84%	94.12%	82.3%	81.76%	81.04%	93.7%	79.54%	79.72%	79.84%	**swine/SaV/GIII [D1-Var1/1-293] HUN/2016**
swine/SaV/GIII [D1-Var1/1-293] HUN/2016	85.32%	85.32%	81.68%	94.48%	83.72%	94.53%	100%	100%	**82.12%**	91.06%	82.48%	82.06%	81.82%	81.94%	94.66%	82.18%	82%	89.98%	81.64%	82.48%	79.9%	91.24%	82.42%	94.42%	84.1%	80.68%	81.94%	81.04%	83.26%	46.97%	82.9%	83.02%	94%	83.38%	79.9%	94%	82.6%	81.82%	81.34%	93.58%	79.48%	79.66%	79.78%	**swine/SaV/GIII [D3-Var1/2-291] HUN/2016**
swine/SaV/GIII [D3-Var1/2-291] HUN/2016	85.41%	85.41%	81.81%	94.22%	84.24%	94.27%	99.26%	100%	100%	81.28%	82.3%	82.54%	83.38%	88.78%	82.3%	83.98%	88.84%	83.56%	91.48%	83.38%	79.84%	81.46%	82.24%	82.48%	82.9%	81.88%	83.26%	94.18%	84.22%	48.83%	82.78%	82.78%	82.54%	81.88%	80.2%	82.54%	82.42%	94.12%	81.1%	81.82%	80.02%	87.16%	79.84%	**swine/SaV/GIII [D3-Var2/2-291] HUN/2016**
swine/SaV/GIII [D3-Var2/2-291] HUN/2016	83.63%	83.59%	83.59%	82.94%	84.02%	82.98%	82.72%	**82.72%**	100%	100%	**82.66%**	81.28%	81.82%	80.98%	89.92%	81.58%	81.04%	88.54%	81.28%	81.7%	80.32%	97.66%	81.76%	90.7%	82.78%	79.9%	80.92%	80.5%	82.24%	46.79%	81.28%	81.16%	90.4%	81.76%	79.48%	90.28%	81.58%	80.86%	80.26%	89.62%	80.2%	79.12%	80.2%	**swine/SaV/GIII [Du3-Var2/2-273] HUN/2022**
swine/SaV/GIII [Du3-Var2/2-273] HUN/2022	84.28%	84.15%	81.16%	90.97%	83.37%	90.92%	90.92%	90.97%	81.64%	100%	100%	98.5%	81.28%	82.24%	82.72%	82.06%	82.3%	83.5%	82.3%	83.38%	80.44%	82.84%	81.04%	82.66%	89.26%	81.22%	81.76%	80.8%	82.48%	48.65%	90.1%	89.98%	82.6%	82.06%	80.74%	82.72%	82.06%	81.52%	80.38%	82.84%	80.62%	80.8%	80.26%	**swine/SaV/GIII [Du3-Var1/2-273] HUN/2022**
swine/SaV/GIII [Du3-Var1/2-273] HUN/2022	84.8%	84.54%	81.81%	85.63%	83.42%	85.59%	84.8%	84.76%	82.46%	**85.28%**	100%	100%	81.64%	82.36%	82.06%	82.42%	82.42%	83.32%	82.6%	83.62%	80.74%	81.7%	81.16%	82%	89.26%	81.94%	82.18%	81.16%	82.78%	48.53%	90.46%	90.34%	81.82%	81.88%	80.98%	81.94%	82.12%	81.64%	80.62%	82.24%	80.8%	80.74%	80.44%	**swine/SaV/GIII [Du6-Var1/1-274] HUN/2022**
swine/SaV/GIII [Du6-Var1/1-274] HUN/2022	84.85%	84.59%	81.46%	84.24%	83.33%	84.2%	84.59%	84.46%	82.29%	84.24%	97.3%	100%	100%	**81.28%**	**81.58%**	97.96%	81.22%	82.72%	82.6%	83.86%	80.5%	81.7%	92.14%	81.7%	83.02%	82.42%	89.92%	81.76%	94.96%	47.45%	82.48%	82.48%	82.24%	92.2%	80.26%	82.12%	92.68%	83.26%	83.14%	80.8%	80.14%	80.5%	80.44%	**swine/SaV/GIII [GD0717/1-Var1/3-281] HUN/2020**
swine/SaV/GIII [GD0717/1-Var1/3-281] HUN/2020	89.71%	89.62%	83.76%	82.98%	92.83%	83.02%	82.63%	83.24%	84.24%	82.85%	82.94%	82.72%	100%	100%	**82.78%**	82%	99.7%	82.36%	89.56%	82.9%	79%	80.92%	80.98%	82.54%	83.56%	80.62%	82.9%	87.76%	82.36%	48.05%	83.5%	83.5%	82.6%	82.24%	79.36%	82.6%	82.18%	87.94%	80.14%	81.76%	78.82%	86.02%	78.82%	**swine/SaV/GIII [GD0717/1-Var2/3-281] HUN/2020**
swine/SaV/GIII [GD0717/1-Var2/3-281] HUN/2020	81.94%	81.81%	82.85%	82.81%	82.76%	82.85%	82.07%	82.42%	88.62%	81.25%	82.5%	82.29%	**82.81%**	100%	100%	81.7%	82.84%	89.26%	81.76%	82.42%	79.84%	90.22%	81.94%	95.38%	83.68%	80.62%	81.28%	81.82%	83.08%	46.97%	82.78%	82.78%	95.02%	82.9%	79.54%	95.02%	82.12%	82%	81.76%	94.42%	79.6%	79.9%	79.42%	**swine/SaV/GIII [GD0717/1-Var3/3-281] HUN/2020**
swine/SaV/GIII [GD0717/1-Var3/3-281] HUN/2020	85.06%	84.89%	82.37%	95.87%	83.76%	95.83%	93.88%	93.79%	82.5%	90.53%	85.93%	84.63%	83.2%	**83.07%**	100%	100%	**81.94%**	82.66%	83.2%	84.04%	81.1%	81.58%	92.62%	81.7%	82.66%	82.84%	90.64%	82.06%	95.32%	48.17%	82.84%	82.84%	82.42%	92.26%	80.8%	82.3%	92.8%	83.62%	83.26%	81.64%	80.98%	80.86%	80.98%	**swine/SaV/GIII [GD0717/3-Var1/2-282] HUN/2020**
swine/SaV/GIII [GD0717/3-Var1/2-282] HUN/2020	90.01%	89.93%	84.41%	83.11%	93.4%	83.15%	83.02%	83.72%	84.54%	82.5%	83.37%	83.42%	97.96%	83.37%	83.24%	100%	100%	82.36%	89.62%	82.78%	79.06%	80.98%	81.04%	82.6%	83.68%	80.68%	82.96%	87.7%	82.42%	48.05%	83.68%	83.68%	82.66%	82.18%	79.06%	82.66%	82.12%	87.88%	80.2%	81.82%	78.88%	85.9%	79%	**swine/SaV/GIII [GD0717/3-Var2/2-282] HUN/2020**
swine/SaV/GIII [GD0717/3-Var2/2-282] HUN/2020	81.9%	81.77%	82.89%	82.85%	82.81%	82.89%	82.11%	82.46%	88.67%	81.2%	82.46%	82.24%	82.68%	99.69%	83.11%	**83.33%**	100%	100%	82.42%	83.86%	80.5%	88.54%	82.3%	89.08%	84.16%	81.7%	81.88%	82.54%	83.14%	48.11%	83.5%	83.5%	89.5%	82.42%	81.22%	89.5%	82.06%	82.84%	81.52%	88.84%	80.02%	81.7%	80.38%	**swine/SaV/GIII [GD15-Var1/1-285] HUN/2016**
swine/SaV/GIII [GD15-Var1/2-285] HUN/2016	83.68%	83.68%	82.89%	89.19%	84.41%	89.14%	89.32%	89.19%	84.02%	88.1%	84.76%	83.98%	83.85%	82.94%	88.93%	83.68%	82.94%	100%	100%	**82.66%**	79.54%	81.46%	82.06%	81.64%	82.84%	81.94%	82.54%	89.98%	83.26%	48.17%	82.72%	82.72%	81.94%	81.76%	79.42%	81.94%	81.82%	89.2%	81.46%	81.52%	79.24%	86.74%	79.54%	**swine/SaV/GIII [III/1-Var1/2-270] HUN/2005**
swine/SaV/GIII [III/1-Var1/2-270] HUN/2005	83.24%	83.07%	83.81%	82.76%	83.68%	82.81%	82.24%	82.68%	91.31%	81.77%	82.81%	82.85%	84.02%	90.27%	82.46%	84.5%	90.23%	83.07%	100%	100%	80.86%	81.94%	83.38%	82.72%	84.52%	83.86%	84.28%	82.78%	84.7%	47.93%	84.22%	84.1%	82.9%	83.98%	80.74%	82.9%	84.34%	83.38%	83.38%	82.84%	80.5%	80.86%	80.92%	**swine/SaV/GIII [III/1-Var2/2-270] HUN/2005**
swine/SaV/GIII [III/1-Var2/2-270] HUN/2005	83.94%	83.98%	84.59%	83.42%	84.8%	83.46%	82.68%	83.24%	85.5%	81.68%	83.68%	83.68%	84.67%	85.5%	82.85%	84.93%	85.41%	84.24%	**87.41%**	100%	100%	80.38%	80.32%	79.48%	80.86%	80.5%	80.5%	79.18%	80.38%	47.69%	82.12%	82%	79.78%	80.38%	91.9%	79.9%	80.5%	79.6%	80.44%	79.42%	94.66%	78.7%	98.92%	**swine/SaV/GIII [KE5-Var1/1-271] HUN/2022**
swine/SaV/GIII [KE5-Var1/1-271] HUN/2022	82.07%	81.9%	81.72%	81.42%	82.03%	81.46%	81.46%	81.51%	80.9%	81.38%	81.9%	82.03%	81.81%	79.99%	81.46%	82.42%	80.03%	82.16%	80.77%	81.51%	100%	100%	82.18%	90.7%	82.84%	80.02%	80.68%	80.68%	82.48%	46.61%	81.76%	81.64%	90.64%	81.82%	79.9%	90.58%	81.64%	80.98%	80.38%	89.86%	80.56%	79.42%	80.38%	**swine/SaV/GIII [KE6-Var1/1-272] HUN/2022**
swine/SaV/GIII [KE6-Var1/1-272] HUN/2022	84.98%	84.89%	82.2%	92.1%	84.11%	92.05%	91.71%	91.57%	82.37%	96.52%	86.41%	84.59%	83.2%	81.77%	91.53%	83.15%	81.81%	88.62%	82.68%	82.72%	81.85%	100%	100%	82.12%	82.78%	82.78%	89.92%	81.16%	93.4%	47.63%	81.64%	81.64%	82.12%	91.66%	79.96%	82.12%	92.02%	83.32%	83.02%	81.1%	80.68%	80.62%	80.62%	**swine/SaV/GIII [OF-44-Var1/1-292] HUN/2020**
swine/SaV/GIII [OF-44-Var1/1-292] HUN/2020	89.8%	89.62%	84.37%	83.02%	92.05%	83.07%	82.94%	83.33%	83.5%	82.85%	82.46%	82.24%	92.14%	82.2%	83.46%	92.53%	82.16%	83.2%	83.15%	83.98%	81.51%	83.59%	100%	100%	**83.86%**	**80.86%**	**81.46%**	**81.76%**	83.08%	47.69%	82.66%	82.66%	96.82%	82.9%	79.06%	96.7%	82.12%	81.82%	81.7%	96.22%	79.12%	80.26%	79.42%	**swine/SaV/GIII [OF-H2-Var1/5-294] HUN/2020**
swine/SaV/GIII [OF-H2-Var1/5-294] HUN/2020	84.72%	84.63%	81.98%	97.82%	83.89%	97.78%	93.83%	93.66%	82.98%	91.01%	85.76%	84.11%	83.33%	82.85%	95.44%	83.11%	82.89%	88.84%	82.63%	83.33%	81.16%	92.14%	83.5%	100%	100%	**82.72%**	**82.78%**	**82.48%**	83.5%	47.45%	92.14%	92.14%	83.62%	83.02%	81.04%	83.62%	83.26%	82.96%	83.02%	83.14%	80.44%	80.86%	80.92%	**swine/SaV/GIII [OF-H2-Var2/5-294] HUN/2020**
swine/SaV/GIII [OF-H2-Var2/5-294] HUN/2020	82.5%	82.42%	82.24%	82.98%	83.46%	82.98%	83.42%	83.63%	82.11%	82.72%	87.23%	87.19%	83.37%	82.5%	82.94%	83.24%	82.59%	84.15%	82.72%	84.11%	81.68%	82.46%	82.46%	**83.2%**	100%	100%	**82.6%**	**81.4%**	82.72%	47.93%	82.72%	82.6%	80.68%	82.24%	79.48%	80.68%	82.54%	81.4%	90.1%	80.62%	80.02%	79.54%	80.62%	**swine/SaV/GIII [OF-H2-Var3/5-294] HUN/2020**
swine/SaV/GIII [OF-H2-Var3/5-294] HUN/2020	83.46%	83.15%	90.62%	81.9%	83.42%	81.94%	81.29%	81.55%	83.11%	80.72%	81.68%	81.9%	82.94%	82.33%	81.9%	83.28%	82.37%	83.24%	83.15%	84.15%	81.77%	81.25%	83.24%	**81.85%**	**82.85%**	100%	100%	**81.58%**	91.66%	48.59%	82.78%	82.66%	81.88%	90.34%	80.44%	81.76%	90.64%	83.14%	83.62%	81.46%	80.44%	80.92%	80.86%	**swine/SaV/GIII [OF-H2-Var4/5-294] HUN/2020**
swine/SaV/GIII [OF-H2-Var4/5-294] HUN/2020	88.8%	88.54%	84.46%	81.94%	88.84%	81.9%	82.46%	82.63%	84.85%	81.64%	82.11%	82.46%	88.49%	84.5%	81.94%	89.06%	84.54%	82.68%	84.41%	85.5%	81.2%	81.94%	89.01%	**82.24%**	**82.29%**	**83.24%**	100%	100%	81.64%	47.57%	81.76%	81.76%	81.34%	80.74%	79.48%	81.46%	80.92%	91.9%	81.28%	80.92%	79.3%	85.66%	78.94%	**swine/SaV/GIII [OF-H2-Var5/5-294] HUN/2020**
swine/SaV/GIII [OF-H2-Var5/5-294] HUN/2020	82.16%	82.2%	83.2%	81.81%	82.24%	81.85%	80.94%	81.38%	94.18%	80.51%	81.16%	81.11%	82.59%	88.19%	81.9%	82.85%	88.15%	82.81%	90.27%	85.02%	80.33%	81.25%	82.2%	**81.98%**	**81.94%**	**82.42%**	**83.28%**	100%	100%	**48.05%**	83.02%	83.02%	82.96%	94.24%	80.74%	82.84%	94.78%	83.44%	82.96%	82.3%	80.56%	80.74%	80.62%	**swine/SaV/GIII [PS1-Var1/1-269] HUN/2009**
swine/SaV/GIII [PS1-Var1/1-269] HUN/2009	90.75%	90.75%	84.93%	84.02%	94.66%	84.07%	84.07%	84.76%	85.15%	83.11%	83.76%	83.76%	95%	83.59%	84.33%	95.48%	83.55%	84.15%	84.59%	85.28%	81.94%	83.89%	93.61%	84.2%	83.68%	83.5%	90.1%	82.85%	100%	100%	48.35%	48.35%	47.81%	48.11%	47.99%	47.69%	48.23%	48.65%	47.15%	47.45%	47.45%	48.89%	47.87%	**swine/SaV/GVII [PS1-Var1/1-269] HUN/2009**
swine/SaV/GVII [PS1-Var1/1-269] HUN/2009	47.04%	47.13%	46.78%	46.13%	46.87%	46.13%	45.78%	45.65%	47.04%	45.35%	46.83%	46.61%	46.39%	46.61%	45.74%	46.83%	46.52%	46.57%	46.22%	46.09%	46.22%	45.18%	46.35%	46.05%	45.57%	46.48%	46.87%	46%	**46.74%**	100%	100%	99.88%	83.02%	82.66%	82.12%	83.02%	82.78%	82.42%	82.78%	82.3%	81.34%	80.92%	82.12%	**swine/SaV/GIII [So3-Var1/1-277] HUN/2022**
swine/SaV/GIII [So3-Var1/1-277] HUN/2022	82.5%	82.42%	82.5%	82.29%	83.5%	82.29%	82.76%	82.89%	82.24%	81.64%	87.93%	88.02%	83.15%	83.07%	82.46%	83.37%	83.2%	83.89%	82.72%	83.85%	82.68%	81.77%	81.94%	82.33%	92.49%	82.89%	82.16%	81.59%	83.46%	46.13%	100%	100%	82.9%	82.66%	82%	82.9%	82.78%	82.54%	82.66%	82.3%	81.34%	80.92%	82.12%	**swine/SaV/GIII [So5-Var1/1-278] HUN/2022**
swine/SaV/GIII [So5-Var1/1-278] HUN/2022	82.5%	82.42%	82.42%	82.29%	83.42%	82.29%	82.85%	82.98%	82.24%	81.55%	87.84%	87.93%	83.15%	83.02%	82.46%	83.37%	83.15%	83.89%	82.72%	83.76%	82.59%	81.68%	81.94%	82.33%	92.53%	82.81%	82.11%	81.59%	83.46%	46.13%	99.82%	100%	100%	**82.9%**	79.42%	99.88%	82.12%	81.82%	81.7%	95.44%	79.18%	80.2%	79.78%	**swine/SaV/GIII [SzG10-Var1/2-280] HUN/2022**
swine/SaV/GIII [SzG10-Var1/2-280] HUN/2022	85.06%	84.98%	81.98%	97.78%	84.2%	97.74%	93.53%	93.27%	82.81%	90.75%	85.46%	84.2%	83.33%	82.68%	94.92%	83.5%	82.72%	89.06%	82.76%	83.37%	81.59%	91.84%	83.37%	96.83%	82.76%	81.64%	82.42%	81.46%	83.98%	46.13%	82.59%	82.5%	100%	100%	80.68%	82.78%	99.22%	83.86%	82.66%	81.94%	80.14%	80.38%	80.5%	**swine/SaV/GIII [SzG10-Var2/2-280] HUN/2022**
swine/SaV/GIII [SzG10-Var2/2-280] HUN/2022	89.23%	89.1%	84.5%	83.72%	92.18%	83.76%	83.59%	84.07%	82.98%	82.46%	82.98%	82.42%	92.66%	83.15%	83.85%	92.75%	83.2%	83.2%	82.89%	84.33%	81.55%	83.15%	91.84%	83.98%	82.85%	82.89%	88.58%	81.81%	94.44%	46.7%	82.81%	82.81%	**83.81%**	100%	100%	**79.54%**	**81.04%**	**79.78%**	80.02%	79.36%	91.66%	78.76%	92.2%	**swine/SaV/GIII [SzG8-Var1/4-279] HUN/2022**
swine/SaV/GIII [SzG8-Var1/4-279] HUN/2022	82.11%	81.94%	81.29%	80.94%	82.11%	80.98%	81.46%	81.64%	81.33%	80.98%	82.29%	82.46%	81.81%	80.29%	81.59%	82.24%	79.99%	82.5%	80.59%	81.25%	91.88%	81.46%	81.42%	80.81%	81.64%	80.68%	80.68%	80.59%	82.07%	46.57%	82.5%	82.42%	81.07%	81.72%	100%	100%	**82%**	**81.82%**	81.7%	95.44%	79.3%	80.32%	79.9%	**swine/SaV/GIII [SzG8-Var2/4-279] HUN/2022**
swine/SaV/GIII [SzG8-Var2/4-279] HUN/2022	85.02%	84.93%	81.94%	97.65%	84.15%	97.61%	93.48%	93.31%	82.76%	90.71%	85.59%	84.33%	83.28%	82.72%	94.96%	83.46%	82.76%	89.01%	82.81%	83.42%	81.64%	91.75%	83.42%	96.78%	82.81%	81.68%	82.29%	81.59%	83.94%	46.09%	82.55%	82.46%	99.86%	83.76%	**81.2%**	100%	100%	**84.52%**	82.96%	81.34%	80.44%	80.56%	80.8%	**swine/SaV/GIII [SzG8-Var3/4-279] HUN/2022**
swine/SaV/GIII [SzG8-Var3/4-279] HUN/2022	89.62%	89.53%	84.67%	83.11%	92.62%	83.15%	83.07%	83.55%	83.42%	82.29%	83.02%	82.55%	93.05%	83.15%	83.33%	93.18%	83.2%	82.98%	82.89%	84.54%	81.59%	82.98%	92.05%	83.37%	82.98%	83.15%	88.84%	81.98%	94.87%	46.83%	82.85%	82.85%	83.2%	99.39%	**81.94%**	**83.15%**	100%	100%	81.28%	81.1%	79.84%	85.9%	79.9%	**swine/SaV/GIII [SzG8-Var4/4-279] HUN/2022**
swine/SaV/GIII [SzG8-Var4/4-279] HUN/2022	83.55%	83.42%	83.55%	82.59%	84.28%	82.63%	81.94%	82.37%	94.18%	81.33%	81.98%	81.85%	84.24%	88.41%	82.29%	84.63%	88.45%	83.81%	90.06%	85.98%	80.9%	82.07%	84.07%	82.5%	82.72%	83.07%	85.02%	92.36%	84.54%	46.48%	82.2%	82.29%	82.42%	84.5%	**80.85%**	**82.46%**	**84.93%**	100%	100%	**81.16%**	79.48%	79.06%	80.32%	**swine/SaV/GIII [SzL4-Var1/2-275] HUN/2022**
swine/SaV/GIII [SzL4-Var1/2-275] HUN/2022	82.85%	82.63%	91.36%	82.33%	83.46%	82.37%	81.85%	82.07%	82.33%	80.98%	81.33%	81.2%	83.63%	81.85%	82.63%	83.94%	81.9%	82.85%	83.15%	84.24%	81.85%	81.68%	83.37%	82.2%	83.24%	90.53%	84.11%	82.29%	83.76%	46%	83.07%	82.98%	82.29%	83.2%	80.9%	82.24%	83.37%	82.72%	100%	100%	79.3%	79.96%	79.18%	**swine/SaV/GIII [SzL4-Var2/2-275] HUN/2022**
swine/SaV/GIII [SzL4-Var2/2-275] HUN/2022	84.28%	84.2%	82.33%	96.74%	82.76%	96.7%	93.09%	92.88%	82.11%	89.8%	85.54%	84.11%	82.16%	82.07%	94.61%	82.89%	82.11%	88.41%	82.2%	82.98%	80.9%	91.05%	82.29%	96.18%	82.24%	81.64%	82.03%	80.98%	83.5%	45.65%	81.81%	81.81%	95.35%	82.94%	80.59%	95.31%	82.55%	81.68%	**81.77%**	100%	100%	**78.52%**	94.66%	**swine/SaV/GIII [SzL9-Var1/2-276] HUN/2022**
swine/SaV/GIII [SzL9-Var1/2-276] HUN/2022	81.59%	81.42%	81.33%	81.03%	82.2%	81.07%	81.25%	81.29%	81.2%	81.55%	82.24%	82.2%	81.81%	79.94%	81.42%	82.42%	79.99%	82.03%	80.59%	81.33%	94.79%	82.24%	81.94%	81.11%	81.38%	81.38%	81.07%	80.33%	81.85%	46.22%	82.2%	82.2%	81.16%	81.64%	91.75%	81.29%	81.81%	81.33%	80.98%	80.81%	100%	100%	78.58%	**swine/SaV/GIII [SzL9-Var2/2-276] HUN/2022**
swine/SaV/GIII [SzL9-Var2/2-276] HUN/2022	81.46%	81.29%	81.38%	81.42%	81.46%	81.46%	80.72%	80.98%	87.8%	80.03%	81.59%	81.42%	81.94%	87.32%	80.9%	82.29%	87.15%	81.94%	87.93%	83.72%	79.55%	80.77%	82.07%	81.29%	80.51%	80.9%	82.98%	87.32%	82.24%	46.83%	81.07%	81.11%	81.03%	81.38%	80.2%	81.16%	81.46%	87.02%	80.68%	80.77%	**79.38%**	100%	100%	**swine/SaV/GIII [W313/9-D8-Var1/1-290] HUN/2022**
swine/SaV/GIII [W313/9-D8-Var1/1-290] HUN/2022	66.31%	66.18%	66.18%	65.4%	66.1%	65.4%	65.66%	65.79%	65.62%	66.4%	65.88%	66.05%	66.23%	65.32%	65.4%	66.66%	65.49%	66.62%	65.88%	66.66%	80.55%	65.97%	66.36%	65.4%	67.88%	66.36%	66.4%	65.14%	66.44%	40.79%	68.7%	68.7%	65.66%	66.36%	75%	65.79%	66.53%	66.1%	66.1%	65.14%	77.3%	64.62%	100%	
**Complete consensus sequences**	swine/SaV/GIII [97/18-Var1/1-283] HUN/2018	swine/SaV/GIII [A3-Var1/2-287] HUN/2016	swine/SaV/GIII [A3-Var2/2-287] HUN/2016	swine/SaV/GIII [C1-Var1/2-288] HUN/2016	swine/SaV/GIII [C1-Var2/2-288] HUN/2016	swine/SaV/GIII [C2-Var1/1-289] HUN/2016	swine/SaV/GIII [D1-Var1/1-293] HUN/2016	swine/SaV/GIII [D3-Var1/2-291] HUN/2016	swine/SaV/GIII [D3-Var2/2-291] HUN/2016	swine/SaV/GIII [Du3-Var2/2-273] HUN/2022	swine/SaV/GIII [Du3-Var1/2-273] HUN/2022	swine/SaV/GIII [Du6-Var1/1-274] HUN/2022	swine/SaV/GIII [GD0717/1-Var1/3-281] HUN/2020	swine/SaV/GIII [GD0717/1-Var2/3-281] HUN/2020	swine/SaV/GIII [GD0717/1-Var3/3-281] HUN/2020	swine/SaV/GIII [GD0717/3-Var1/2-282] HUN/2020	swine/SaV/GIII [GD0717/3-Var2/2-282] HUN/2020	swine/SaV/GIII [GD15-Var1/2-285] HUN/2016	swine/SaV/GIII [III/1-Var1/2-270] HUN/2005	swine/SaV/GIII [III/1-Var2/2-270] HUN/2005	swine/SaV/GIII [KE5-Var1/1-271] HUN/2022	swine/SaV/GIII [KE6-Var1/1-272] HUN/2022	swine/SaV/GIII [OF-44-Var1/1-292] HUN/2020	swine/SaV/GIII [OF-H2-Var1/5-294] HUN/2020	swine/SaV/GIII [OF-H2-Var2/5-294] HUN/2020	swine/SaV/GIII [OF-H2-Var3/5-294] HUN/2020	swine/SaV/GIII [OF-H2-Var4/5-294] HUN/2020	swine/SaV/GIII [OF-H2-Var5/5-294] HUN/2020	swine/SaV/GIII [PS1-Var1/1-269] HUN/2009	swine/SaV/GVII [PS1-Var1/1-269] HUN/2009	swine/SaV/GIII [So3-Var1/1-277] HUN/2022	swine/SaV/GIII [So5-Var1/1-278] HUN/2022	swine/SaV/GIII [SzG10-Var1/2-280] HUN/2022	swine/SaV/GIII [SzG10-Var2/2-280] HUN/2022	swine/SaV/GIII [SzG8-Var1/4-279] HUN/2022	swine/SaV/GIII [SzG8-Var2/4-279] HUN/2022	swine/SaV/GIII [SzG8-Var3/4-279] HUN/2022	swine/SaV/GIII [SzG8-Var4/4-279] HUN/2022	swine/SaV/GIII [SzL4-Var1/2-275] HUN/2022	swine/SaV/GIII [SzL4-Var2/2-275] HUN/2022	swine/SaV/GIII [SzL9-Var1/2-276] HUN/2022	swine/SaV/GIII [SzL9-Var2/2-276] HUN/2022	swine/SaV/GIII [W313/9-D8-Var1/1-290] HUN/2022	

Table S7: Pairwise nucleotide identity values in percentages between complete sapovirus consensus sequences (lower part of the table with clear cells) and sapovirus VP1 sequences (upper part of the table, grey cells) identified in this study. Percentage values in bold and red are identity values between sequences of the same sample. Pairwise identity values between VP1 sequences of the same sample higher than the genotype demarcation limit (83.10%, Oka et al. [15]) are highlighted with yellow colour.

Norovirus	swine/NoV/GII.11 [OF-20/4-CSA-285] HUN/2021	swine/NoV/GII.11 [OF-20/1-CSA-840] HUN/2021	swine/NoV/GII.11 [OF-121-ST-837] HUN/2020	swine/NoV/GII.11 [OF-20/5-HV2-286] HUN/2021	swine/NoV/GII.11 [OF-9H-JASZ-836] HUN/2021	swine/NoV/GII.11 [OF-9-NH-284] SKV/2022	swine/NoV/GII.11 [OF-8-NH-287] SKV/2022	swine/NoV/GII.18 [OF-9-NH-284] SKV/2022	swine/NoV/GII.18 [OF-8-NH-287] SKV/2022	swine/NoV/GII.18 [OF-20/3-JA-288] HUN/2022	swine/NoV/GII.18 [OF-20/2-JA-838] HUN/2022	swine/NoV/GII.18 [OF-U2-OL-842] HUN/2020	swine/NoV/GII.18 [OF-178-ST-841] HUN/2020	VP1 sequences
	100%	100%	89.54%	89.12%	89.78%	87.09%	86.91%	65.89%	65.71%	66.24%	66.24%	65.89%	65.17%	swine/NoV/GII.11 [OF-20/4-CSA-285] HUN/2021
swine/NoV/GII.11 [OF-20/4-CSA-285] HUN/2021	100%	100%	89.54%	89.12%	89.78%	87.09%	86.91%	65.89%	65.71%	66.24%	66.24%	65.89%	65.17%	swine/NoV/GII.11 [OF-20/1-CSA-840] HUN/2021
swine/NoV/GII.11 [OF-20/1-CSA-840] HUN/2021	100%	100%	100%	88.82%	89.30%	86.85%	86.91%	65.65%	65.47%	66.18%	66.18%	65.65%	64.63%	swine/NoV/GII.11 [OF-121-ST-837] HUN/2020
swine/NoV/GII.11 [OF-121-ST-837] HUN/2020	89.38%	89.38%	100%	100%	90.26%	87.03%	86.85%	65.29%	65.23%	65.65%	65.65%	65.23%	65.41%	swine/NoV/GII.11 [OF-20/5-HV2-286] HUN/2021
swine/NoV/GII.11 [OF-20/5-HV2-286] HUN/2021	89.38%	89.38%	88.64%	100%	100%	87.87%	87.81%	66.18%	66.12%	66.48%	66.48%	66.00%	65.05%	swine/NoV/GII.11 [OF-9H-JASZ-836] HUN/2021
swine/NoV/GII.11 [OF-9H-JASZ-836] HUN/2021	89.69%	89.69%	89.5%	90.23%	100%	100%	98.86%	**65.65%**	65.47%	65.83%	65.83%	65.71%	65.65%	swine/NoV/GII.11 [OF-9-NH-284] SKV/2022
swine/NoV/GII.11 [OF-9-NH-284] SKV/2022	86.74%	86.74%	85.78%	86.01%	86.59%	100%	100%	65.41%	**65.23%**	65.59%	65.59%	65.53%	65.59%	swine/NoV/GII.11 [OF-8-NH-287] SKV/2022
swine/NoV/GII.11 [OF-8-NH-287] SKV/2022	86.59%	86.59%	85.85%	85.85%	86.74%	98.91%	100%	100%	99.76%	89.30%	89.30%	86.02%	86.20%	swine/NoV/GII.18 [OF-9-NH-284] SKV/2022
swine/NoV/GII.18 [OF-9-NH-284] SKV/2022	63.54%	63.54%	62.96%	62.92%	64.08%	**62.41%**	62.22%	100%	100%	89.18%	89.18%	85.90%	86.08%	swine/NoV/GII.18 [OF-8-NH-287] SKV/2022
swine/NoV/GII.18 [OF-8-NH-287] SKV/2022	63.42%	63.42%	62.88%	62.88%	64.04%	62.30%	**62.10%**	99.65%	100%	100%	100%	86.55%	87.51%	swine/NoV/GII.18 [OF-20/3-JA-288] HUN/2022
swine/NoV/GII.18 [OF-20/3-JA-288] HUN/2022	63.69%	63.69%	63.15%	62.92%	63.85%	62.30%	62.14%	88.53%	88.41%	100%	100%	86.55%	87.51%	swine/NoV/GII.18 [OF-20/2-JA-838] HUN/2022
swine/NoV/GII.18 [OF-20/2-JA-838] HUN/2022	63.54%	63.54%	62.99%	62.76%	63.69%	62.14%	61.99%	88.37%	88.26%	99.84%	100%	100%	87.75%	swine/NoV/GII.18 [OF-U2-OL-842] HUN/2020
swine/NoV/GII.18 [OF-U2-OL-842] HUN/2020	63.3%	63.3%	63.19%	62.76%	63.46%	62.57%	62.49%	85.74%	85.54%	86.05%	85.89%	100%	100%	swine/NoV/GII.18 [OF-178-ST-841] HUN/2020
swine/NoV/GII.18 [OF-178-ST-841] HUN/2020	63.11%	63.11%	62.22%	63.03%	62.68%	62.61%	62.72%	85.85%	85.74%	86.98%	86.82%	87.60%	100%	
**Complete consensus sequences**	swine/NoV/GII.11 [OF-20/4-CSA-285] HUN/2021	swine/NoV/GII.11 [OF-20/1-CSA-840] HUN/2021	swine/NoV/GII.11 [OF-121-ST-837] HUN/2020	swine/NoV/GII.11 [OF-20/5-HV2-286] HUN/2021	swine/NoV/GII.11 [OF-9H-JASZ-836] HUN/2021	swine/NoV/GII.11 [OF-9-NH-284] SKV/2022	swine/NoV/GII.11 [OF-8-NH-287] SKV/2022	swine/NoV/GII.18 [OF-9-NH-284] SKV/2022	swine/NoV/GII.18 [OF-8-NH-287] SKV/2022	swine/NoV/GII.18 [OF-20/3-JA-288] HUN/2022	swine/NoV/GII.18 [OF-20/2-JA-838] HUN/2022	swine/NoV/GII.18 [OF-U2-OL-842] HUN/2020	swine/NoV/GII.18 [OF-178-ST-841] HUN/2020	

Table S8: Pairwise nucleotide identity values in percentages between complete norovirus consensus sequences (lower part of the table with clear cells) and norovirus VP1 sequences (upper part of the table, grey cells) identified in this study. Percentage values in bold and red are identity values between sequences of the same sample.

Valovirus	swine/VaV/GI [PS1-269] HUN/2009	swine/VaV/GI [PD1-289] HUN/2009	swine/VaV/GI [OF-9-NH-290] SKV/2022	VP1 sequences
	100%	100%	88.39%	swine/VaV/GI [PS1-269] HUN/2009
swine/VaV/GI [PS1-269] HUN/2009	100%	100%	88.39%	swine/VaV/GI [PD1-289] HUN/2009
swine/VaV/GI [PD1-289] HUN/2009	99.95%	100%	100%	swine/VaV/GI [OF-9-NH-290] SKV/2022
swine/VaV/GI [OF-9-NH-290] SKV/2022	88.67%	88.72%	100%	
**Complete consensus sequences**	swine/VaV/GI [PS1-269] HUN/2009	swine/VaV/GI [PD1-289] HUN/2009	swine/VaV/GI [OF-9-NH-290] SKV/2022	

Table S9: Pairwise nucleotide identity values in percentages between complete valovirus consensus sequences (lower part of the table with clear cells) and valovirus VP1 sequences (upper part of the table, grey cells) identified in this study.

Target virus	Reaction type	Oligonucleotide ID	Sequence (5' - 3')	3' RACE RT-PCR product size
Sapovirus (SaV)	3'RACE PCR	*Sw-SaV-Ell-F1*	AAT GCG GGC AAG GTG GTG GC	1340 bp
		Sw-SaV-Ell-F2	GAG GGT CTA GCA TAT CTG ACA AT	1220 bp
		Sw-SaV-Ell-F3	CAC TGC AAA CAC CAT ATC AGA CAC	1220 bp
		Sw-SaV-Ell-F4	CAG TGA TAG GTC TTC CAT CG	1210 bp
		Sw-SaV-Ell-F5	CAA TTC CGG AAC CGC YGT GTC	1230 bp
		Sw-SaV-Ell-F6	GCA CTA GCC TGA GTG AYA GCA CC	1220 bp
		Sw-SaV-Ell-F7	ACT AGC CTG GCT GAC AAC ACC	1220 bp
		Sw-SaV-Ell-F8	CTT GTG TGG CCC AGG TTG TT	1370 bp
		Sw-SaV-Ell-F9	GGT TTG GCA GTG TTT CAG CTG G	1330 bp
		Sw-SaV-Ell-F10	GCC AGT ACC GCC ATT AAC AAC G	1200 bp
Norovirus (NoV)	3'RACE PCR	*Sw-NoV-GII.11-Ell-F*	TTG TGG CCA CTG TGG AGA GC	1330 bp
		Sw-NoV-GII.18-Ell-F	CAG TTG AAT TTG TGA CAG AGT CA	1390 bp
All viruses	3'RACE-RT	OligodT-Anchor-Adapter	GCG CGC GCC ACC AAT TTA AA T(15)V	-
	3'RACE-PCR	Adapter-1*	GCG CGC GCC ACC AAT TTA AAT	-

Table S10: List and features of oligonucleotide primers used for the verification 3'RACE RT-PCR reactions of selected sapovirus (SaV) and norovirus (NoV) consensus sequences. ID:identification name.
